# Genome Scan for Selection in Structured Layer Chicken Populations Exploiting Linkage Disequilibrium Information

**DOI:** 10.1371/journal.pone.0130497

**Published:** 2015-07-07

**Authors:** Mahmood Gholami, Christian Reimer, Malena Erbe, Rudolf Preisinger, Annett Weigend, Steffen Weigend, Bertrand Servin, Henner Simianer

**Affiliations:** 1 Animal Breeding and Genetics Group, Department of Animal Sciences, Georg-August-University Göttingen, Göttingen, Germany; 2 LOHMANN Tierzucht GMBH, Cuxhaven, Germany; 3 Institute of Farm Animal Genetics (ING), Friedrich-Loeffler-Institut (FLI), Neustadt, Germany; 4 Laboratoire Génétique, Physiologie et Systèmes d’Elevage, Institut National de la Recherche Agronomique, Castanet-Tolosan, France; Sun Yat-sen University, CHINA

## Abstract

An increasing interest is being placed in the detection of genes, or genomic regions, that have been targeted by selection because identifying signatures of selection can lead to a better understanding of genotype-phenotype relationships. A common strategy for the detection of selection signatures is to compare samples from distinct populations and to search for genomic regions with outstanding genetic differentiation. The aim of this study was to detect selective signatures in layer chicken populations using a recently proposed approach, hapFLK, which exploits linkage disequilibrium information while accounting appropriately for the hierarchical structure of populations. We performed the analysis on 70 individuals from three commercial layer breeds (White Leghorn, White Rock and Rhode Island Red), genotyped for approximately 1 million SNPs. We found a total of 41 and 107 regions with outstanding differentiation or similarity using hapFLK and its single SNP counterpart FLK respectively. Annotation of selection signature regions revealed various genes and QTL corresponding to productions traits, for which layer breeds were selected. A number of the detected genes were associated with growth and carcass traits, including *IGF-1R*, *AGRP* and *STAT5B*. We also annotated an interesting gene associated with the dark brown feather color mutational phenotype in chickens (*SOX10*). We compared FST, FLK and hapFLK and demonstrated that exploiting linkage disequilibrium information and accounting for hierarchical population structure decreased the false detection rate.

## Introduction

A local reduction of genetic variation up- and downstream of the beneficial mutation is caused by the rapid fixation of a beneficial mutation, leaving special patterns of DNA behind, which is commonly referred to as a “selective sweep” [1]. The study of such signatures of selection can provide valuable insights into genomic regions harboring interesting genes that are or have been under selective pressure and hence can help to understand the mechanisms that led to the differentiation of various genotypes and their influenced phenotypes during selection. Recently, an increasing interest has been placed in the detection of genes, or genomic regions, that are targeted by selection [2], permitted by the availability of large-scale SNP datasets that allow to scan the genome for positions that may have been targets of recent selection [3].

Many different methods are available for detecting selective sweeps from DNA sequence data. Qanbari *et al*. (2014) [4] classified these methods in two main groups: intra-population statistics (e.g. Kim and Nielsen (2004) [5] and Sabeti *et al*. (2002) [3]) and inter-populations statistics (e.g. Lewontin and Krakauer (1973) [6] and Beaumont and Balding (2004) [7]). Innan and Kim (2008) [8] and Yi *et al*. (2010) [9] showed that between recently diverged populations, inter-populations statistics have more statistical power for the detection of selection signatures. These methods are particularly suited for studying species that are structured in well-defined populations, which is the case in many domesticated species.

Inter-populations statistics can be divided into two groups based on single site or haplotype differentiation analyses [4]. The most widely used single site differentiation statistic is Wright’s fixation index, F_ST_ [10]. A major concern with Wright’s F_ST_ is that it implicitly assumes that populations have the same effective size (*N*_*e*_) and to be derived independently from an ancestral population. When this is not true F_ST_ will produce false positive signals, similar to the well-known effects of cryptic structure in genome-wide association studies [11]. Bonhomme *et al*. (2010) [12] proposed a new statistic, termed FLK, that deals with *N*_*e*_ variation and historical branching of populations by incorporating a population kinship matrix into the Lewontin and Krakauer (LK) [6] statistic and showed that FLK is indeed more powerful than F_ST_ for a given false positive rate.

Another group of methods builds on the fact that haplotype diversity and linkage disequilibrium (LD) patterns contain useful information for the detection of selection signatures [13] and therefore, usage of haplotype or LD based differentiation analyses has its own advantages. Browning and Weir (2010) [14] showed that SNP ascertainment bias has less impact on haplotype based differentiation analyses compared to single site differentiation. A major challenge regarding the haplotype differentiation scans is that it does not account for the possibility of hierarchical structure between populations. Therefore Fariello *et al*. (2013) [15] proposed the hapFLK statistic which is a haplotype based extension of the FLK statistic that accounts for both hierarchical structure and haplotype information. They showed that using haplotype information to detect selection in F_ST_-like approaches greatly increases the detection power. Specifically, they demonstrated that the hapFLK statistic has more power in detecting soft sweeps, incomplete sweeps and sweeps occurring in several populations.

The chicken is an excellent model for studying the signatures of selection under artificial breeding conditions due to growing genomic resources, the relatively rapid reproduction time and the existence of several inbred lines together with strong agricultural interest [16]. Several studies have investigated selection signatures in chicken either using sequence data or genotype data from low to medium density SNP chips. Rubin *et al*. (2010) [17] studied the signatures of domestication and selective sweeps using the “Pooled Heterozygosity” (H_P_) statistic in various commercial broiler and layer lines. Johansson *et al*. (2010) [18] explored the genomes of two lines of chickens subjected to 50 generations of divergent selection using a 60k SNP assay. Qanbari *et al*. (2012) [19] applied a modified sliding window, called “creeping window”, of H_P_ measures in pooled sequence data in laying chickens. In an earlier work we [20] studied the signatures of selection by F_ST_ in seven commercial breeds using approximately one million SNPs which, however, ignored the hierarchical structure of the populations analyzed. Recent divergence of certain commercial breeds [21] and the introduction of strong selection for production traits (in the 20th century) [22] fosters the interest in detecting selective sweeps in chicken using statistical methods that account for the strong hierarchical structure between these populations. Therefore, this dataset offers an interesting opportunity to evaluate methods that account for population structure in a setting characterized by a strong past selection pressure, high genetic drift and clear population structure, which has never been done before.

In this study, FLK [12] and hapFLK [15] statistics were applied on the same data as in our previous study on selection signatures in commercial chicken [20], allowing a comparison between F_ST_, FLK and hapFLK. In contrast to our previous work, the approaches used in the current study have the potential to identify genomic regions which have been selected more recently (e.g. soft sweeps) and are associated with specific layer traits.

## Materials and Methods

### Animals, Data collection and filtering

Two sets of samples—commercial egg layers and wild chicken (coded respectively LAY and ANC)—were used in this study. The commercial individuals from Lohmann Tierzucht GmbH originated from three different breeds. One commercial white egg layer breed based on White Leghorn (WL), with three separate lines, and the other two brown egg layer breeds based on White Rock (WR) and Rhode Island Red (RIR), respectively, each with two separate lines per breed. In each of these seven lines, ten individuals were sampled and genotyped. The wild chickens, comprising Red Jungle fowl (Cochin-Chinese) (*G*. *g*. *gallus*) and Red Jungle fowl (Burmese) (*G*. *g*. *spadiceus*) were sampled within the AVIANDIV project. A more detailed list of breeds is presented in Table 1. The ANC group consisted of two subspecies of *Gallus gallus* that are believed to stem in straight line from wild ancestors of domestic chickens. Data is publicly available (S1 Dataset).

**Table 1:** Name, abbreviation, number of individuals and the egg color for each breed used in this study.

Breed	Abbreviation	# of lines	# of individuals	Egg color
White Leghorn	WL(1/2/3)	3	30(0♂,30♀)	White
Rhode Island Red	RIR(1/2)	2	20(2♂,18♀)	Brown
White Rock	WR(1/2)	2	20(2♂,18♀)	Brown
Gallus gallus gallus	ANC/Ggal	1	2(0♂,2♀)	Brown
Gallus gallus spadiceus	ANC/Gspa	1	2(0♂,2♀)	Brown

Genotyping was done with three Affymetrix 600K SNP arrays. Overlapping SNPs between the three 600K SNP arrays were removed by the data provider and a total of 1,139,073 SNPs remained. For this study we included only the SNPs that were located on autosomal chromosomes (1–28), SNPs that were located on sex chromosomes and linkage groups were removed (62,337 were removed). SNPs with at least one missing value and SNPs with minor allele frequencies lower than 5% (172,344 SNPs) were removed in order to avoid dealing with genotyping errors; this approach was suggested by the data provider. A total of 904,392 SNPs remained after filtering. The entire filtering process was done using the PLINK software (http://pngu.mgh.harvard.edu/purcell/plink/) [23].

### Population structure analysis

Using Reynolds’ genetic distances [24], a phylogenetic tree was constructed to retrieve the structure of the studied samples.

### FLK and hapFLK calculation

To identify regions under selection, FLK and hapFLK were calculated in all LAY breeds, using ANC individuals for rooting the population tree. FLK calculates variation of the inbreeding coefficient and incorporate hierarchical structure by using a population kinship matrix (for details see Bonhomme *et al*. (2010) [12]). The same matrix is used in hapFLK, but the statistic is computed from haplotype frequencies rather than SNP allele frequencies. Here, the haplotypes considered are in fact latent states extracted from the multipoint linkage disequilibrium model of Scheet and Stephens [25] (for details read Fariello *et al*. (2013) [15]). To determine the number of underlying latent states we used the fastPHASE [25] cross validation procedure, which indicated that 5 or 10 haplotype clusters were adequate. We found that using either 5 or 10 haplotype clusters gave nearly identical results and therefore present those obtained assuming 5 haplotype clusters.

### Assigning signatures of selection to specific population groups

When using differentiation-based approaches, it is sometimes difficult to pinpoint the population(s) that have been the target of selection. Fariello *et al*. (2013) [15] proposed to decompose the hapFLK statistic by projecting it on principal components (PC) of the population kinship matrix to identify which part of the population tree exhibits an outlying differentiation in a particular genomic region. Here, we employed this approach to look for selection signatures that affected either (i) the whole population set (LAY), (ii) white layer populations or (iii) brown layer populations. For (i) we used the hapFLK statistic, for (ii) and (iii) we considered the projection of the statistic on the subtree corresponding to white (resp. brown) layer populations. In each case we considered that a position lying in the top and bottom 0.05% of the empirical distribution was potentially within a selection signature.

For each selection signature, we then re-estimated the branch lengths of the population tree, using local allele or haplotype clusters frequencies (see Fariello *et al*. (2013) [15] for details) and identified the branch lengths that seem significantly larger than the branches of whole genome tree to pinpoint selected populations.

### Fitting of gamma distribution

As hapFLK statistic does not follow a known distribution under neutrality, the null distribution has to be estimated from the data. As hapFLK is similar to FLK, a good approximation to the asymptotic distribution of hapFLK comes from the gamma distribution family. To estimate p-values of selection signatures, we fitted a gamma distribution to the hapFLK observed distribution, using the minimum distance estimation method [26,27] which is robust to outliers, which helps to reduce the influence of selection signatures in estimating the null distribution. This was done for false detection rate (FDR) estimation.

### Annotation

As explained above, regions with extreme FLK and hapFLK values were considered as candidates for selective sweeps. For all the three groups (all layers, white layers and brown layers) the extreme values (the upper and lower 0.05%) that were within 500 kb of each other were grouped together. For all joined groups gene annotations, QTL annotations and pathway annotations were completed. Gene annotations were done with the biomaRt R package [28] based on the Ensembl database [29] of Gallus_gallus-4.0 assembly. Animal QTL database [30] was used for QTL annotation, KEGG database for pathway annotation [31] and Gene Ontology (GO) database for GO annotation [32]. Gene enrichment analysis was done with Fisher’s exact test [33] for all annotated genes in all groups (all layers, white layers and brown layers) separately. Pathways and gene ontologies with p ≤ 0.05 were identified as being under selection.

## Results

### Population structure

**Fig 1:**
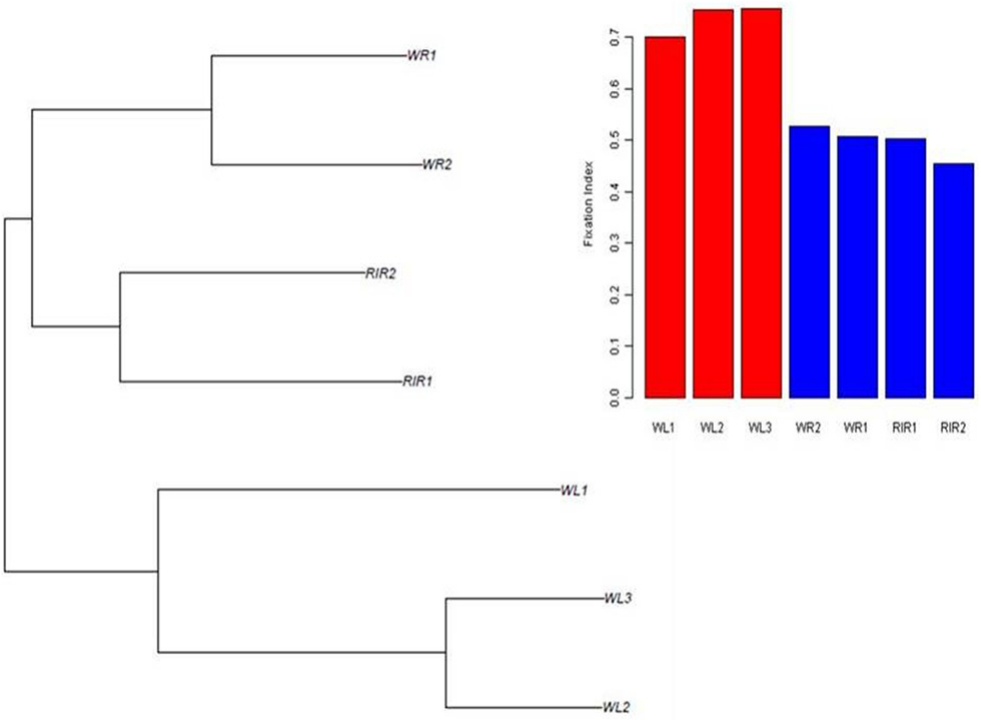
Reynolds’ genetic distances population tree of seven commercial breeds and histogram of fixation index for each line.

A phylogenetic tree based on Reynolds’ genetic distances with 100,000 randomly selected SNPs (100 replications) was constructed and is shown in Fig 1. As Fig 1 shows, commercial white egg-layer breeds were separated from brown egg-layers and grouped in one sub-tree. In the sub-trees, the two white-layer lines WL2 and WL3 as well as the two brown-layer lines WR1 and WR2 form a separate sub-cluster, respectively. The population specific fixation indices of all populations, also shown in Fig 1, are extremely high (ranging from 0.45 to 0.75), reflecting the very strong effect of genetic drift in these populations, with the three White Leghorn populations notably more inbred than the Brown layer populations.

### FLK

**Fig 2:**
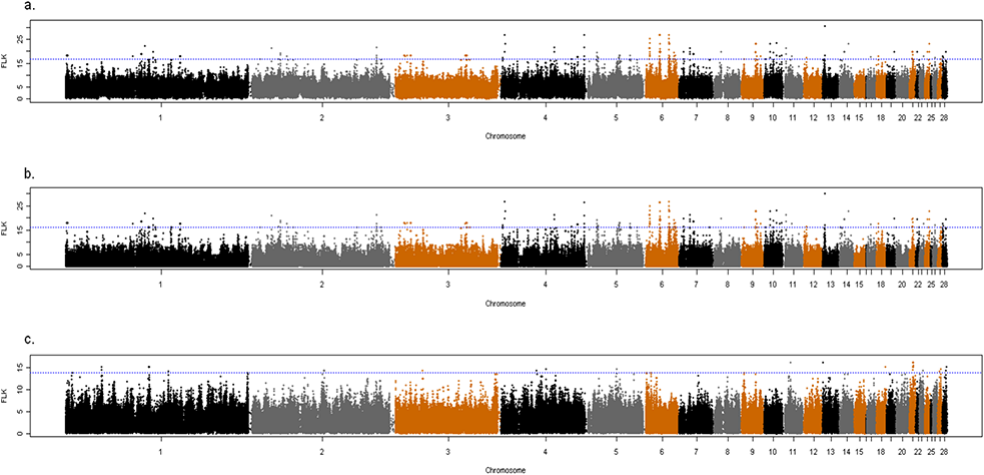
Manhattan plot of FLK analysis over the entire genome. Blue line indicates the upper 0.05% of FLK distribution, for (A) within all breeds, (B) within white breeds, and, (C) within brown breeds.

Based on the FLK values distribution, a total of 107 regions (63 in all layers, 27 in white layers and 17 in brown layers) were detected as signatures of selection (S1 Table). All these regions were in the upper 0.05% of the distribution which is representative of regions with fixed difference between populations. The genome-wide distribution of FLK values obtained from each group—all, white and brown—are depicted in Fig 2A, 2B and 2C, respectively. Annotation was carried out for all regions with extreme FLK values, i.e. potential selection signatures. The lists of genes in selective sweeps detected with FLK are available in the supplementary tables (S2, S3 and S4 Tables). The annotation list is enriched with genes of biological interest involved in various pathways such as ATP metabolic process (P = 0.023), metal ion binding (P = 0.001), nucleic acid binding (P = 0.008) and metabolic pathways (P<0.001), all of which can be related to production traits under selection in layers. The lists of pathways and gene ontologies under selection are available in supplementary tables (S5, S6 and S7 Tables). We identified three candidate genes which can be related to the breeding goals of chickens. *H3F3C* and *AGRP* which are associated with body growth and body weight [34,35], and *IL19* which is associated to the immune system in chicken [36]. More details about gene locations and study groups are available in Table 2. We also detected several QTL overlapping selection signatures for traits such as breast muscle weight, abdominal fat weight and liver weight, which all are related to the breeding goals of chickens (Table 3).

**Table 2:** Genes associated with productive traits in FLK and hapFLK analysis in all three studies. ‘All’, ‘White’, and ‘Brown’ stand for inclusion of all the commercial breeds, analysis within white layers and analysis within brown layers, respectively. ‘s’ stands for similarity and ‘d’ for difference.

Chr	Gene	Function	Test	Group
1	SOX10	Causal mutation underlying the dark brown mutational phenotype in chickens.	hapFLK	All(d) and Brown(d)
3	H3F3C	Potential role in early feed stress responses and adaptation to feed intake stress.	FLK	All(d), White(d)
10	IGF-1R	Associated with chicken early growth and carcass traits.	hapFLK	Brown(s)
11	AGRP	Associated with chest width, body weight, and high slaughter rate.	FLK	All(d), White(d)
20	BPIFB8	A molecular actor of the avian egg natural defense.	hapFLK	Brown(s)
26	IL19	Associated with immunoprotection.	FLK	All(d), White(d)
27	STAT5B	A potential genetic marker for growth and reproduction traits.	hapFLK	Brown(s)

**Table 3:** QTL associated with productive traits in FLK analysis in all three studies. ‘All’ stands for inclusion of all commercial breeds, and ‘White’ for analysis within white layers.

Chr	QTL	Group
1	Fear-tonic immobility duration	All, White
4	Disease-related traits	All, White
5	Disease-related traits	All, White
6	Liver weight	All, White
11	Breast muscle weight	All, White
26	Abdominal fat weight	All, White
26	Abdominal fat percentage	All, White

### hapFLK

**Fig 3:**
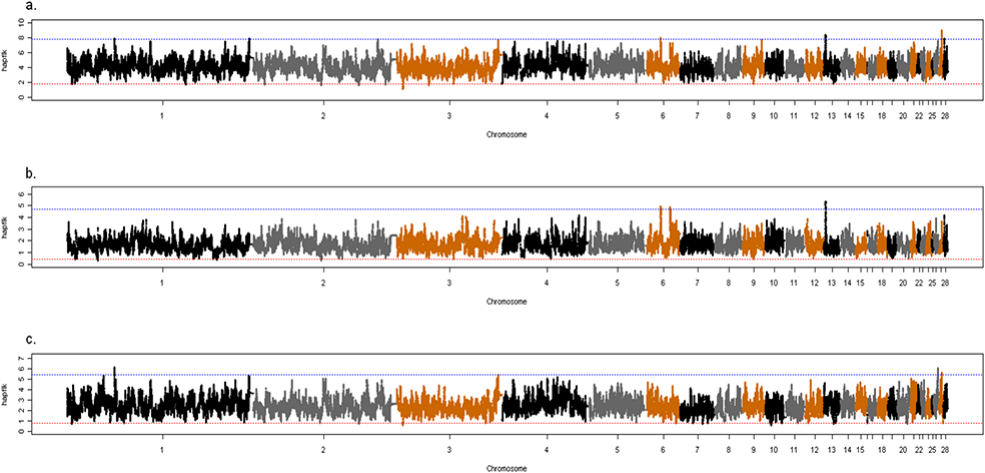
Manhattan plot of hapFLK analysis over the entire genome with 5 clusters. Blue (red) line indicates the upper (lower) 0.05% of hapFLK distribution, for, (A) within all breeds, (B) within white breeds, and, (C) Within brown breeds.

Based on the hapFLK values distribution, a total of 41 regions (17 in all layers, 12 in white layers and 12 in brown layers) were detected as selection signatures (S8 Table). All these regions were in either the upper or the lower 0.05% of the distribution, which represent regions with a fixed difference or fixed similarity between populations, respectively. The genome-wide distribution of hapFLK values with 5 haplotype clusters obtained for each group—all, white and brown—are depicted in Fig 3A, 3B and 3C, respectively. Annotation was carried out for all regions with extreme hapFLK values, i.e. potential selective sweeps. The lists of genes for selective sweeps detected with hapFLK are available in the supplementary tables (S9, S10 and S11 Tables). The annotation list is enriched with genes of biological interest involved in various pathways such as nerve development (p = 0.027), growth factor receptor (p = 0.008), RNA metabolic process (p = 0.042) and skeletal muscle cell differentiation (p = 0.032), all of which could be related to production traits indirectly. The lists of pathways and gene ontologies under which were detected under selection in this study are available in the supplementary tables (S12, S13 and S14 Tables). We identified four genes that were related to the breeding goals of chickens with the hapFLK method. *IGF-1R* and *STAT5B* are associated with growth and carcass traits [37,38]. *BPIFB8* and *SOX10*, which are associated with egg natural defense [39] and dark brown mutational phenotype [40] respectively (more details is available in Table 2). Several QTL, which were related to the breeding goals of egg-layer chickens were detected as well, for traits such as drumstick and thigh morphology, carcass weight and shank length. A complete list of all QTL with more details is available in Table 4.

**Table 4:** QTL associated with productive traits in hapFLK analysis in all three studies. ‘All’, ‘White’, and ‘Brown’, stand for inclusion of all the commercial breeds, analysis within white layers and analysis within brown layers, respectively. ‘s’ stands for similarity and ‘d’ for difference.

Chr	QTL	Group
1	Abdominal fat percentage	All(d), Brown(d) and White(d)
1	Heart weight	All(s) and Brown(s)
2	Carcass weight	All(s) and Brown(s)
2	Drumstick and thigh weight	All(s) and Brown(s)
2	Drumstick and thigh muscle weight	All(s) and Brown(s)
2	Shank length	All(s) and Brown(s)
2	Shank circumference	All(s) and Brown(s)
2	Heart weight	White(s)
9	Liver percentage	White(s)

## Discussion

### Structure analysis and P_0_ comparison

Our population structure analyses are largely in agreement with the expected historical origin of the breeds [21] and as expected, they are also similar to the previous study using the same data [20].

One of the issues in the FLK and hapFLK analysis in this study is using only 4 wild chickens for development of the population's kinship matrix. We assessed whether using a different set of outgroup individuals could possibly change our findings by verifying the influence of the outgroup set on the estimation of the ancestral allele frequency (p_0_). p_0_ can be seen as a nuisance parameter in the model that has to be estimated from the data through the kinship matrix. We studied the possible impact of the number of wild chickens used by comparing p_0_ when being calculated from 4 wild chickens (our ANC group) vs. 40 wild chickens (consisted of 20 *Gallus gallus gallus* and 20 *Gallus gallus spadiceus* which were genotyped with Axiom Genome-Wide Chicken Genotyping Array of Affymetrix and were available only for this comparison). p_0_ was calculated for each group (ANC group and 40 wild chickens) for every SNP on the 600K SNP chip. Pairwise comparison of each group’s p_0_ values along the genome gave an average correlation of 0.95. This high correlation suggests that there is no vital difference in development of population's kinship matrix with 4 or 40 wild chickens. Therefore the kinship matrix calculated based on four wild chickens, which had been genotyped for the complete set of close to one million SNPs was considered sufficient. A histogram of the differences in p_0_ estimated with the two outgroup sets is shown in S1 Fig, showing that more than 90% of the differences are less than ± 0.02.

### Fitting of gamma distribution

Although the outlier approach is an effective and widely used method for identification of genes under selection lacking known phenotypes [41], an outlier signal is not necessarily synonymous with regions being under selection [42]. Therefore we fitted a gamma distribution to the hapFLK in order to estimate the false discovery rate (FDR). This approach suggested an FDR of 10–20% in our analysis.

### F_ST_, FLK and hapFLK

**Fig 4:**
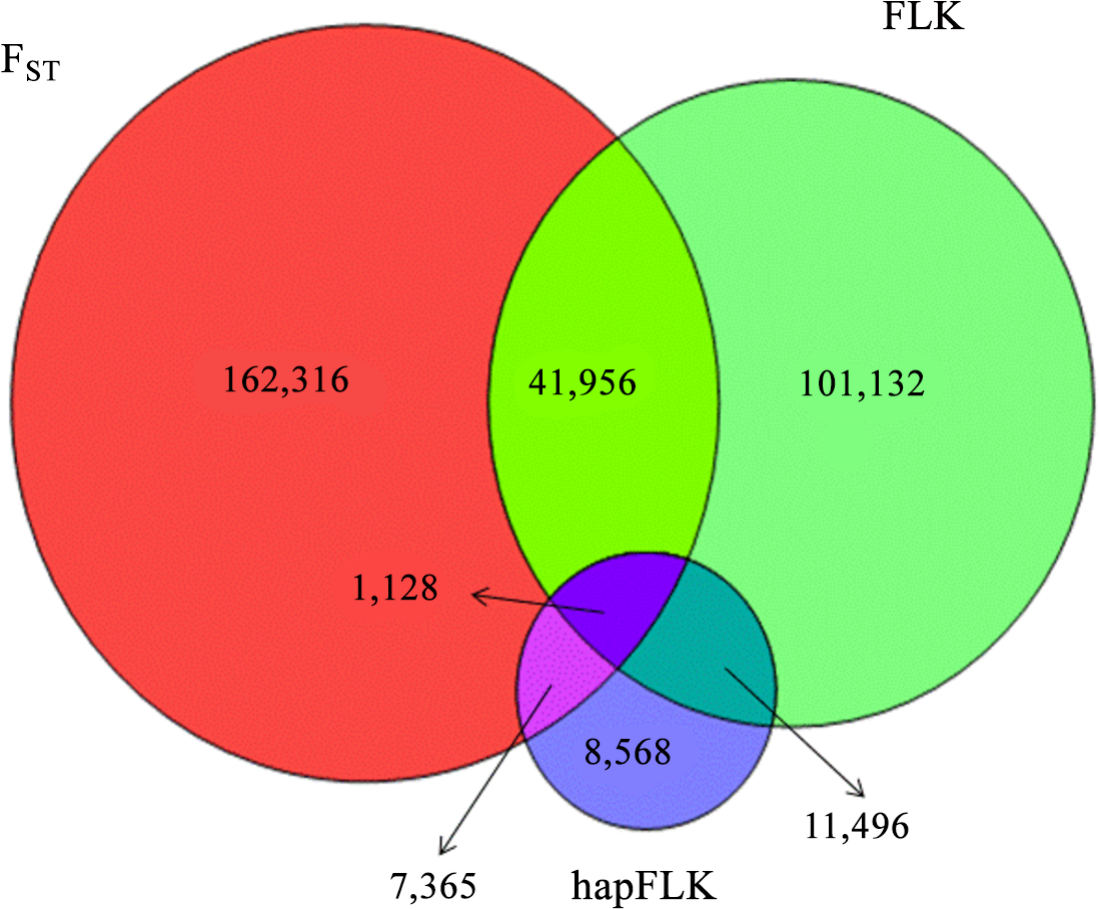
Venn diagram of overlapping SNPs identified as under selection, with F_ST_, FLK and hapFLK methods using same threshold (upper and lower 1%).

An overlap exists between the regions that have been determined as regions under selection in a previous study with F_ST_ [20] and the current analysis of FLK and hapFLK as shown by the Venn diagram for the number of SNPs identified as being under selection with either of the methods shown in Fig 4. Using the same threshold as in our previous work [20] (upper and lower 1%) resulted in detection of a lower number of selection signatures with FLK (73.2%) and much lower with hapFLK (13.4%) compared to the F_ST_ based results reported in our earlier study on the same data (list of regions detected with F_ST_ method is available in S15 Table) [20]. A finding suggested that ten-thousands of polymorphisms respond to selection, which was the case in our earlier work [20], does not appear realistic [43]. Many of the outliers detected with F_ST_ must be considered as false positives, which might be partly due to the fact that the method assumes populations to have the same effective size and to have emerged independently from the same ancestral population. Therefore we used a much stricter threshold (upper and lower 0.05%) in the study presented here than in our previous work (upper and lower 1%) [20]. Accordingly, the use of a stricter threshold and the application of methods that account for different effective population sizes and hierarchical phylogenies (FLK and hapFLK), resulted in the detection of much lower number of selection signatures. There is also an overlap between regions detected by hapFLK and FLK (44.2%) which is due to the use of same statistic in both methods. The difference between regions detected by FLK and hapFLK can be due to the fact that haplotype and SNPs harbor different information.

**Fig 5:**
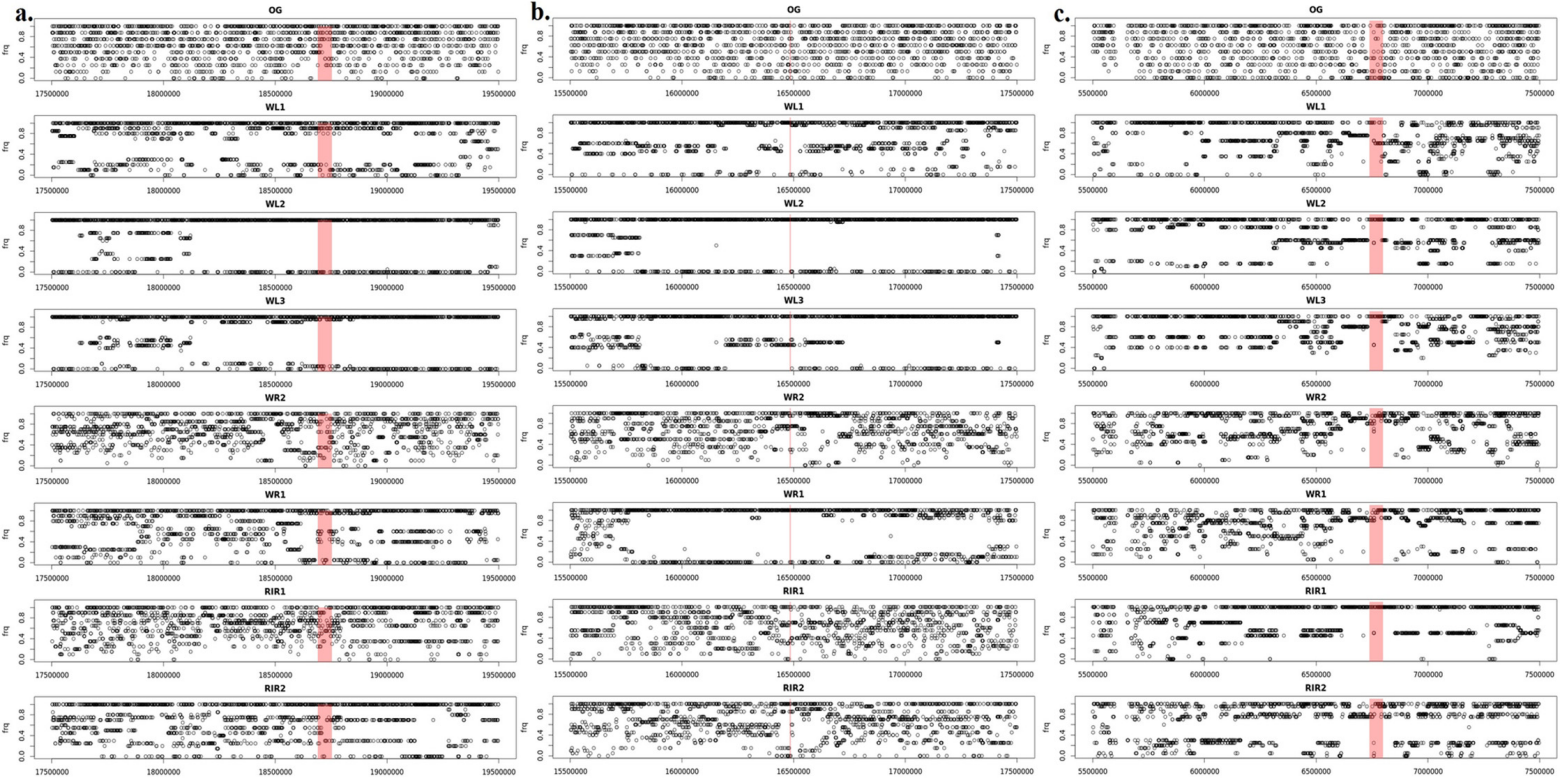
Allele frequency in different breeds for 2 Mbp around the intended region. Red box indicates, for (A) *TGFB2* gene (Chr3: 18,690,003–18,753,123), (B) *H3F3C* gene (Chr3: 16,483,162–16,487,393) and (C) 60Kb region on chromosome 10 (6,799,776–6,738,610).

As an example, in Fig 5A we demonstrate allele frequencies at SNP positions around the *TGFB2* gene (Chr3: 18,690,003–18,753,123) which was detected as a gene under selection by F_ST_ [20] due to a reduction of diversity within the WL breed. However, since this reduction exists only within the WL breed this can also be explained by drift alone. By taking the population tree into consideration, FLK does not detect any signals in this region. Another example is the region around the *H3F3C* gene (Chr3: 16,483,162–16,487,393) which was detected to be under selection by FLK. Allele frequencies around this region shows that a huge diversity exists between some breeds (Fig 5B). We detect an outlier with FLK in particular because WR1 and WR2 show very different patterns of allele frequencies in this region although they are closely related in the population tree. However F_ST_ was not able to detect any signal here, since F_ST_ treats each population as an independent evidence for sweep detection and does not consider the huge difference between WL, RIR and WR breeds. There are as well cases in which all three methods (F_ST_, FLK and hapFLK) were able to detect the region under selection. An example is a 60Kb region on chromosome 10 (6,799,776–6,738,610). Fig 5C shows allele frequencies around this region.

A complete hard sweep is expected to be large [44], while a soft sweep is more likely to have smaller size [45]. In the current study we detected smaller sweeps (bp length) compared to our F_ST_ study, which may be due to the fact that hapFLK has greater power in detection of soft sweeps. Nevertheless we should as well take into account the false positive rate of our F_ST_ study. A boxplot of sweep size with F_ST_ and hapFLK method is shown in S2 Fig.

A vast majority of differentiated polymorphisms in our data set could be caused by genetic drift. Genetic drift is high when the (effective) population size is small [46] which is the case in commercial laying breeds [47]. Since regions differentiated by selection and regions differentiated by drift alone may overlap, there is a lack of power in our analysis. This could be solved by using a larger number of populations to minimize the risk that a systematic pattern of differentiation in many breeds (say, several white layers vs. several brown layers) is created at random by drift alone. Other obstacles in this study are the use of only 10 animals per sample and filtering for minor allele frequencies; these two issues might have an effect on the estimation of allele frequencies, comparison of rarer alleles and identification of all haplotypes. In a recent simulation study [48] it was shown that the power of most selection signature tests is more dependent on marker density than on sample size, and that with a marker density similar to the one used in the present study a high power and positional resolution was achieved with 15 sampled individuals per population. We detected several genes related to the breeding goals of egg-layer chickens, such as low body weight, high reproduction performance and good feed conversion [49], both with FLK and hapFLK. For instance, with the FLK method we detected several QTL associated to disease-related traits and breast muscle weight, as well as *AGRP* (agouti related protein homolog), which is associated with breast muscle water loss rate, chest width, body weight, slaughter rate and semi-evisceration weight [35].

In the hapFLK analysis, we also detected several genes, which are associated with growth and carcass traits, such as *IGF-1R* and *STAT5B*. *STAT5B* (signal transducer and activator of transcription 5B) is associated with growth and reproduction traits [38]. *IGF-1R* (insulin-like growth factor 1) is similar to *IGF2* [50], which was detected in our previous work [20]. *IGF-1R* is associated with chicken early growth and carcass traits [37]. We additionally detected several QTL associated to carcass weight, drumstick weight and shank length. QTL associated with meat production, as well as both *IGF-1R* and *STAT5B*, were located in regions that were similar between brown layers. Supporting results were found in our previous study [20], where we detected genes associated to meat quality and production in brown layers, which reflects the fact that brown egg-layers were originally a dual-purpose breed [21].

**Fig 6:**
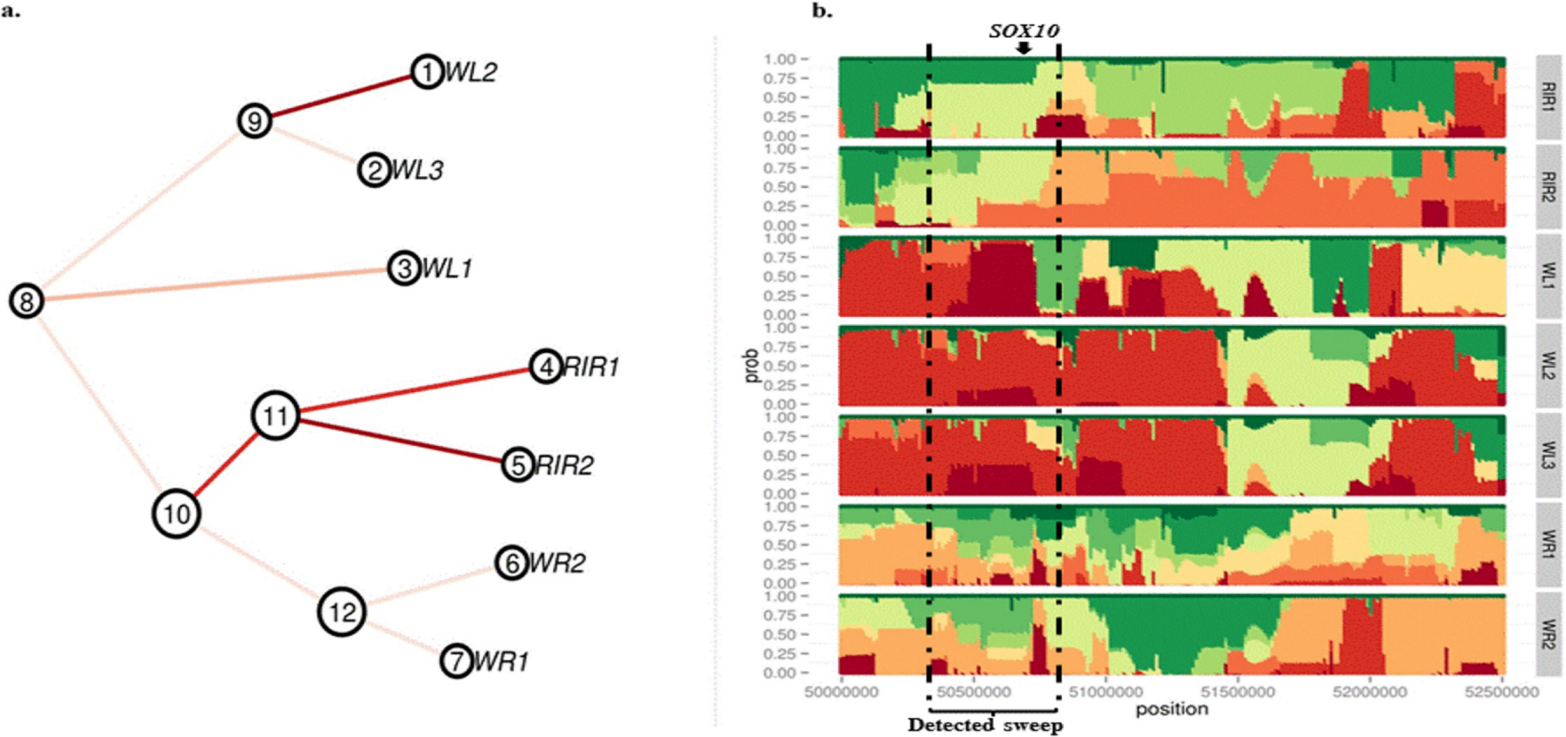
(A) Re-estimation of local tree using haplotype clusters frequencies for surrounding region of *SOX10* gene. (B) Haplotype frequencies for the surrounding region of *SOX10* gene (50.8 Mbp).

Bonhomme *et al*. (2010) [12] and Fariello *et al*. (2013) [15] showed with simulation that using FLK or hapFLK method to detect selection signatures in comparison to other F_ST_-like approaches greatly increases the detection power. Specifically, hapFLK statistic has more power in detecting sweeps occurring in several populations. Due to this, we were able to detect *SOX10* with hapFLK which was not detected by F_ST_ or FLK method. *SOX10* is a gene on chromosome one underlying the dark brown mutational phenotype in chickens plumage [40]. *SOX10* was detected in regions that were different between brown layers. Re-estimation of the local tree using haplotype clusters frequencies (Fig 6A) and haplotype frequencies (Fig 6B) for the region surrounding *SOX10* revealed selection in the RIR breeds in this region. RIR is the only breed with dark brown feather in our data set [51], which is in great agreement with our selection signature detection.

### Conclusions

In conclusion we were able to identify several putative selection signature regions with genes corresponding to the traits associated to growth and reproduction traits. Some of these annotated genes were similar (or had similar functions) to our findings in our previous work [20]. However, several of the detected regions were not associated with any genes related to production traits, which could be due to insufficient knowledge about these regions [52]. We did not identify selection signatures that were reported in other studies on chicken [17,53] which could be due to lack of diversity in our data compared to their data set. By detection of *SOX10* as a gene under selection, we demonstrated that the use of haplotype frequencies and consideration of hierarchical structure can improve the power of detection of soft sweep in our data set.

## Supporting Information

S1 Fig Histogram of p_0_ difference between the calculation with 4 wild chickens (ANC group) and 40 wild chickens. (TIFF)

S2 Fig Boxplot of sweep size with F_ST_ and hapFLK method. (TIFF)

S1 Table Regions detected as putative selective sweeps detected with FLK with upper 0.05% threshold. All, White and Brown stands for studies with all layers, within white layers and within brown layers, respectively.
(PDF)

S2 Table List of genes for selective sweeps detected with FLK with 0.05% threshold in all layers. (PDF)

S3 Table List of genes for selective sweeps detected with FLK with 0.05% threshold in white layers. (PDF)

S4 Table List of genes for selective sweeps detected with FLK with 0.05% threshold in brown layers. (PDF)

S5 Table Lists of pathways and gene ontologies under selection with FLK with 0.05% threshold in all layers. (PDF)

S6 Table Lists of pathways and gene ontologies under selection with FLK with 0.05% threshold in white layers. (PDF)

S7 Table Lists of pathways and gene ontologies under selection with FLK with 0.05% threshold in brown layers. (PDF)

S8 Table Regions detected as putative selective sweeps detected with hapFLK with upper (U) and lower (L) 0.05% threshold. All, White and Brown stands for studies with all layers, within white layers and within brown layers, respectively.
(PDF)

S9 Table List of genes for selective sweeps detected with hapFLK with 0.05% threshold in all layers. (PDF)

S10 Table List of genes for selective sweeps detected with hapFLK with 0.05% threshold in white layers. (PDF)

S11 Table List of genes for selective sweeps detected with hapFLK with 0.05% threshold in brown layers. (PDF)

S12 Table Lists of pathways and gene ontologies under selection with hapFLK with 0.05% threshold in all layers. (PDF)

S13 Table Lists of pathways and gene ontologies under selection with hapFLK with 0.05% threshold in white layers. (PDF)

S14 Table Lists of pathways and gene ontologies under selection with hapFLK with 0.05% threshold in brown layers. (PDF)

S15 Table Regions detected as putative selective sweeps detected with F_ST_ with upper (U) and lower (L) 1% threshold. LG stands for studies between commercial-layers and non-commercial chickens and BW stands for studies between brown and white layers, respectively.
(PDF)

S1 Dataset Compress file of genotyped data in plink format. (RAR)

## References

[1] Kaplan NL, Hudson RR, Langley CH (1989) The “hitchhiking effect” revisited. Genetics 123: 887–899. 2612899 10.1093/genetics/123.4.887 PMC1203897

[2] Nielsen R (2005) Molecular Signatures of Natural Selection. Annu Rev Genet 39: 197–218. 10.1146/annurev.genet.39.073003.112420 16285858

[3] Sabeti PC, Reich DE, Higgins JM, Levine HZP, Richter DJ, Schaffner SF, et al (2002) Detecting recent positive selection in the human genome from haplotype structure. Nature 419: 832–837. 10.1038/nature01140 12397357

[4] Qanbari S, Simianer H (n.d.) Mapping signatures of positive selection in the genome of livestock. Livest Sci. Available: http://www.sciencedirect.com/science/article/pii/S187114131400239X. Accessed: 18 June 2014.

[5] Kim Y, Nielsen R (2004) Linkage Disequilibrium as a Signature of Selective Sweeps. Genetics 167: 1513–1524. 10.1534/genetics.103.025387 15280259 PMC1470945

[6] Lewontin RC, Krakauer J (1973) Distribution of Gene Frequency as a Test of the Theory of the Selective Neutrality of Polymorphisms. Genetics 74: 175–195. 4711903 10.1093/genetics/74.1.175 PMC1212935

[7] Beaumont MA, Balding DJ (2004) Identifying adaptive genetic divergence among populations from genome scans. Mol Ecol 13: 969–980. 15012769 10.1111/j.1365-294x.2004.02125.x

[8] Innan H, Kim Y (2008) Detecting Local Adaptation Using the Joint Sampling of Polymorphism Data in the Parental and Derived Populations. Genetics 179: 1713–1720. 10.1534/genetics.108.086835 18562650 PMC2475763

[9] Yi X, Liang Y, Huerta-Sanchez E, Jin X, Cuo ZXP, Pool JE, et al (2010) Sequencing of 50 Human Exomes Reveals Adaptation to High Altitude. Science 329: 75–78. 10.1126/science.1190371 20595611 PMC3711608

[10] Wright S (1949) The Genetical Structure of Populations. Ann Eugen 15: 323–354. 10.1111/j.1469-1809.1949.tb02451.x 24540312

[11] Price AL, Zaitlen NA, Reich D, Patterson N (2010) New approaches to population stratification in genome-wide association studies. Nat Rev Genet 11: 459–463. 10.1038/nrg2813 20548291 PMC2975875

[12] Bonhomme M, Chevalet C, Servin B, Boitard S, Abdallah J, Blott S, et al (2010) Detecting Selection in Population Trees: The Lewontin and Krakauer Test Extended. Genetics 186: 241–262. 10.1534/genetics.110.117275 20855576 PMC2940290

[13] Sabeti PC, Varilly P, Fry B, Lohmueller J, Hostetter E, Cotsapas C, et al (2007) Genome-wide detection and characterization of positive selection in human populations. Nature 449: 913–918. 10.1038/nature06250 17943131 PMC2687721

[14] Browning SR, Weir BS (2010) Population Structure With Localized Haplotype Clusters. Genetics 185: 1337–1344. 10.1534/genetics.110.116681 20457877 PMC2927760

[15] Fariello MI, Boitard S, Naya H, SanCristobal M, Servin B (2013) Detecting signatures of selection through haplotype differentiation among hierarchically structured populations. Genetics 193: 929–941. 10.1534/genetics.112.147231 23307896 PMC3584007

[16] Brown WRA, Hubbard SJ, Tickle C, Wilson SA (2003) The chicken as a model for large-scale analysis of vertebrate gene function. Nat Rev Genet 4: 87–98. 10.1038/nrg998 12560806

[17] Rubin CJ, Zody MC, Eriksson J, Meadows JR, Sherwood E, Webster MT, et al (2010) Whole-genome resequencing reveals loci under selection during chicken domestication. Nature 464: 587–591. 10.1038/nature08832 20220755

[18] Johansson AM, Pettersson ME, Siegel PB, Carlborg Ö (2010) Genome-Wide Effects of Long-Term Divergent Selection. PLoS Genet 6: e1001188 10.1371/journal.pgen.1001188 21079680 PMC2973821

[19] Qanbari S, Strom TM, Haberer G, Weigend S, Gheyas AA, Turner F, et al (2012) A High Resolution Genome-Wide Scan for Significant Selective Sweeps: An Application to Pooled Sequence Data in Laying Chickens. PLoS ONE 7: e49525 10.1371/journal.pone.0049525 23209582 PMC3510216

[20] Gholami M, Erbe M, Gärke C, Preisinger R, Weigend A, Weigend S, et al (2014) Population Genomic Analyses Based on 1 Million SNPs in Commercial Egg Layers. PLoS ONE 9: e94509 10.1371/journal.pone.0094509 24739889 PMC3989219

[21] Crawford R (1990) poultry breeding and genetics Elsevier science.

[22] Burt DW (2005) Chicken genome: Current status and future opportunities. Genome Res 15: 1692–1698. 10.1101/gr.4141805 16339367

[23] Purcell S, Neale B, Todd-Brown K, Thomas L, Ferreira MAR, Bender D, et al (2007) PLINK: A Tool Set for Whole-Genome Association and Population-Based Linkage Analyses. Am J Hum Genet 81: 559–575. 17701901 10.1086/519795 PMC1950838

[24] Reynolds J, Weir BS, Cockerham CC (1983) Estimation of the Coancestry Coefficient: Basis for a Short-Term Genetic Distance. Genetics 105: 767–779. 17246175 10.1093/genetics/105.3.767 PMC1202185

[25] Scheet P, Stephens M (2006) A fast and flexible statistical model for large-scale population genotype data: applications to inferring missing genotypes and haplotypic phase. Am J Hum Genet 78: 629–644. 10.1086/502802 16532393 PMC1424677

[26] Clarke BR, McKinnon PL, Riley G (2012) A fast robust method for fitting gamma distributions. Stat Pap 53: 1001–1014. 10.1007/s00362-011-0404-3

[27] Zhou H, Alexander D, Lange K (2011) A quasi-Newton acceleration for high-dimensional optimization algorithms. Stat Comput 21: 261–273. 10.1007/s11222-009-9166-3 21359052 PMC3045213

[28] Durinck S (n.d.) biomaRt: Interface to BioMart databases (e.g. Ensembl, COSMIC, Wormbase and Gramene). R package version 260.

[29] Flicek P, Ahmed I, Amode MR, Barrell D, Beal K, Brent S, et al (2012) Ensembl 2013. Nucleic Acids Res 41: D48–D55. 10.1093/nar/gks1236 23203987 PMC3531136

[30] Hu Z-L, Park CA, Wu X-L, Reecy JM (2012) Animal QTLdb: an improved database tool for livestock animal QTL/association data dissemination in the post-genome era. Nucleic Acids Res 41: D871–D879. 10.1093/nar/gks1150 23180796 PMC3531174

[31] Kanehisa M, Goto S, Sato Y, Furumichi M, Tanabe M (2012) KEGG for integration and interpretation of large-scale molecular data sets. Nucleic Acids Res 40: D109–114. 10.1093/nar/gkr988 22080510 PMC3245020

[32] Ashburner M, Ball CA, Blake JA, Botstein D, Butler H, Cherry JM, et al (2000) Gene ontology: tool for the unification of biology. The Gene Ontology Consortium. Nat Genet 25: 25–29. 10.1038/75556 10802651 PMC3037419

[33] Fisher RA (1922) On the Interpretation of χ2 from Contingency Tables, and the Calculation of P. J R Stat Soc 85: 87–94. 10.2307/2340521

[34] Xu P, Denbow CJ, Meiri N, Denbow DM (2012) Fasting of 3-day-old chicks leads to changes in histone H3 methylation status. Physiol Behav 105: 276–282. 10.1016/j.physbeh.2011.06.023 21824486

[35] Bai Y, Sun G, Kang X, Han R, Tian Y, Li H, et al (2012) Polymorphisms of the pro-opiomelanocortin and agouti-related protein genes and their association with chicken production traits. Mol Biol Rep 39: 7533–7539. 10.1007/s11033-012-1587-y 22399312

[36] Kim S, Miska KB, McElroy AP, Jenkins MC, Fetterer RH, Cox CM, et al (2009) Molecular cloning and functional characterization of avian interleukin-19. Mol Immunol 47: 476–484. 10.1016/j.molimm.2009.08.027 19767108

[37] Lei M, Peng X, Zhou M, Luo C, Nie Q, Zhang X (2008) Polymorphisms of the IGF1R gene and their genetic effects on chicken early growth and carcass traits. BMC Genet 9: 70 10.1186/1471-2156-9-70 18990245 PMC2628351

[38] Zhao XH, Wang JY, Zhang GX, Wei Y, Gu YP, Yu YB (2012) Single nucleotide polymorphism in the STAT5b gene is associated with body weight and reproductive traits of the Jinghai Yellow chicken. Mol Biol Rep 39: 4177–4183. 10.1007/s11033-011-1202-7 21766180

[39] Gautron J, Réhault-Godbert S, Pascal G, Nys Y, Hincke MT (2011) Ovocalyxin-36 and other LBP/BPI/PLUNC-like proteins as molecular actors of the mechanisms of the avian egg natural defences. Biochem Soc Trans 39: 971–976. 10.1042/BST0390971 21787332

[40] Gunnarsson U, Kerje S, Bed’hom B, Sahlqvist A-S, Ekwall O, Tixier-Boichard M, et al (2011) The Dark brown plumage color in chickens is caused by an 8.3-kb deletion upstream of SOX10. Pigment Cell Melanoma Res 24: 268–274. 10.1111/j.1755-148X.2011.00825.x 21210960

[41] Narum SR, Hess JE (2011) Comparison of FST outlier tests for SNP loci under selection. Mol Ecol Resour 11: 184–194. 10.1111/j.1755-0998.2011.02987.x 21429174

[42] Akey JM (2009) Constructing genomic maps of positive selection in humans: Where do we go from here? Genome Res 19: 711–722. 10.1101/gr.086652.108 19411596 PMC3647533

[43] Nuzhdin SV, Turner TL (2013) Promises and limitations of hitchhiking mapping. Curr Opin Genet Dev 23: 694–699. 10.1016/j.gde.2013.10.002 24239053 PMC3872824

[44] Smith JM, Haigh J (1974) The hitch-hiking effect of a favourable gene. Genet Res 23: 23–35. 10.1017/S0016672300014634 4407212

[45] Pritchard JK, Pickrell JK, Coop G (2010) The Genetics of Human Adaptation: Hard Sweeps, Soft Sweeps, and Polygenic Adaptation. Curr Biol 20: R208–R215. 10.1016/j.cub.2009.11.055 20178769 PMC2994553

[46] Nielsen R, Slatkin M (2013) An introduction to population genetics: theory and applications Sunderland, Mass.: Sinauer Associates.

[47] Qanbari S, Hansen M, Weigend S, Preisinger R, Simianer H (2010) Linkage disequilibrium reveals different demographic history in egg laying chickens. BMC Genet 11: 103 10.1186/1471-2156-11-103 21078133 PMC3001694

[48] Ma Y, Ding X, Qanbari S, Weigend S, Zhang Q, Simianer H (2015) Properties of different selection signature statistics and a new strategy for combining them. Heredity 00, 1–11. 10.1038/hdy.2015.42 PMC4611237 25990878

[49] Muir WM, Aggrey SE (2003) Poultry Genetics, Breeding and Biotechnology. CABI. 724 p.

[50] Jiao S, Ren H, Li Y, Zhou J, Duan C, Lu L (2013) Differential regulation of IGF-I and IGF-II gene expression in skeletal muscle cells. Mol Cell Biochem 373: 107–113. 10.1007/s11010-012-1479-4 23054195

[51] Bassom F (2009) Mini encyclopedia of chicken breeds & care: a color directory of the most popular breeds and their care Bufflao, N.Y.; Richmond Hill, Ont.: Firefly Books.

[52] Eyras E, Reymond A, Castelo R, Bye JM, Camara F, Flicek P, et al (2005) Gene finding in the chicken genome. BMC Bioinformatics 6: 131 10.1186/1471-2105-6-131 15924626 PMC1174864

[53] Elferink MG, Megens H-J, Vereijken A, Hu X, Crooijmans RPMA, Groenen MA (2012) Signatures of Selection in the Genomes of Commercial and Non-Commercial Chicken Breeds. PLoS ONE 7: e32720 10.1371/journal.pone.0032720 22384281 PMC3287981

