# Supplementary figures and images for: Genome Scan for Selection in Structured Layer Chicken Populations Exploiting Linkage Disequilibrium Information
Source: PLoS One. 2015 Jul 7;10(7):e0130497. doi: 10.1371/journal.pone.0130497 (PMC4494984; doi:10.1371/journal.pone.0130497)

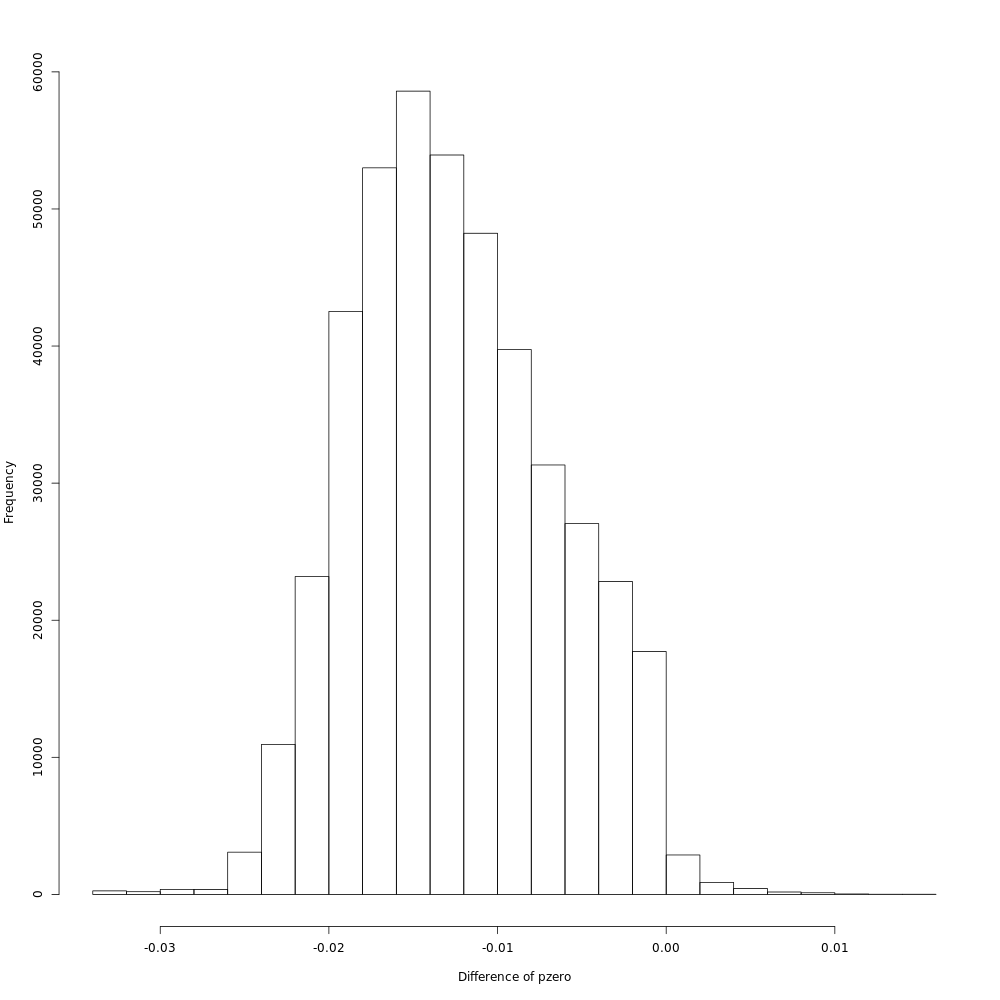

Supplement: S1 Fig — (TIFF) [file pone.0130497.s001.tiff]

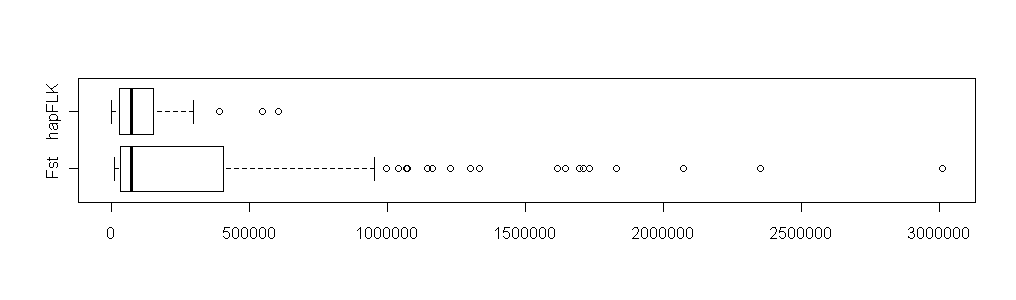

Supplement: S2 Fig — (TIFF) [file pone.0130497.s002.tiff]
