# Supplementary material for: Genome Scan for Selection in Structured Layer Chicken Populations Exploiting Linkage Disequilibrium Information
Source: PLoS One. 2015 Jul 7;10(7):e0130497. doi: 10.1371/journal.pone.0130497 (PMC4494984; doi:10.1371/journal.pone.0130497)
Supplement: S1 Table — All, White and Brown stands for studies with all layers, within white layers and within brown layers, respectively. (PDF) [file pone.0130497.s003.pdf]

Supplementary Table 1. Regions detected as putative selective sweeps by FLK method with upper 0.05% threshold. All, White and Brown stands for studies with all layers, within white layers and within brown layers, respectively.

Chr	Start	End	FLK	Test
1	876,801	879,393	18.18	All, White
1	1,447,884	1,918,112	18.18	All, white
1	38,501,132	38,743,192	15.29	Brown
1	72,000,056	72,000,056	18.03	All, White
1	81,514,762	81,617,589	18.99	All, White
1	85,564,990	85,564,990	22.31	All, White
1	89,714,612	89,958,818	15.29	Brown
1	94,278,058	94,350,858	19.92	All, White
1	110,893,174	110,901,608	14.19	Brown
1	122,878,602	123,575,939	18.03	All, White
2	20,823,662	20,823,662	21.33	All, White
2	30,599,514	30,756,158	19.22	All, White
2	37,811,029	37,811,029	18.03	All, White
2	77,671,653	77,829,435	14.36	Brown
2	134,444,108	134,511,569	21.66	All, White
3	9,103,949	9,464,866	18.18	All, White
3	10,433,119	10,433,119	17.99	All, White
3	12,736,662	12,839,916	18.18	All, White
3	16,195,426	16,501,848	18.18	All, White
3	29,252,847	29,252,847	14.36	Brown
3	74,769,008	74,769,008	18.22	All, White
3	76,027,460	76,756,006	18.35	All, White
4	1,946,359	1,946,359	17.37	All
4	4,132,955	4,606,317	27.01	All, White
4	38,537,874	38,537,874	14.36	Brown
4	48,911,210	48,911,210	14.76	Brown
4	57,462,535	57,736,499	21.67	All, White
4	82,428,770	82,642,951	17.74	All, White
4	89,562,515	89,645,321	26.79	All, White
5	9,560,855	9,727,104	19.60	All, White
5	10,483,424	10,869,666	17.37	All
5	30,977,615	30,986,726	14.76	Brown
5	32,973,636	33,689,397	18.35	All
5	33,689,397	33,689,397	18.02	white
5	34,192,489	34,192,489	18.18	All, White
5	44,980,131	45,324,225	18.22	All, White
6	3,857,340	4,542,453	25.21	All, White
6	14,494,277	15,513,209	26.81	All, White

6	16,239,040	16,239,040	19.84	All, White
6	24,742,685	25,333,094	27.01	All, White
6	30,071,833	30,555,513	19.60	All, White
6	31,806,976	31,867,965	18.35	All, White
7	4,573,380	4,573,380	19.92	All, White
7	11,712,633	12,018,314	21.49	All, White
7	15,401,874	15,554,717	18.74	All, White
8	7,772,316	7,796,487	19.92	All, White
9	15,158,147	15,541,760	23.24	All, White
9	16,767,582	16,767,582	17.88	All, White
9	20,484,006	20,491,673	17.99	All, White
10	6,717,862	6,829,903	23.24	All, White
10	9,787,437	9,795,191	19.38	All, White
10	13,240,503	13,313,302	23.43	All, White
10	16,903,725	17,024,113	18.03	All, White
10	18,687,536	18,692,572	18.27	All, White
11	1,129,121	1,273,109	21.49	All, White
11	5,850,574	5,852,027	16.23	Brown
11	7,828,349	7,846,020	17.99	All, White
12	2,504,479	2,504,479	17.20	All
13	481,800	481,800	16.23	Brown
13	2,198,792	2,227,737	30.64	All
13	2,198,792	2,198,792	30.29	white
14	5,312,749	5,339,593	19.72	All, White
14	9,923,476	9,923,476	23.08	All, White
17	4,819,521	4,819,521	17.74	All, White
18	2,316,233	2,316,233	18.03	All, White
18	9,945,284	9,945,284	15.29	Brown
19	8,078,819	8,148,720	19.92	All, White
21	3,298,371	3,657,433	19.92	All, White
21	3,954,063	4,186,034	16.23	Brown
22	1,500,456	1,500,456	19.72	All, White
23	4,166,191	4,166,191	14.44	Brown
23	4,697,093	4,697,093	18.03	All, White
24	2,693,250	2,693,250	19.95	All, White
24	4,739,168	4,791,387	23.08	All, White
26	2,746,599	2,746,599	17.34	All, White
27	2,376,218	2,404,136	14.19	Brown
27	3,464,176	3,464,176	14.76	Brown
28	469,149	469,181	17.88	All, White
28	1,133,950	1,143,821	17.32	All
28	3,507,916	3,760,475	19.72	All, White
28	3,573,243	3,680,447	14.36	Brown
28	4,385,539	4,504,460	15.29	Brown
