# Supplementary material for: Genome Scan for Selection in Structured Layer Chicken Populations Exploiting Linkage Disequilibrium Information
Source: PLoS One. 2015 Jul 7;10(7):e0130497. doi: 10.1371/journal.pone.0130497 (PMC4494984; doi:10.1371/journal.pone.0130497)
Supplement: S2 Table — (PDF) [file pone.0130497.s004.pdf]

Supplementary Table 2. List of genes for selective sweeps detected with FLK with 0.05% threshold in all layers.

Chr	Start	End	Description	FLK
1	621303	630454	interferon regulatory factor 5	18.18
1	630567	647745	transportin 3	18.18
1	658443	664773	Smoothened homolog	18.18
1	690901	708802	Adenosylhomocysteinase	18.18
1	710117	727850	striatin interacting protein 2	18.18
1	757267	795092	nuclear respiratory factor 1	18.18
1	807182	849202	ubiquitin-conjugating enzyme E2 H	18.18
1	850460	859444	nuclear-interacting partner of ALK	18.18
1	911097	915397	carboxypeptidase A5 precursor	18.18
1	916281	920045	carboxypeptidase A1 preproprotein	18.18
1	921538	929838	Centrosomal protein of 41 kDa	18.18
1	935413	954122	Coatomer subunit gamma	18.18
1	995787	1011804	ankyrin repeat domain-containing protein 16	18.18
1	1012026	1028924	rab GDP dissociation inhibitor beta	18.18
1	1016281	1061797	family with sequence similarity 208, member B	18.18
1	1068473	1076747	ankyrin repeat and SOCS box protein 13	18.18
1	1088060	1099472	neuroepithelial cell-transforming gene 1 protein	18.18
1	1361642	1822074	exocyst complex component 4	18.03
1	71808627	71817151	BCL2-like 14 (apoptosis facilitator)	18.03
1	71833436	71943987	low density lipoprotein receptor-related protein 6	18.03
1	71974273	71980456	MANSC domain containing 1 precursor	18.03
1	71983303	72042978	Loss of heterozygosity 12 chromosomal region 1 protein homolog	18.03
1	72046586	72113670	dual specificity phosphatase 16	18.03
1	72156810	72166175	G protein-coupled receptor 19	18.03
1	81373015	81656000	Limbic system-associated membrane protein	18.99
1	81680485	81734254	growth associated protein 43	18.99
1	94474405	94635350	glucan (1,4-alpha-), branching enzyme 1	18.03
1	122962850	123267815	FERM and PDZ domain containing 4	18.03
1	123394258	123418544	male-specific lethal 3 homolog (Drosophila)	18.03
2	20547214	20632812	multidrug resistance protein 1	21.33
2	20656627	20694939	RUN domain-containing protein 3B	21.33
2	20696196	20709565	solute carrier family 25, member 40	21.33
2	20711043	20726377	DBF4 homolog (S. cerevisiae)	21.33
2	20865806	20874342	sorcin	21.33
2	30537280	30564128	Sp4 transcription factor	19.22
2	30690282	30698829	cell division cycle-associated 7-like protein	19.22
2	30757166	30909032	Rap guanine nucleotide exchange factor (GEF) 5	19.22
2	30683539	30683661	U5 spliceosomal RNA	19.22
2	37711497	37756128	ubiquitin-conjugating enzyme E2E 1	18.03

2	37769194	37773772	Ribosomal protein L15	18.03
2	37787391	37809859	nuclear receptor subfamily 1 group D member 2	18.03
2	37844468	37965174	Thyroid hormone receptor beta	18.03
2	37698436	37698610	U1 spliceosomal RNA	18.03
2	134048833	134252747	trichorhinophalangeal syndrome I	21.66
2	134628301	134709262	Eukaryotic translation initiation factor 3 subunit H	21.66
3	9262357	9405997	WD repeat containing planar cell polarity effector	18.18
3	9406399	9417141	Malate dehydrogenase, cytoplasmic	18.18
3	9438436	9460691	UTP--glucose-1-phosphate uridylyltransferase	18.18
3	9462114	9489472	vacuolar protein sorting 54 homolog (S. cerevisiae)	18.18
3	9521292	9539167	protein pellino homolog 1	17.99
3	10359052	10449924	Meis homeobox 1	17.99
3	12844082	12878172	Delta-like protein	18.18
3	12887313	12960238	UPF0492 protein C20orf94 homolog	18.18
3	12961577	12970854	McKusick-Kaufman/Bardet-Biedl syndromes putative chaperonin	18.18
3	12995964	13027937	Synaptosomal-associated protein 25	18.18
3	16172663	16238767	chromogranin B (secretogranin 1)	18.18
3	16255281	16265499	DNA helicase MCM8	18.18
3	16271778	16276722	cardiolipin synthase 1	18.18
3	16360689	16391192	poly	18.18
3	16404585	16432600	protein lin-9 homolog	18.18
3	16453958	16471806	Golgi resident protein GCP60	18.18
3	16482887	16487420	histone H3.2	18.18
3	16515941	16518570	left-right determination factor 2 precursor	18.18
3	16598307	16603854	signal recognition particle 9 kDa protein	18.22
3	74490287	74540828	mitogen-activated protein kinase kinase kinase 7	18.22
3	74768582	74811042	BTB and CNC homology 1, basic leucine zipper transcription factor 2	18.22
3	74853141	74946896	Midasin	18.22
3	74953170	74985332	ankyrin repeat domain 6	18.22
3	74823486	74824862	gap junction protein, alpha 10, 62kDa	18.22
3	76289510	76312354	synaptotagmin binding, cytoplasmic RNA interacting protein	18.35
3	76326433	76373661	sorting nexin-14	18.35
3	76271065	76271131	Small nucleolar RNA SNORD50	18.35
3	76273207	76273277	Small nucleolar RNA SNORD50	18.35
4	1703060	1742346	Midline 2; Uncharacterized protein	17.37
4	1770827	1772689	TSC22 domain family, member 3	17.37
4	1790749	1795411	fatty acid amide hydrolase 2	17.37
4	1801657	1806508	chloride intracellular channel protein 2	17.37
4	1813109	1815939	G2/mitotic-specific cyclin-B3	17.37
4	1830442	1832824	shroom family member 4	17.37
4	1838457	1841000	bone morphogenetic protein 15 precursor	17.37
4	1858232	1866719	Histone deacetylase	17.37
4	1867724	1871061	40S ribosomal protein S4	17.37

4	1871906	1874674	RNA binding motif protein 41	17.37
4	1927710	1944396	FERM and PDZ domain containing 3	17.37
4	1950342	1960665	Ribose-phosphate pyrophosphokinase	17.37
4	1977381	1981476	myelin proteolipid protein	17.37
4	2006952	2017876	tyrosine-protein kinase BTK	17.37
4	2030171	2032347	aryl hydrocarbon receptor interacting protein-like 1	17.37
4	2035986	2044107	dystrophin related protein 2	17.37
4	2058474	2072715	Centromere protein I	17.37
4	2094842	2104015	H/ACA ribonucleoprotein complex subunit 4	17.37
4	2104399	2118953	55 kDa erythrocyte membrane protein	17.37
4	2122995	2142344	coagulation factor VIII, procoagulant component	17.37
4	2148375	2154417	FUN14 domain containing 2	17.37
4	2156282	2160713	C-x(9)-C motif containing 4 homolog (S. cerevisiae)	17.37
4	2163986	2168244	BRCA1/BRCA2-containing complex, subunit 3	17.37
4	2177525	2195453	Putative uncharacterized protein	17.37
4	2100634	2100709	Small nucleolar RNA SNORD83	17.37
4	2098331	2098464	Small nucleolar RNA SNORA36 family	17.37
4	2097884	2097959	Small nucleolar RNA SNORD83	17.37
4	2099885	2100131	Small nucleolar RNA SNORD17	17.37
4	2101395	2101544	Small nucleolar RNA SNORA56	17.37
4	2097097	2097343	Small nucleolar RNA SNORD17	17.37
4	4161930	4176418	solute carrier family 9, subfamily A (NHE6, cation proton antiporter 6), member 6	27.01
4	4206227	4209911	four and a half LIM domains 1	27.01
4	4214210	4248185	MAP7 domain containing 3	27.01
4	4280658	4292346	G protein-coupled receptor 112	27.01
4	4301225	4305523	bombesin receptor subtype-3	27.01
4	4345180	4349228	CD40 ligand CD40 ligand, membrane form CD40 ligand, soluble form	27.01
4	4354214	4383568	Rho guanine nucleotide exchange factor 6	27.01
4	4390057	4400409	heterogeneous nuclear ribonucleoprotein G	27.01
4	4398822	4398886	Small nucleolar RNA SNORD61	27.01
4	57404743	57409306	pituitary homeobox 2	21.67
4	57434843	57469617	glutamyl aminopeptidase (aminopeptidase A)	21.67
4	57576632	57597173	Elongation of very long chain fatty acids protein 6	21.67
4	57631764	57683454	pro-epidermal growth factor precursor	21.67
4	57708911	57722892	visual pigment-like receptor peropsin	21.67
4	57731319	57744001	complement factor I	21.67
4	57745870	57751937	phospholipase A2, group XIIA	21.67
4	57754986	57763766	caspase-6	21.67
4	57807176	57923545	PDZ and LIM domain protein 5	17.74
4	82408395	82444435	max dimerization protein 4	17.74
4	82459166	82544586	polymerase (DNA directed) nu	17.74
4	82738354	82761647	negative elongation factor A	17.74
4	82783104	82842064	Wolf-Hirschhorn syndrome candidate 1	26.79

4	89402351	89584157	exocyst complex component 6B	26.79
5	9538613	9582720	LIM domain only 1 (rhombotin 1)	19.60
5	9614317	9626415	resistance to inhibitors of cholinesterase 3 homolog (C. elegans)	19.60
5	9634001	9761468	tubby homolog (mouse)	19.60
5	9768005	9802730	ras-related protein R-Ras2	19.60
5	9834069	9848544	Coatomer subunit beta	19.60
5	9851015	9858153	proteasome subunit alpha type-1	19.60
5	9876942	9951911	cGMP-inhibited 3,5-cyclic phosphodiesterase B	17.37
5	10549851	10784968	SRY (sex determining region Y)-box 6	17.37
5	33101816	33216460	serine/threonine-protein kinase D1	18.35
5	33413582	33433671	G2/M phase-specific E3 ubiquitin-protein ligase	18.35
5	33438355	33483021	sec1 family domain-containing protein 1	18.35
5	33517885	33534657	Cochlin	18.35
5	33543459	33598778	striatin, calmodulin binding protein 3	18.18
5	34050021	34304671	A kinase (PRKA) anchor protein 6	18.18
5	34375148	34959900	neuronal PAS domain protein 3	18.22
5	44981707	44983006	homeobox protein goosecoid	18.22
5	45092963	45128794	endoribonuclease Dicer	18.22
5	45165017	45184249	calmin (calponin-like, transmembrane)	18.22
5	45270524	45300174	spectrin repeat containing, nuclear envelope family member 3	18.22
5	45330738	45337464	glutaredoxin-related protein 5, mitochondrial	18.22
5	45328658	45328930	Small Cajal body specific RNA 13	18.22
6	3953643	3956579	lung lectin precursor	25.21
6	3961091	3967496	surfactant, pulmonary-associated protein A1 precursor	25.21
6	3981178	3989358	soluble mannose-binding lectin precursor	25.21
6	4283164	4363396	ret proto-oncogene precursor	25.21
6	14667844	14924979	adenosine kinase	26.81
6	14941845	14956153	AP-3 complex subunit mu-1	26.81
6	14961440	14990291	Vinculin	26.81
6	15060198	15068452	urokinase-type plasminogen activator preproprotein	26.81
6	16111040	16115551	zona pellucida sperm-binding protein 4	19.84
6	16273210	16314303	Phosphoinositide 3-kinase adapter protein 1	19.84
6	16318282	16329867	nucleolar and coiled-body phosphoprotein 1	19.84
6	16349917	16354551	ELOVL fatty acid elongase 3	19.84
6	16379333	16476177	golgi brefeldin A resistant guanine nucleotide exchange factor 1	19.84
6	16488268	16493350	nuclear factor NF-kappa-B p100 subunit	19.60
6	24664740	24940362	sortilin-related VPS10 domain containing receptor 1	19.60
6	25264735	25352280	gamma-adducin	19.60
6	30060586	30109050	phosphatidic acid phosphatase type 2 domain containing 1A	19.60
6	30185811	30223053	WD repeat domain 11	19.60
6	30411293	30490016	Fibroblast growth factor receptor 2	19.60
6	30550113	30621520	arginyl-tRNA--protein transferase 1	18.35
6	31636707	31661235	family with sequence similarity 53, member B	18.35

6	31686516	31702105	BRISC complex subunit Abro1	18.35
6	32007895	32012786	testis expressed 36	18.35
6	32058339	32075635	DEAH (Asp-Glu-Ala-His) box polypeptide 32	18.35
7	4312646	4330369	bifunctional purine biosynthesis protein PURH	19.92
7	4333468	4382817	fibronectin precursor	19.92
7	4527554	4590262	NEDD8-conjugating enzyme UBE2F	19.92
7	4591385	4595864	receptor (G protein-coupled) activity modifying protein 1	19.92
7	4611641	4662295	leucine rich repeat (in FLII) interacting protein 1	19.92
7	4722132	4740447	melanophilin	19.92
7	4752831	4805577	collagen alpha-3(VI) chain precursor	19.92
7	11536104	11616253	pleckstrin homology domain containing, family M, member 3	21.49
7	11686741	11704137	cyclic AMP-responsive element-binding protein 1	21.49
7	11766883	11819797	Kruppel-like factor 7 (ubiquitous)	21.49
7	11854607	11868058	carboxypeptidase O	21.49
7	11950838	12015748	disintegrin and metalloproteinase domain-containing protein 23	21.49
7	12072509	12076665	elongation factor 1-beta	21.49
7	12077169	12090936	NADH-ubiquinone oxidoreductase 75 kDa subunit, mitochondrial	21.49
7	12107101	12129187	INO80 complex subunit D	21.49
7	12074025	12074158	Small nucleolar RNA SNORA41	21.49
7	12074705	12074740	Small nucleolar RNA Z196/R39/R59 family	21.49
7	15219971	15271809	alkylglycerone phosphate synthase	18.74
7	15304546	15320356	nuclear factor erythroid 2-related factor 2	18.74
8	7564281	7592106	smg-7 homolog, nonsense mediated mRNA decay factor (C. elegans)	19.92
8	7604691	7609670	actin-related protein 2/3 complex subunit 5	19.92
8	7641619	7708062	ral guanine nucleotide dissociation stimulator-like 1	19.92
8	7903724	7931488	ER degradation enhancer, mannosidase alpha-like 3	19.92
8	7933908	7992849	protein Niban	19.92
8	7564912	7565074	U1 spliceosomal RNA	19.92
9	15087461	15106244	eukaryotic translation initiation factor 4E family member 2	23.24
9	15246615	15264143	eukaryotic translation initiation factor 2B, subunit 5 epsilon, 82kDa	23.24
9	15281328	15292012	dishevelled, dsh homolog 3 (Drosophila)	23.24
9	15293532	15316234	AP-2 complex subunit mu	23.24
9	15332834	15340129	von Willebrand factor A domain containing 5B2	23.24
9	15340601	15343371	ALG3, alpha-1,3- mannosyltransferase	23.24
9	15356178	15362770	endothelin converting enzyme 2	23.24
9	15363725	15369726	26S proteasome non-ATPase regulatory subunit 2	23.24
9	15371232	15387501	eukaryotic translation initiation factor 4 gamma, 1	23.24
9	15395572	15405268	chloride channel, voltage-sensitive 2	23.24
9	15406844	15413438	chordin precursor	23.24
9	15419502	15421617	thrombopoietin precursor	23.24
9	15437757	15449465	EPH receptor B3	23.24
9	15380309	15380385	Small nucleolar RNA SNORD66	23.24
9	15381519	15381596	Small nucleolar RNA SNORD66	23.24

9	16565596	16587667	fragile X mental retardation syndrome-related protein 1	17.88
9	16663283	16672397	tetratricopeptide repeat protein 14	17.88
9	16760382	16850020	peroxisomal biogenesis factor 5-like	17.88
9	16861502	16902461	Ubiquitin carboxyl-terminal hydrolase 13	17.88
9	16903953	16907474	NADH dehydrogenase	17.88
9	16911951	16921482	actin-like 6A	17.88
9	16984938	17003984	mitofusin-1	17.88
9	16820695	16820798	U6 spliceosomal RNA	17.88
9	20419761	20464371	cholinesterase precursor	17.99
9	20586079	20587887	SLIT and NTRK-like family, member 3	17.99
10	6434758	6619897	disintegrin and metalloproteinase domain-containing protein 10 precursor	23.24
10	6572777	6596733	aquaporin 9	23.24
10	6604818	6661741	Retinal dehydrogenase 2	23.24
10	6816944	6857906	cingulin-like 1	23.24
10	6903270	7058903	transcription factor 12	19.38
10	9545024	9553009	solute carrier family 24, member 5 precursor	19.38
10	9551752	9570906	myelin expression factor 2	19.38
10	9587065	9632792	solute carrier family 12 (sodium/potassium/chloride transporters), member 1	19.38
10	17122370	17146830	proprotein convertase subtilisin/kexin type 6	18.03
10	17199826	17220109	ankyrin repeat and death domain containing 1A	18.27
10	18440729	18553284	dual specificity mitogen-activated protein kinase kinase 5	18.27
10	18595984	18624673	E3 SUMO-protein ligase PIAS1	18.27
10	18632517	18638897	ceroid-lipofuscinosis, neuronal 6, late infantile, variant	18.27
10	18641302	18648029	protein fem-1 homolog B	18.27
10	18699925	18729841	coronin, actin binding protein, 2B	18.27
10	18770495	18779187	NADPH oxidase 5	18.27
10	18807760	18817356	glucuronic acid epimerase	18.27
10	18823404	18826891	progestin and adipoQ receptor family member V	18.27
10	18828578	18846365	kinesin-like protein KIF23	18.27
11	983068	995051	E2F transcription factor 4; Uncharacterized protein	21.49
11	995632	1005522	engulfment and cell motility 3	21.49
11	1011955	1025358	KIAA0895-like	21.49
11	1029318	1036713	lecithin-cholesterol acyltransferase	21.49
11	1073882	1097277	family with sequence similarity 65, member A	21.49
11	1102641	1128402	transcriptional repressor CTCF	21.49
11	1130092	1147360	RGD motif, leucine rich repeats, tropomodulin domain and proline-rich containing	21.49
11	1165618	1177771	alanyl-tRNA synthetase, cytoplasmic	21.49
11	1189504	1194020	fibulin 7	21.49
11	1213866	1215689	tubulin polymerization-promoting protein family member 3	21.49
11	1223848	1237337	potassium channel tetramerisation domain containing 19	21.49
11	1240508	1302916	pleckstrin homology domain containing, family G (with RhoGef domain) member 4	21.49
11	1336785	1351404	FH1/FH2 domain-containing protein 1	21.49

11	1389699	1391495	agouti-related protein precursor	21.49
11	1398993	1402340	N-lysine methyltransferase SETD6	21.49
11	1405351	1453089	CCR4-NOT transcription complex, subunit 1	21.49
11	1424514	1424649	Small nucleolar RNA SNORA46	21.49
11	1432730	1432863	Small nucleolar RNA SNORA76	21.49
11	7596758	7603866	Cytochrome b-c1 complex subunit Rieske, mitochondrial	17.99
11	7841312	7844172	processing of precursor 4	17.99
11	7933476	7946064	G1/S-specific cyclin-E1	17.99
11	7961122	8028603	URI1, prefoldin-like chaperone	17.99
12	2286271	2288801	guanylate binding protein	17.20
12	2342130	2383187	ring finger protein 123	17.20
12	2383108	2385611	GDP-mannose pyrophosphorylase B	17.20
12	2420987	2427304	cadherin-related family member 4	17.20
12	2427451	2434824	ubiquitin-like modifier activating enzyme 7	17.20
12	2435916	2437477	aminomethyltransferase, mitochondrial precursor	17.20
12	2441415	2452604	dystroglycan precursor	17.20
12	2479299	2483435	hepatocyte growth factor-like protein precursor	17.20
12	2487790	2491678	acylamino-acid-releasing enzyme	17.20
12	2494176	2539876	bassoon presynaptic cytomatrix protein	17.20
12	2549854	2567887	TRAF-interacting protein	17.20
12	2568476	2571564	CaM kinase-like vesicle-associated	17.20
12	2579806	2587122	macrophage-stimulating protein receptor precursor	17.20
12	2588483	2592570	MON1 homolog A (yeast)	17.20
12	2598043	2648508	RNA-binding protein 6	17.20
12	2659272	2697071	RAD54-like 2 (S. cerevisiae)	17.20
12	2749644	2795258	Fanconi anemia, complementation group D2	17.20
12	2379402	2380922	adhesion molecule with Ig-like domain 3	17.20
13	2010626	2012530	leucine rich repeat transmembrane neuronal 2	30.64
13	2134785	2156387	stress-70 protein, mitochondrial precursor	30.64
13	2146299	2146368	Small nucleolar RNA SNORD63	30.64
14	5075181	5086183	DNA topoisomerase 3-alpha	19.72
14	5112713	5127320	phosphoribosyl pyrophosphate synthase-associated protein 2	19.72
14	5129153	5157900	solute carrier family 5 (sodium/glucose cotransporter), member 10	19.72
14	5138381	5150303	family with sequence similarity 83, member G	19.72
14	5223282	5225616	B9 protein domain 1	19.72
14	5230050	5338554	Voltage-dependent T-type calcium channel subunit alpha-1H	19.72
14	9934515	9974931	ubiquitin carboxyl-terminal hydrolase 7	23.08
14	10046044	10052775	phosphomannomutase 2	23.08
14	10103934	10114456	methyltransferase-like protein 22	23.08
17	4788861	4790491	ovoglycoprotein precursor	17.74
17	4820725	4824113	nuclear apoptosis inducing factor 1	17.74
17	4829664	4860591	early estrogen-induced gene 1 protein	17.74
17	4865441	4867919	dolichol phosphate-mannose biosynthesis regulatory protein	17.74

17	4870177	4871635	alpha-N-acetyl-neuraminyl-2,3-beta-galactosyl-1	17.74
17	4872095	4874530	ST6 -N-acetylgalactosaminide alpha-2,6-sialyltransferase 6	17.74
17	4877410	4880796	Adenylate kinase isoenzyme 1	17.74
17	4882932	4891674	endoglin precursor	17.74
17	4892549	4896134	folylpolyglutamate synthase	17.74
17	4896492	4902733	Cyclin-dependent kinase 9	17.74
17	4903795	4923543	SH2 domain containing 3C	17.74
17	4929079	4931968	tetratricopeptide repeat domain 16	17.74
17	4933496	4946785	cerebral endothelial cell adhesion molecule	17.74
17	4949346	4967354	Ubiquitin-related modifier 1 homolog	17.74
17	4983785	4987536	coenzyme Q4 homolog (S. cerevisiae)	17.74
17	5016759	5075578	dynamin 1	17.74
18	2020996	2066954	Netrin-1	18.03
18	2070106	2146328	syntaxin-8	18.03
18	2180734	2183708	ubiquitin specific peptidase 43	18.03
18	2188691	2205886	Rho GTPase activating protein 44	18.03
18	2223389	2230202	uncharacterized protein LOC417324	18.03
19	7951502	7972199	protein phosphatase, Mg2+/Mn2+ dependent, 1D	19.92
19	7979200	7997166	amyloid protein-binding protein 2	19.92
19	8096015	8104574	carbonic anhydrase IV	19.92
19	8148378	8165006	Gametogenetin-binding protein 2	19.92
19	8166412	8187734	Dehydrogenase/reductase SDR family member 11	19.92
21	3252441	3261217	G protein-coupled receptor 157	19.92
21	3470173	3492933	phosphatidylinositol-4,5-bisphosphate 3-kinase catalytic subunit delta isoform	19.92
21	3493075	3527740	calsyntenin-1 precursor	19.92
21	3573056	3581801	Protein LZIC	19.92
21	3581913	3588648	nicotinamide nucleotide adenylyltransferase 1	19.92
21	3590985	3592942	retinol binding protein 7, cellular	19.92
21	3595756	3630094	ubiquitination factor E4B	19.92
21	3639974	3713856	kinesin family member 1B	19.92
21	3719439	3729173	6-phosphogluconate dehydrogenase, decarboxylating	19.92
21	3705900	3706069	TUC338	19.92
22	1256816	1265197	Charged multivesicular body protein 7	19.72
22	1270094	1276295	tumor necrosis factor receptor superfamily member 10B precursor	19.72
22	1343627	1375329	exportin-7	19.72
22	1376680	1381157	docking protein 2, 56kDa	19.72
22	1397665	1418597	GDNF family receptor alpha-2 precursor	19.72
22	1597651	1602693	TELO2 interacting protein 2	19.72
23	4570631	4804042	CUB and Sushi multiple domains 2	18.03
23	4824370	4834663	collagen, type IX, alpha 2	18.03
23	4835192	4847417	small ArfGAP2	18.03
23	4901524	4907272	potassium voltage-gated channel, KQT-like subfamily, member 4	18.03
23	4910836	4916448	tubulointerstitial nephritis antigen-like 1	18.03

23	4918792	4920966	penta-EF-hand domain containing 1	18.03
23	4921631	4939423	collagen, type XVI, alpha 1	18.03
23	4943828	4960852	brain-specific angiogenesis inhibitor 2	18.03
24	2427011	2464846	immunoglobulin superfamily, member 9B	19.95
24	2516848	2542717	non-SMC condensin II complex, subunit D3	19.95
24	2553386	2558481	Thymocyte nuclear protein 1	19.95
24	2845500	2863118	potassium-transporting ATPase alpha chain 2	19.95
24	2902288	3002638	GRAM domain containing 1B	23.08
24	4392700	4566947	ubiquitin conjugation factor E4 A	23.08
24	4557911	4561306	RNA-binding protein 7	23.08
24	4664469	4732605	cell adhesion molecule 1	23.08
26	2504099	2506388	Interleukin-10	17.34
26	2517673	2519712	interleukin 19	17.34
26	2520032	2531914	polymeric immunoglobulin receptor precursor	17.34
26	2556703	2560172	ubiquitin thioesterase OTU1	17.34
26	2576519	2583374	C4b-binding protein alpha chain precursor	17.34
26	2599568	2605277	complement component (3b/4b) receptor 1-like precursor	17.34
26	2627129	2639856	complement component 4 binding protein, alpha chain precursor	17.34
28	271548	395862	Fibrillin-3; Uncharacterized protein	17.88
28	509129	534490	heterogeneous nuclear ribonucleoprotein M	17.88
28	558963	564318	U6 snRNA-associated Sm-like protein LSm7	17.88
28	564384	599425	signal peptide peptidase-like 2B precursor	17.88
28	627972	628532	translocase of inner mitochondrial membrane 13 homolog (yeast)	17.88
28	636058	661825	lamin-B2	17.88
28	676818	677614	60S ribosomal protein L36	17.88
28	678763	694768	lon peptidase 1, mitochondrial	17.88
28	701484	724473	solute carrier family 1 (high affinity aspartate/glutamate transporter), member 6	17.32
28	899423	901661	anti-dorsalizing morphogenetic protein precursor	17.32
28	932572	950125	ELAV-like protein 1	17.32
28	961655	965629	megakaryocyte-associated tyrosine kinase	17.32
28	972503	976394	Retinal homeobox protein Rx1	17.32
28	976369	983104	mucosa-associated lymphoid tissue lymphoma translocation protein 1-like	17.32
28	991289	1002123	tight junction protein 3	17.32
28	1036061	1041471	cactin, spliceosome C complex subunit	17.32
28	1041856	1045661	thromboxane A2 receptor	17.32
28	1047302	1049378	GIPC PDZ domain containing family, member 3	17.32
28	1049765	1054525	high mobility group 20B	17.32
28	1086890	1089949	Deoxyhypusine hydroxylase	17.32
28	1102700	1127154	unc-13 homolog A (C. elegans)	17.32
28	1143774	1145084	phosphatidic acid phosphatase type 2C	17.32
28	1171971	1179236	amino-terminal enhancer of split	17.32
28	1195931	1202586	guanine nucleotide-binding protein subunit alpha-11	17.32
28	1218202	1225645	nicalin precursor	17.32

28	1233711	1264738	CUGBP, Elav-like family member 5	17.32
28	1266179	1271107	Hydroxysteroid 11-beta-dehydrogenase 1-like protein	17.32
28	1303285	1407097	MPN domain containing	19.72
28	3410986	3425610	solute carrier family 25, member 42	19.72
28	3470619	3483167	homer homolog 3 (Drosophila)	19.72
28	3489321	3495011	probable ATP-dependent RNA helicase DDX49	19.72
28	3495159	3499962	Coatomer subunit epsilon	19.72
28	3514060	3516165	growth differentiation factor 3	19.72
28	3517866	3537861	UPF1 regulator of nonsense transcripts homolog (yeast)	19.72
28	3651445	3653912	cytokine receptor-like factor 1	19.72
28	3657472	3659967	KxDL motif containing 1	19.72
28	3666228	3700318	RNA polymerase II elongation factor ELL	19.72
28	3728960	3735148	LSM4 homolog, U6 small nuclear RNA associated (S. cerevisiae)	19.72
28	3748373	3753211	phosphodiesterase 4C, cAMP-specific	19.72
28	3758472	3759385	MPV17 mitochondrial membrane protein-like 2	19.72
28	3759501	3762347	Interferon-gamma-inducible lysosomal thiol reductase	19.72
28	3782857	3800231	microtubule associated serine/threonine kinase 3	19.72
28	3800995	3805633	interleukin 12 receptor, beta 1	19.72
28	3810197	3816386	arrestin domain containing 2	19.72
28	3820870	3825356	peroxisomal membrane protein 11C	19.72
28	3878507	3912897	Tyrosine-protein kinase receptor	19.72
