# Supplementary material for: Genome Scan for Selection in Structured Layer Chicken Populations Exploiting Linkage Disequilibrium Information
Source: PLoS One. 2015 Jul 7;10(7):e0130497. doi: 10.1371/journal.pone.0130497 (PMC4494984; doi:10.1371/journal.pone.0130497)
Supplement: S4 Table — (PDF) [file pone.0130497.s006.pdf]

Supplementary Table 4. List of genes for selective sweeps detected with FLK with 0.05% threshold in brown layers.

Chr	Start	End	Description	FLK
1	38593310	38850635	neuron navigator 3	15.29
1	110613430	110658911	monoamine oxidase A	15.29
2	77784558	78033401	catenin (cadherin-associated protein), delta 2	14.36
3	29337956	29424515	MAM domain-containing glycosylphosphatidylinositol anchor protein 1 precursor	14.36
3	29217044	29217179	TUC338	14.36
4	38695965	38707538	ufm1-specific protease 2	14.36
4	38707911	38715236	LRP2 binding protein	14.36
4	38717204	38769907	sorting nexin 25	14.36
4	38786566	38804359	KIAA1430	14.36
4	48659743	48674369	signal recognition particle 72 kDa protein	14.36
4	48676608	48680544	ADP-ribosylation factor-like 9	14.76
4	48685349	48688644	Homeodomain-only protein	14.76
4	48706786	48712370	serine peptidase inhibitor, Kazal type 2 (acrosin-trypsin inhibitor)	14.76
4	48787273	48788448	dual specificity protein phosphatase 4	14.76
4	48794273	48900800	tankyrase-1	14.76
4	48927640	48936506	3-5 exoribonuclease 1	14.76
4	48960039	48966650	Protein phosphatase 1 regulatory subunit 3	14.76
4	49033103	49034980	ghrelin O-acyltransferase	14.76
4	49041665	49042706	Ribonuclease CL2	14.76
4	49077854	49125067	septin 11	14.76
4	49129165	49130967	sosondowah ankyrin repeat domain family member B	14.76
4	49142913	49148727	16 kDa beta-galactoside-binding lectin	14.76
4	49156409	49291621	shroom family member 3	14.76
5	30717442	30832671	uncharacterized protein C15orf41 homolog	16.23
11	5636333	5654522	cylindromatosis (turban tumor syndrome)	16.23
11	5725577	5829415	naked cuticle homolog 1 (Drosophila)	16.23
11	5990712	6006669	bromodomain-containing protein 7	16.23
11	6010767	6037599	adenylate cyclase 7	16.23
13	185486	285392	protocadherin alpha 11 precursor	16.23
13	365453	377641	histidyl-tRNA synthetase, cytoplasmic	16.23
13	384539	392849	protein Red	16.23
13	392987	394389	NADH dehydrogenase	16.23
13	418871	424514	Transmembrane protein 173	16.23
13	402430	403827	CD14 molecule precursor	16.23
13	380747	380840	Vault RNA	16.23
18	9623865	9743508	regulatory associated protein of MTOR, complex 1	15.29
18	9754645	9756687	neuronal pentraxin I	15.29
18	9763404	9771339	endonuclease V	15.29
18	9771940	9818172	ring finger protein 213	15.29

18	9819301	9824829	solute carrier family 26, member 11	15.29
18	9885036	9886813	E3 SUMO-protein ligase CBX4	15.29
18	10016690	10022596	RNA binding protein, fox-1 homolog (C. elegans) 3	15.29
18	10024650	10029994	endo-beta-N-acetylglucosaminidase	15.29
18	10034762	10038434	soluble calcium-activated nucleotidase 1	15.29
18	10040657	10047611	metalloproteinase inhibitor 2 precursor	15.29
18	10062346	10070304	cytohesin-1	15.29
18	10070951	10098653	dynein, axonemal, heavy chain 17	15.29
18	10096967	10115046	CDP-diacylglycerol--glycerol-3-phosphate 3-phosphatidyltransferase, mitochondrial	15.29
18	10142947	10144362	Thymidine kinase, cytosolic	15.29
18	10145914	10147671	synaptogyrin 2	15.29
18	10154424	10156574	ADP-ribosylation factor-like 16	15.29
18	10181308	10184603	gastric inhibitory polypeptide receptor precursor	15.29
18	10183770	10202043	protein disulfide-isomerase precursor	15.29
18	10116718	10117433	suppressor of cytokine signaling 3	15.29
21	3826146	3869802	castor zinc finger 1	16.23
21	4065712	4071374	TAR DNA-binding protein 43	16.23
21	4073317	4086699	mannan-binding lectin serine protease 2 precursor	16.23
21	4099857	4103582	Pro2-somatostatin precursor	16.23
21	4118177	4132290	exosome component 10	16.23
21	4133269	4193503	mechanistic target of rapamycin (serine/threonine kinase)	16.23
21	4195215	4200725	ubiA prenyltransferase domain-containing protein 1	16.23
21	4202008	4221119	pleckstrin homology domain containing, family M (with RUN domain) member 2	16.23
21	4229458	4231641	filamin binding LIM protein 1	16.23
21	4260524	4301058	spen homolog, transcriptional regulator (Drosophila)	16.23
21	4191831	4192009	TUC338	16.23
23	3965824	3970617	granulocyte colony-stimulating factor receptor	14.44
23	3977706	3987124	organic solute carrier partner 1	14.44
23	3988933	4004007	serine/threonine-protein kinase 40	14.44
23	4007214	4007707	eva-1 homolog B (C. elegans)	14.44
23	4008688	4024899	thyroid hormone receptor-associated protein 3	14.44
23	4031627	4038165	MAP7 domain containing 1	14.44
23	4050995	4056202	Trafficking protein particle complex subunit 3	14.44
23	4092973	4097739	poly(ADP-ribose) glycohydrolase ARH3	14.44
23	4098117	4102886	tektin 2 (testicular)	14.44
23	4111971	4133770	protein argonaute-3	14.44
23	4176483	4190009	claspin	14.44
23	4222202	4234872	Proteasome subunit beta type	14.44
23	4256435	4263091	neurochondrin	14.44
23	4264511	4288627	KIAA0319-like	14.44
23	4295102	4296014	interferon alpha-inducible protein 27-like protein 2	14.44
23	4343978	4352202	Gizzard PTB-associated splicing factor; Uncharacterized protein	14.44
23	4361390	4361969	ZMYM6 neighbor	14.44

23	4379948	4393033	discs, large (Drosophila) homolog-associated protein 3	14.44
23	4398099	4400981	connexin 37	14.44
23	4404012	4404779	gap junction protein, beta 3, 31kDa	14.44
27	2226991	2253136	corticotropin-releasing factor receptor 1 precursor	14.19
27	2302640	2319626	integrin beta-3 precursor	14.19
27	2332100	2344617	Methyltransferase-like protein 2	14.19
27	2357455	2399073	tousled-like kinase 2	14.19
27	2412671	2426010	mannose receptor, C type 2	14.19
27	2524612	2629807	tetratricopeptide repeat, ankyrin repeat and coiled-coil containing 2	14.19
27	2634670	2639226	cytochrome b561	14.19
27	3204183	3219385	tumor necrosis factor receptor superfamily member 16 precursor	14.19
27	3252521	3267354	membrane protein, palmitoylated 3 (MAGUK p55 subfamily member 3)	14.76
27	3315353	3321406	homeobox protein MOX-1	14.76
27	3335561	3350183	ets variant 4	14.76
27	3352213	3364975	DEAH (Asp-Glu-Ala-His) box polypeptide 8	14.76
27	3366784	3370919	Prohibitin	14.76
27	3413412	3417057	phosphoethanolamine/phosphocholine phosphatase	14.76
27	3423443	3425719	guanine nucleotide binding protein (G protein), gamma transducing activity polypeptide 2	14.76
27	3433057	3459259	Insulin-like growth factor 2 mRNA-binding protein 1	14.76
27	3468107	3475761	gastric inhibitory polypeptide precursor	14.76
27	3495909	3506537	calcium binding and coiled-coil domain 2	14.76
27	3586132	3589844	Hoxb-7	14.76
27	3598354	3600970	homeobox B6	14.76
27	3604171	3606516	Homeobox protein Hox-B5	14.76
27	3621645	3626317	homeobox protein Hox-B4	14.76
27	3643412	3649970	homeobox protein Hox-B3	14.76
27	3652538	3655356	homeobox B2	14.76
27	3662963	3664600	homeobox B1	14.76
27	3711055	3815879	src kinase associated phosphoprotein 1	14.76
28	3410986	3425610	solute carrier family 25, member 42	14.36
28	3470619	3483167	homer homolog 3 (Drosophila)	14.36
28	3489321	3495011	probable ATP-dependent RNA helicase DDX49	14.36
28	3495159	3499962	Coatomer subunit epsilon	14.36
28	3514060	3516165	growth differentiation factor 3	14.36
28	3517866	3537861	UPF1 regulator of nonsense transcripts homolog (yeast)	14.36
28	3651445	3653912	cytokine receptor-like factor 1	14.36
28	3657472	3659967	KxDL motif containing 1	14.36
28	3666228	3700318	RNA polymerase II elongation factor ELL	14.36
28	3728960	3735148	LSM4 homolog, U6 small nuclear RNA associated (S. cerevisiae)	14.36
28	3748373	3753211	phosphodiesterase 4C, cAMP-specific	14.36
28	3758472	3759385	MPV17 mitochondrial membrane protein-like 2	14.36
28	3759501	3762347	Interferon-gamma-inducible lysosomal thiol reductase	14.36
28	3782857	3800231	microtubule associated serine/threonine kinase 3	14.36

28	3800995	3805633	interleukin 12 receptor, beta 1	14.36
28	3810197	3816386	arrestin domain containing 2	14.36
28	3820870	3825356	peroxisomal membrane protein 11C	14.36
28	4195685	4203208	tropomyosin 4	15.29
28	4410520	4497158	receptor-type tyrosine-protein phosphatase delta precursor	15.29
28	4512762	4588636	lysine (K)-specific demethylase 4B	15.29
28	4673752	4692777	dipeptidyl-peptidase 9	15.29
28	4625272	4627738	toll-like receptor adaptor molecule 1	15.29
