# Supplementary material for: Genome Scan for Selection in Structured Layer Chicken Populations Exploiting Linkage Disequilibrium Information
Source: PLoS One. 2015 Jul 7;10(7):e0130497. doi: 10.1371/journal.pone.0130497 (PMC4494984; doi:10.1371/journal.pone.0130497)
Supplement: S5 Table — (PDF) [file pone.0130497.s007.pdf]

Supplementary Table 5. Lists of pathways and gene ontologies under selection with FLK with 0.05% threshold in all layers.

Description	# Genes anotated	Genes of pathways (%)*	P-Value
membrane	22	1.1	0.000
integral to membrane	21	1.3	0.000
zinc ion binding	18	1.5	0.000
intracellular	18	1.5	0.000
plasma membrane	15	1.5	0.000
Metabolic pathways	17	12.5	0.000
ATP binding	19	1.8	0.000
DNA binding	16	1.7	0.000
nucleotide binding	17	1.8	0.000
mitochondrion	17	1.9	0.000
regulation of transcription, DNA-dependent	15	1.9	0.000
signal transduction	17	2.2	0.000
metal ion binding	14	2.0	0.001
COPI-coated vesicle membrane	3	60.0	0.001
maintenance of DNA methylation	3	60.0	0.001
nucleic acid binding	13	2.1	0.002
intercellular bridge	3	50.0	0.002
protein K63-linked deubiquitination	4	30.8	0.002
cellular response to oxidative stress	4	28.6	0.003
positive regulation of transcription from RNA polymerase II promoter	9	2.0	0.004
G-protein coupled receptor signaling pathway	8	1.9	0.004
COPI vesicle coat	3	37.5	0.005
exocyst	3	37.5	0.005
regulation of cell size	3	37.5	0.005
endoplasmic reticulum unfolded protein response	4	23.5	0.006
urogenital system development	3	30.0	0.009
sequence-specific DNA binding transcription factor activity	13	2.4	0.009
ubiquitin ligase complex	5	16.7	0.011
retinoic acid receptor signaling pathway	3	27.3	0.012
Neuroactive ligand-receptor interaction	5	12.5	0.012
protein phosphorylation	12	2.4	0.013
regulation of cyclin-dependent protein serine/threonine kinase activity	4	17.4	0.019
vesicle docking involved in exocytosis	3	23.1	0.019
cytosol	9	2.2	0.021
sequence-specific DNA binding	11	2.4	0.022
ubiquitin-specific protease activity	5	13.9	0.023
dorsal/ventral neural tube patterning	3	21.4	0.024
Golgi apparatus	10	2.4	0.024
transferase activity, transferring phosphorus-containing groups	12	2.5	0.026

positive regulation of proteolysis	3	20.0	0.029
endoplasmic reticulum	8	2.2	0.029
cell periphery	4	14.8	0.033
one-carbon metabolic process	3	18.8	0.034
ATP metabolic process	3	18.8	0.034
guanyl-nucleotide exchange factor activity	6	11.1	0.036
signal transducer activity	8	2.3	0.038
G-protein coupled receptor activity	8	2.3	0.038
negative regulation of intrinsic apoptotic signaling pathway	3	17.6	0.040
negative regulation of tumor necrosis factor production	3	17.6	0.040
One carbon pool by folate	3	12.5	0.040
Galactose metabolism	4	12.5	0.040
protein kinase activity	12	2.6	0.041
negative regulation of NF-kappaB transcription factor activity	4	13.8	0.042
transcription, DNA-dependent	6	2.1	0.044
extracellular region	11	2.6	0.046
hair follicle morphogenesis	3	16.7	0.047
positive regulation of fat cell differentiation	3	16.7	0.047
G2 DNA damage checkpoint	3	16.7	0.047
ubiquitin thiolesterase activity	6	10.3	0.049

*Percentage of the genes of the pathway which were among the annotated genes.
