# Supplementary material for: Genome Scan for Selection in Structured Layer Chicken Populations Exploiting Linkage Disequilibrium Information
Source: PLoS One. 2015 Jul 7;10(7):e0130497. doi: 10.1371/journal.pone.0130497 (PMC4494984; doi:10.1371/journal.pone.0130497)
Supplement: S6 Table — (PDF) [file pone.0130497.s008.pdf]

Supplementary Table 6. Lists of pathways and gene ontologies under selection with FLK with 0.05% threshold in white layers.

Description	# Genes anotated	Genes of pathways (%)*	P-Value
membrane	21	1.1	0.000
integral to membrane	20	1.3	0.000
zinc ion binding	17	1.4	0.000
Metabolic pathways	16	33.3	0.000
intracellular	18	1.5	0.000
plasma membrane	14	1.4	0.000
ATP binding	17	1.6	0.000
DNA binding	16	1.7	0.000
mitochondrion	16	1.8	0.000
nucleotide binding	17	1.8	0.000
COPI-coated vesicle membrane	3	60.0	0.001
maintenance of DNA methylation	3	60.0	0.001
intercellular bridge	3	50.0	0.001
metal ion binding	12	1.7	0.001
regulation of transcription, DNA-dependent	15	1.9	0.001
COPI vesicle coat	3	37.5	0.003
exocyst	3	37.5	0.003
regulation of cell size	3	37.5	0.003
endoplasmic reticulum unfolded protein response	4	23.5	0.004
signal transduction	16	2.1	0.004
urogenital system development	3	30.0	0.006
nucleic acid binding	12	2.0	0.008
retinoic acid receptor signaling pathway	3	27.3	0.008
protein phosphorylation	9	1.8	0.010
Neuroactive ligand-receptor interaction	4	33.3	0.013
vesicle docking involved in exocytosis	3	23.1	0.013
protein K63-linked deubiquitination	3	23.1	0.013
dorsal/ventral neural tube patterning	3	21.4	0.016
cellular response to oxidative stress	3	21.4	0.016
transferase activity, transferring phosphorus-containing groups	9	1.9	0.016
guanyl-nucleotide exchange factor activity	6	11.1	0.019
cell periphery	4	14.8	0.020
G-protein coupled receptor signaling pathway	8	1.9	0.022
one-carbon metabolic process	3	18.8	0.023
ATP metabolic process	3	18.8	0.023
positive regulation of transcription from RNA polymerase II promoter	9	2.0	0.027
protein kinase activity	9	1.9	0.027
transcription, DNA-dependent	4	1.4	0.028
ubiquitin ligase complex	4	13.3	0.029

hair follicle morphogenesis	3	16.7	0.032
positive regulation of fat cell differentiation	3	16.7	0.032
heart looping	4	12.9	0.032
regulation of pH	3	15.8	0.036
protein tyrosine kinase activity	8	2.0	0.038
oxidoreductase activity	5	10.4	0.039
metallocarboxypeptidase activity	3	15.0	0.042
heart morphogenesis	4	11.8	0.043

*Percentage of the genes of the pathway which were among the annotated genes.
