# Supplementary material for: Genome Scan for Selection in Structured Layer Chicken Populations Exploiting Linkage Disequilibrium Information
Source: PLoS One. 2015 Jul 7;10(7):e0130497. doi: 10.1371/journal.pone.0130497 (PMC4494984; doi:10.1371/journal.pone.0130497)
Supplement: S7 Table — (PDF) [file pone.0130497.s009.pdf]

Supplementary Table 7. Lists of pathways and gene ontologies under selection with FLK with 0.05% threshold in brown layers.

Description	# Genes anotated	Genes of pathways (%)*	P-Value
histone mRNA catabolic process	3	33.3	0.000
phosphoprotein binding	3	13.6	0.003
peptidyl-serine phosphorylation	4	7.8	0.006
stress fiber	3	7.7	0.017
mTOR signaling pathway	3	10.0	0.029
Metabolic pathways	6	12.1	0.033

*Percentage of the genes of the pathway which were among the annotated genes.
