# Supplementary material for: Genome Scan for Selection in Structured Layer Chicken Populations Exploiting Linkage Disequilibrium Information
Source: PLoS One. 2015 Jul 7;10(7):e0130497. doi: 10.1371/journal.pone.0130497 (PMC4494984; doi:10.1371/journal.pone.0130497)
Supplement: S8 Table — All, White and Brown stands for studies with all layers, within white layers and within brown layers, respectively. (PDF) [file pone.0130497.s010.pdf]

Supplementary Table 8. Regions detected as putative selective sweeps by hapFLK method with upper (U) and lower (L) 0.05% threshold. All, White and Brown stands for studies with all layers, within white layers and within brown layers, respectively.

Chr	Start	End	hapFLK	Test
1	5,121,876	5,302,365	0.76	Brown-L
1	5,329,469	5,329,469	1.81	All-L
1	8,677,015	8,677,294	1.79	All-L
1	8,731,823	8,757,484	0.46	White-L
1	32,397,662	32,942,914	0.46	White-L
1	51,163,477	51,256,248	6.09	Brown-U
1	51,215,475	51,225,638	7.91	All-U
1	127,732,538	127,915,106	1.81	All-L
1	157,490,940	157,516,967	0.46	White-L
1	161,237,909	161,332,626	0.45	White-L
1	196,244,077	196,253,560	7.88	All-U
2	12,175,577	12,209,773	1.81	All-L
2	73,071,390	73,339,729	0.46	White-L
2	73,193,094	73,263,368	1.81	All-L
2	95,743,533	95,792,009	0.46	White-L
2	113,911,780	114,055,311	1.81	All-L
2	113,950,392	113,999,395	0.75	Brown-L
2	134,443,362	134,459,049	7.85	All-U
2	142,627,443	142,689,733	1.81	All-L
3	6,616,322	6,757,590	1.81	All-L
3	6,645,290	6,753,215	0.75	Brown-L
3	34,770,172	34,836,299	1.81	All-L
3	61,004,540	61,118,267	0.46	White-L
4	52,837,671	52,869,617	0.46	White-L
6	14,491,630	15,094,652	4.95	White-U
6	14,517,907	14,908,351	8.06	All-U
6	25,082,446	25,328,086	4.92	White-L
7	24,156,732	24,190,715	0.76	Brown-L
9	11,867,843	11,878,490	0.46	White-L
10	6,738,610	6,799,776	0.76	Brown-L
11	12,850,158	12,850,158	0.76	Brown-L
13	2,137,687	2,237,067	8.38	All-U, White-U
13	10,463,797	10,528,177	0.75	Brown-L
13	10,582,518	10,582,518	1.81	All-L
20	10,843,995	10,934,960	0.76	Brown-L
26	4,496,470	4,790,865	6.05	Brown-U
27	3,442,539	3,634,198	5.65	Brown-U
27	3,464,176	3,656,769	8.98	All-U
27	5,033,879	5,184,681	0.76	Brown-L
28	1,121,483	1,130,412	7.93	All-U
