# Supplementary material for: Genome Scan for Selection in Structured Layer Chicken Populations Exploiting Linkage Disequilibrium Information
Source: PLoS One. 2015 Jul 7;10(7):e0130497. doi: 10.1371/journal.pone.0130497 (PMC4494984; doi:10.1371/journal.pone.0130497)
Supplement: S9 Table — (PDF) [file pone.0130497.s011.pdf]

Supplementary Table 9. List of genes for selective sweeps detected with hapFLK with 0.05% threshold in all layers.

Chr	Start	End	Description	hapFLK
1	50719186	50733066	DMC1 dosage suppressor of mck1 homolog, meiosis-specific homologous recombination	7.91
1	50753266	50759412	KDEL receptor 3	7.91
1	50770322	50773091	Inward-rectifying potassium channel cKir2.3; Uncharacterized protein	7.91
1	50788491	50804577	casein kinase I isoform epsilon	7.91
1	50832960	50835917	Transcription factor MafF	7.91
1	50836439	50854357	85 kDa calcium-independent phospholipase A2	7.91
1	50856306	50864314	BAI1-associated protein 2-like 2	7.91
1	50865712	50873377	Monocarboxylate transporter 3	7.91
1	50911765	50921215	Transcription factor SOX-10	7.91
1	50924002	50927570	DNA-directed RNA polymerases I, II, and III subunit RPABC2	7.91
1	50931992	50950143	MICAL-like protein 1	7.91
1	50953763	50963376	Eukaryotic translation initiation factor 3 subunit L	7.91
1	50979163	50980997	galanin receptor type 3	7.91
1	51010961	51014093	Beta-galactoside-binding lectin	7.91
1	51026210	51029611	pyridoxal (pyridoxine, vitamin B6) phosphatase	7.91
1	51050593	51062352	lectin, galactoside-binding, soluble, 2	7.91
1	51065196	51067106	CDC42 effector protein (Rho GTPase binding) 1	7.91
1	51083236	51095519	caspase recruitment domain family, member 10	7.91
1	51099652	51110327	MFNG O-fucosylpeptide 3-beta-N-acetylglucosaminyltransferase	7.91
1	51229151	51248424	cytohesin-4	7.91
1	51257450	51266551	ras-related C3 botulinum toxin substrate 2	7.91
1	51269724	51274129	somatostatin receptor type 3	7.91
1	51336790	51344019	potassium channel tetramerisation domain containing 17	7.91
1	51349989	51355027	Sulfurtransferase	7.91
1	51355166	51362344	thiosulfate sulfurtransferase	7.91
1	51419978	51430209	neutrophil cytosol factor 4	7.91
1	51455040	51463393	Parvalbumin, muscle	7.91
1	51573280	51580672	thioredoxin, mitochondrial	7.91
1	51598550	51666512	myosin-9	7.91
1	50983576	50984295	noggin 4 precursor	7.91
1	50991976	50992548	Histone H5	7.91
1	51511551	51511715	TUC338	7.91
1	5715214	5839991	CUGBP Elav-like family member 2	1.79
1	8177104	8318907	Semaphorin-3D	1.79
1	8522175	8850302	semaphorin-3A	1.79
1	127508430	127539291	protein kinase, X-linked	1.81
1	127679910	127698770	matrix-remodelling associated 5	1.81
1	127805905	127818492	arylsulfatase H precursor	1.81
1	127871298	127881303	glycogenin 2	1.81

1	127916919	127941663	CD99 antigen precursor	1.81
2	134048833	134252747	trichorhinophalangeal syndrome I	7.85
2	134628301	134709262	Eukaryotic translation initiation factor 3 subunit H	7.85
2	134749874	134773800	double-strand-break repair protein rad21 homolog	7.85
2	11735794	11743710	Krueppel-like factor 6	1.81
2	73536943	73537003	Small nucleolar RNA R11/Z151	1.81
2	113502155	113517713	tocopherol (alpha) transfer protein	1.81
2	113543309	113551391	YTH domain family protein 3	1.81
2	114472659	114503139	Armadillo repeat-containing protein 1	1.81
2	113888475	113888659	TUC338	1.81
2	142700486	142776708	KH domain containing, RNA binding, signal transduction associated 3	1.81
3	6450555	7094776	neurexin-1-alpha isoform 1 precursor	1.81
3	34322339	34455636	v-akt murine thymoma viral oncogene homolog 3 (protein kinase B, gamma)	1.81
3	34464087	34563969	serologically defined colon cancer antigen 8	1.81
3	34813540	34977620	inactive phospholipase D5	1.81
3	35065030	35080589	exonuclease 1	1.81
3	35088960	35147574	WD repeat domain 64	1.81
3	35238582	35471485	regulator of G-protein signaling 7	1.81
3	35212222	35213478	GPI mannosyltransferase 1	1.81
6	14411231	14417212	voltage-dependent anion-selective channel protein 2	8.06
6	14522141	14541652	Dual specificity phosphatase DUPD1	8.06
6	14667844	14924979	adenosine kinase	8.06
6	14941845	14956153	AP-3 complex subunit mu-1	8.06
6	14961440	14990291	Vinculin	8.06
6	15060198	15068452	urokinase-type plasminogen activator preproprotein	8.06
13	1767729	1785069	endothelial cell surface expressed chemotaxis and apoptosis regulator	8.38
13	1788284	1799938	dnaJ homolog subfamily C member 18	8.38
13	1806725	1808111	marginal zone B and B1 cell-specific protein	8.38
13	1813947	1819364	Polyadenylate-binding protein-interacting protein 2	8.38
13	1825981	1851149	matrin-3	8.38
13	2010626	2012530	leucine rich repeat transmembrane neuronal 2	8.38
13	2134785	2156387	stress-70 protein, mitochondrial precursor	8.38
13	2567435	2611832	fibroblast growth factor 18 precursor	8.38
13	2641231	2651587	nucleophosmin	8.38
13	2681554	2828621	RAN binding protein 17	8.38
13	2146299	2146368	Small nucleolar RNA SNORD63	8.38
13	10000421	10264193	transcription factor COE1	1.81
13	10554868	10603091	clathrin interactor 1	1.81
13	10618933	10623222	LSM11, U7 small nuclear RNA associated	1.81
13	10624187	10630514	probable tRNA(His) guanylyltransferase	1.81
13	10642122	10669352	disintegrin and metalloproteinase domain-containing protein 19 precursor	1.81
13	10668257	10680834	NIPA-like domain containing 4	1.81
13	10737034	10764716	IL2-inducible T-cell kinase	1.81

13	10778915	10785137	hepatitis A virus cellular receptor 1 precursor	1.81
27	3080955	3092426	histone acetyltransferase MYST2	8.98
27	3121453	3124525	solute carrier family 35 member B1	8.98
27	3204183	3219385	tumor necrosis factor receptor superfamily member 16 precursor	8.98
27	3252521	3267354	membrane protein, palmitoylated 3 (MAGUK p55 subfamily member 3)	8.98
27	3315353	3321406	homeobox protein MOX-1	8.98
27	3335561	3350183	ets variant 4	8.98
27	3352213	3364975	DEAH (Asp-Glu-Ala-His) box polypeptide 8	8.98
27	3366784	3370919	Prohibitin	8.98
27	3413412	3417057	phosphoethanolamine/phosphocholine phosphatase	8.98
27	3423443	3425719	guanine nucleotide binding protein , gamma transducing activity polypeptide 2	8.98
27	3433057	3459259	Insulin-like growth factor 2 mRNA-binding protein 1	8.98
27	3468107	3475761	gastric inhibitory polypeptide precursor	8.98
27	3495909	3506537	calcium binding and coiled-coil domain 2	8.98
27	3586132	3589844	Hoxb-7	8.98
27	3598354	3600970	homeobox B6	8.98
27	3604171	3606516	Homeobox protein Hox-B5	8.98
27	3621645	3626317	homeobox protein Hox-B4	8.98
27	3643412	3649970	homeobox protein Hox-B3	8.98
27	3652538	3655356	homeobox B2	8.98
27	3662963	3664600	homeobox B1	8.98
27	3711055	3815879	src kinase associated phosphoprotein 1	8.98
27	3842373	3850380	chromobox protein homolog 1	8.98
27	3855203	3861688	Nuclear factor erythroid 2-related factor 1	8.98
27	3865615	3868783	CDK5 regulatory subunit associated protein 3	8.98
27	3896638	3899664	leucine rich repeat containing 46	8.98
27	3902862	3907297	oxysterol binding protein-like 7	8.98
27	3971094	4005910	myeloid/lymphoid or mixed-lineage leukemia ; translocated to, 6	8.98
27	4006813	4007489	CDGSH iron sulfur domain 3	8.98
27	4008146	4011942	polycomb group ring finger 2	8.98
27	4014223	4017030	Proteasome subunit beta type	8.98
27	4046906	4048844	ribosomal protein L23	8.98
27	4053819	4068552	LIM and SH3 domain protein 1	8.98
27	3869421	3870521	proline rich 15-like	8.98
27	3155867	3156043	TUC338	8.98
27	4047757	4047888	Small nucleolar RNA SNORA21	8.98
28	627972	628532	translocase of inner mitochondrial membrane 13 homolog (yeast)	7.93
28	636058	661825	lamin-B2	7.93
28	676818	677614	60S ribosomal protein L36	7.93
28	678763	694768	lon peptidase 1, mitochondrial	7.93
28	701484	724473	solute carrier family 1 (high affinity aspartate/glutamate transporter), member 6	7.93
28	746840	778361	RAN binding protein 3	7.93
28	789271	792789	kelch-like family member 33	7.93

28	803320	808851	butyrophilin subfamily 1 member A1 precursor	7.93
28	832292	846222	ras-related protein Rab-11B	7.93
28	877452	886036	KN motif and ankyrin repeat domains 3	7.93
28	899423	901661	anti-dorsalizing morphogenetic protein precursor	7.93
28	932572	950125	ELAV-like protein 1	7.93
28	961655	965629	megakaryocyte-associated tyrosine kinase	7.93
28	972503	976394	Retinal homeobox protein Rx1	7.93
28	976369	983104	mucosa-associated lymphoid tissue lymphoma translocation protein 1-like	7.93
28	991289	1002123	tight junction protein 3	7.93
28	1036061	1041471	cactin, spliceosome C complex subunit	7.93
28	1041856	1045661	thromboxane A2 receptor	7.93
28	1047302	1049378	GIPC PDZ domain containing family, member 3	7.93
28	1049765	1054525	high mobility group 20B	7.93
28	1086890	1089949	Deoxyhypusine hydroxylase	7.93
28	1102700	1127154	unc-13 homolog A (C. elegans)	7.93
28	1143774	1145084	phosphatidic acid phosphatase type 2C	7.93
28	1171971	1179236	amino-terminal enhancer of split	7.93
28	1195931	1202586	guanine nucleotide-binding protein subunit alpha-11	7.93
28	1218202	1225645	nicalin precursor	7.93
28	1233711	1264738	CUGBP, Elav-like family member 5	7.93
28	1266179	1271107	Hydroxysteroid 11-beta-dehydrogenase 1-like protein	7.93
28	1303285	1407097	MPN domain containing	7.93
28	1414197	1415393	Zinc finger and BTB domain-containing protein 7A	7.93
28	1420886	1434153	protein inhibitor of activated STAT, 4	7.93
28	1438607	1446530	elongation factor 2	7.93
28	1447842	1453358	death-associated protein kinase 3	7.93
28	1455327	1459014	nicotinamide riboside kinase 2	7.93
28	1463358	1478145	ataxia, cerebellar, Cayman type	7.93
28	1507549	1509672	neurturin	7.93
28	1442292	1442361	Small nucleolar RNA SNORD37	7.93
