# Supplementary material for: Genome Scan for Selection in Structured Layer Chicken Populations Exploiting Linkage Disequilibrium Information
Source: PLoS One. 2015 Jul 7;10(7):e0130497. doi: 10.1371/journal.pone.0130497 (PMC4494984; doi:10.1371/journal.pone.0130497)
Supplement: S10 Table — (PDF) [file pone.0130497.s012.pdf]

Supplementary Table 10. List of genes for selective sweeps detected with hapFLK with 0.05% threshold in white layers.

Chr	Start	End	Description	hapFLK
1	8177104	8318907	Semaphorin-3D	0.46
1	8522175	8850302	semaphorin-3A	0.46
1	32863934	32927887	ubiquitin carboxyl-terminal hydrolase 15 isoform 1	0.46
1	32942077	33016611	protein MON2 homolog	0.46
1	33019230	33019319	gga-let-7i	0.46
1	156898969	157114305	kelch-like family member 1	0.46
1	161506641	161608657	tudor domain-containing protein 3	0.45
1	161696600	161932705	diaphanous homolog 3 (Drosophila)	0.45
2	73536943	73537003	Small nucleolar RNA R11/Z151	0.46
2	95477061	95554752	cadherin-7 precursor	0.46
3	60548727	60598139	clavesin 2	0.46
3	60674294	60677648	Fatty acid-binding protein, brain	0.46
3	60687661	60743857	protein kinase (cAMP-dependent, catalytic) inhibitor beta	0.46
3	60764156	60778623	serine incorporator 1 precursor	0.46
3	60779761	60800976	Heat shock factor protein 2	0.46
3	61222961	61230461	gap junction alpha-1 protein	0.46
4	52775238	52982663	protein sprouty homolog 1	0.46
4	52983308	52995163	nudix (nucleoside diphosphate linked moiety X)-type motif 6	0.46
4	52994729	53020450	Fibroblast growth factor 2	0.46
4	53097087	53101842	interleukin 21 precursor	0.46
4	53134199	53137244	interleukin 2 precursor	0.46
4	53155161	53264068	KIAA1109	0.46
4	53335689	53350682	Bardet-Biedl syndrome 7	0.46
4	53350909	53356114	cyclin A2	0.46
6	14411231	14417212	voltage-dependent anion-selective channel protein 2	4.95
6	14522141	14541652	Dual specificity phosphatase DUPD1	4.95
6	14667844	14924979	adenosine kinase	4.95
6	14941845	14956153	AP-3 complex subunit mu-1	4.95
6	14961440	14990291	Vinculin	4.95
6	15060198	15068452	urokinase-type plasminogen activator preproprotein	4.95
6	24664740	24940362	sortilin-related VPS10 domain containing receptor 1	4.95
6	25264735	25352280	gamma-adducin	4.92
6	25366993	25409141	max-interacting protein 1	4.92
6	25517714	25527369	Dual specificity protein phosphatase	4.92
6	25533210	25555268	structural maintenance of chromosomes protein 3	4.92
6	25683192	25695299	programmed cell death protein 4	4.92
9	11379753	11384969	Zic family member 4	0.46
9	11938121	11940148	phosphatidylinositol glycan anchor biosynthesis, class Z	0.46
9	11940276	11956868	melanotransferrin precursor	0.46

9	11959073	12089444	discs, large homolog 1 (Drosophila)	0.46
9	12115163	12127577	D-beta-hydroxybutyrate dehydrogenase, mitochondrial precursor	0.46
9	12153804	12156739	apolipoprotein D precursor	0.46
9	12157987	12170689	protein phosphatase inhibitor 2	0.46
9	12171478	12231009	Arf-GAP with coiled-coil, ANK repeat and PH domain-containing protein 2	0.46
9	12296460	12308388	large subunit GTPase 1 homolog	0.46
9	11878868	11880012	Type-1 angiotensin II receptor	0.46
13	1767729	1785069	endothelial cell surface expressed chemotaxis and apoptosis regulator	5.33
13	1788284	1799938	dnaJ homolog subfamily C member 18	5.33
13	1806725	1808111	marginal zone B and B1 cell-specific protein	5.33
13	1813947	1819364	Polyadenylate-binding protein-interacting protein 2	5.33
13	1825981	1851149	matrin-3	5.33
13	2010626	2012530	leucine rich repeat transmembrane neuronal 2	5.33
13	2134785	2156387	stress-70 protein, mitochondrial precursor	5.33
13	2567435	2611832	fibroblast growth factor 18 precursor	5.33
13	2641231	2651587	nucleophosmin	5.33
13	2681554	2828621	RAN binding protein 17	5.33
13	2146299	2146368	Small nucleolar RNA SNORD63	5.33
