# Supplementary material for: Genome Scan for Selection in Structured Layer Chicken Populations Exploiting Linkage Disequilibrium Information
Source: PLoS One. 2015 Jul 7;10(7):e0130497. doi: 10.1371/journal.pone.0130497 (PMC4494984; doi:10.1371/journal.pone.0130497)
Supplement: S11 Table — (PDF) [file pone.0130497.s013.pdf]

Supplementary Table 11. List of genes for selective sweeps detected with hapFLK with 0.05% threshold in brown layers.

Chr	Start	End	Description	hapFLK
1	50719186	50733066	DMC1 dosage suppressor of mck1 homolog, meiosis-specific homologous recombination	6.09
1	50753266	50759412	KDEL receptor 3	6.09
1	50770322	50773091	Inward-rectifying potassium channel cKir2.3; Uncharacterized protein	6.09
1	50788491	50804577	casein kinase I isoform epsilon	6.09
1	50832960	50835917	Transcription factor MafF	6.09
1	50836439	50854357	85 kDa calcium-independent phospholipase A2	6.09
1	50856306	50864314	BAI1-associated protein 2-like 2	6.09
1	50865712	50873377	Monocarboxylate transporter 3	6.09
1	50911765	50921215	Transcription factor SOX-10	6.09
1	50924002	50927570	DNA-directed RNA polymerases I, II, and III subunit RPABC2	6.09
1	50931992	50950143	MICAL-like protein 1	6.09
1	50953763	50963376	Eukaryotic translation initiation factor 3 subunit L	6.09
1	50979163	50980997	galanin receptor type 3	6.09
1	51010961	51014093	Beta-galactoside-binding lectin	6.09
1	51026210	51029611	pyridoxal (pyridoxine, vitamin B6) phosphatase	6.09
1	51050593	51062352	lectin, galactoside-binding, soluble, 2	6.09
1	51065196	51067106	CDC42 effector protein (Rho GTPase binding) 1	6.09
1	51083236	51095519	caspase recruitment domain family, member 10	6.09
1	51099652	51110327	MFNG O-fucosylpeptide 3-beta-N-acetylglucosaminyltransferase	6.09
1	51229151	51248424	cytohesin-4	6.09
1	51257450	51266551	ras-related C3 botulinum toxin substrate 2	6.09
1	51269724	51274129	somatostatin receptor type 3	6.09
1	51336790	51344019	potassium channel tetramerisation domain containing 17	6.09
1	51349989	51355027	Sulfurtransferase	6.09
1	51355166	51362344	thiosulfate sulfurtransferase	6.09
1	51419978	51430209	neutrophil cytosol factor 4	6.09
1	51455040	51463393	Parvalbumin, muscle	6.09
1	51573280	51580672	thioredoxin, mitochondrial	6.09
1	51598550	51666512	myosin-9	6.09
1	50983576	50984295	noggin 4 precursor	6.09
1	50991976	50992548	Histone H5	6.09
1	51511551	51511715	TUC338	6.09
2	113502155	113517713	tocopherol (alpha) transfer protein	0.75
2	113543309	113551391	YTH domain family protein 3	0.75
2	114472659	114503139	Armadillo repeat-containing protein 1	0.75
2	113888475	113888659	TUC338	0.75
3	6450555	7094776	neurexin-1-alpha isoform 1 precursor	0.75
7	23919267	24182349	Contactin-associated protein-like 5	0.76
10	6234433	6311306	myosin IE	0.76

10	6313607	6321500	G2/mitotic-specific cyclin-B2	0.76
10	6322555	6364720	E3 ubiquitin-protein ligase Arkadia	0.76
10	6377333	6395463	SAFB-like, transcription modulator	0.76
10	6434758	6619897	disintegrin and metalloproteinase domain-containing protein 10 precursor	0.76
10	6480269	6521283	lipase, hepatic	0.76
10	6572777	6596733	aquaporin 9	0.76
10	6604818	6661741	Retinal dehydrogenase 2	0.76
10	6816944	6857906	cingulin-like 1	0.76
10	6903270	7058903	transcription factor 12	0.76
10	7158297	7164937	meiosis-specific nuclear structural 1	0.76
10	7159553	7183472	testis expressed 9	0.76
10	7249251	7256715	regulatory factor X, 7	0.76
10	7266832	7316503	E3 ubiquitin-protein ligase	0.76
11	12288497	12432361	cadherin-8	0.76
11	13255162	13323250	ADAM metallopeptidase with thrombospondin type 1 motif, 18	0.76
11	12638991	12639143	TUC338	0.76
13	10000421	10264193	transcription factor COE1	0.75
13	10554868	10603091	clathrin interactor 1	0.75
13	10618933	10623222	LSM11, U7 small nuclear RNA associated	0.75
13	10624187	10630514	probable tRNA(His) guanylyltransferase	0.75
13	10642122	10669352	disintegrin and metalloproteinase domain-containing protein 19 precursor	0.75
13	10668257	10680834	NIPA-like domain containing 4	0.75
13	10737034	10764716	IL2-inducible T-cell kinase	0.75
13	10778915	10785137	hepatitis A virus cellular receptor 1 precursor	0.75
20	10382093	10392964	targeting protein for Xklp2	0.76
20	10395495	10403016	myosin light chain kinase 2, skeletal/cardiac muscle	0.76
20	10405912	10410873	interferon regulatory factor 10	0.76
20	10412313	10419874	dual specificity phosphatase 15	0.76
20	10420572	10431015	tubulin tyrosine ligase-like family, member 9	0.76
20	10431992	10433839	p53 and DNA-damage regulated 1	0.76
20	10487518	10491477	GDP-fucose protein O-fucosyltransferase 1 precursor	0.76
20	10492514	10502873	kinesin-like protein KIF3B	0.76
20	10505808	10517025	additional sex combs like 1 (Drosophila)	0.76
20	10595952	10609143	DNA (cytosine-5-)-methyltransferase 3 beta	0.76
20	10610165	10617328	Microtubule-associated protein RP/EB family member 1	0.76
20	10633103	10644913	Ovocalyxin-36 precursor	0.76
20	10643158	10647278	protein TENP	0.76
20	10649024	10653537	BPI fold containing family B, member 6	0.76
20	10663664	10668879	BPI fold containing family B, member 4	0.76
20	10700368	10707694	KIAA1755	0.76
20	10714657	10723053	protein-glutamine gamma-glutamyltransferase 2	0.76
20	10726664	10747756	regulation of nuclear pre-mRNA domain-containing protein 1B	0.76
20	10772139	10813719	catenin, beta like 1	0.76

20	10849414	10853705	deoxynucleotidyltransferase, terminal, interacting protein 1	0.76
20	10856323	10858120	Troponin C, skeletal muscle	0.76
20	10871289	10873861	neuralized homolog 2 (Drosophila)	0.76
20	10874294	10878191	lysosomal protective protein precursor	0.76
20	10878671	10881926	phospholipid transfer protein precursor	0.76
20	10887208	10895373	PDX1 C-terminal inhibiting factor 1	0.76
20	10917368	10921453	matrix metalloproteinase-9 precursor	0.76
20	10922030	10944646	solute carrier family 12 (potassium/chloride transporter), member 5	0.76
20	10966821	10970503	tumor necrosis factor receptor superfamily member 5 precursor	0.76
20	11071603	11076564	solute carrier family 35 member C2	0.76
26	4133855	4213476	ankyrin repeat and sterile alpha motif domain containing 1A	6.05
26	4214176	4217459	transcription initiation factor TFIID subunit 11	6.05
26	4248039	4252009	U1 small nuclear ribonucleoprotein C	6.05
26	4302444	4306682	protein kinase C and casein kinase substrate in neurons 1	6.05
26	4380152	4414660	glutamate receptor, metabotropic 4	6.05
26	4496441	4502013	Green-sensitive opsin	6.05
26	4515351	4518565	Motilin	6.05
26	4540474	4553563	inositol hexakisphosphate kinase 3	6.05
26	4560707	4598750	inositol 1,4,5-trisphosphate receptor, type 3	6.05
26	4657007	4660254	O-acetyl-ADP-ribose deacetylase C6orf130 homolog	6.05
26	4660732	4674975	nuclear transcription factor Y subunit alpha	6.05
26	4678907	4684706	Triggering receptor expressed on myeloid cells; Uncharacterized protein	6.05
26	4686588	4691130	triggering receptor expressed on myeloid cells 2 precursor	6.05
26	4693285	4699278	triggering receptor expressed on myeloid cells precursor	6.05
26	4891507	4916817	forkhead box P4	6.05
26	4960037	4974337	transcription factor EB	6.05
26	4976148	4979037	progastricsin (pepsinogen C)	6.05
26	4980962	4984806	gastricsin precursor	6.05
26	4989811	4997320	fibroblast growth factor receptor substrate 3	6.05
26	5001119	5006673	prickle homolog 4 (Drosophila)	6.05
26	5028663	5038433	Ubiquitin carboxyl-terminal hydrolase	6.05
26	5056220	5060765	Mediator of RNA polymerase II transcription subunit 20	6.05
26	5060817	5063516	bystin-like	6.05
26	5065132	5077551	G1/S-specific cyclin-D3	6.05
26	5103140	5110535	Transcription initiation factor TFIID subunit 8	6.05
26	5112283	5114932	primary cilia formation	6.05
26	5122698	5126812	acidic chitinase precursor	6.05
27	3080955	3092426	histone acetyltransferase MYST2	5.65
27	3121453	3124525	solute carrier family 35 member B1	5.65
27	3204183	3219385	tumor necrosis factor receptor superfamily member 16 precursor	5.65
27	3252521	3267354	membrane protein, palmitoylated 3 (MAGUK p55 subfamily member 3)	5.65
27	3315353	3321406	homeobox protein MOX-1	5.65
27	3335561	3350183	ets variant 4	5.65

27	3352213	3364975	DEAH (Asp-Glu-Ala-His) box polypeptide 8	5.65
27	3366784	3370919	Prohibitin	5.65
27	3413412	3417057	phosphoethanolamine/phosphocholine phosphatase	5.65
27	3423443	3425719	guanine nucleotide binding protein (G protein), gamma transducing activity polypeptide 2	5.65
27	3433057	3459259	Insulin-like growth factor 2 mRNA-binding protein 1	5.65
27	3468107	3475761	gastric inhibitory polypeptide precursor	5.65
27	3495909	3506537	calcium binding and coiled-coil domain 2	5.65
27	3586132	3589844	Hoxb-7	5.65
27	3598354	3600970	homeobox B6	5.65
27	3604171	3606516	Homeobox protein Hox-B5	5.65
27	3621645	3626317	homeobox protein Hox-B4	5.65
27	3643412	3649970	homeobox protein Hox-B3	5.65
27	3652538	3655356	homeobox B2	5.65
27	3662963	3664600	homeobox B1	5.65
27	3711055	3815879	src kinase associated phosphoprotein 1	5.65
27	3842373	3850380	chromobox protein homolog 1	5.65
27	3855203	3861688	Nuclear factor erythroid 2-related factor 1	5.65
27	3865615	3868783	CDK5 regulatory subunit associated protein 3	5.65
27	3896638	3899664	leucine rich repeat containing 46	5.65
27	3902862	3907297	oxysterol binding protein-like 7	5.65
27	3971094	4005910	myeloid/lymphoid or mixed-lineage leukemia ; translocated to, 6	5.65
27	4006813	4007489	CDGSH iron sulfur domain 3	5.65
27	4008146	4011942	polycomb group ring finger 2	5.65
27	4014223	4017030	Proteasome subunit beta type	5.65
27	3869421	3870521	proline rich 15-like	5.65
27	3155867	3156043	TUC338	5.65
27	4685681	4690379	Small nuclear ribonucleoprotein-associated protein B	0.76
27	4698639	4700783	kelch-like family member 11	0.76
27	4701712	4720355	ATP-citrate synthase	0.76
27	4739049	4758336	dnaJ homolog subfamily C member 7	0.76
27	4758782	4759794	NF-kappa-B inhibitor-interacting Ras-like protein 2	0.76
27	4822592	4826644	DEXH (Asp-Glu-X-His) box polypeptide 58	0.76
27	4827918	4832197	histone acetyltransferase KAT2A	0.76
27	4837933	4841685	Ras-related protein Rab-5C	0.76
27	4847654	4856867	potassium voltage-gated channel, subfamily H (eag-related), member 4	0.76
27	4857256	4858313	hypocretin (orexin) neuropeptide precursor	0.76
27	4871019	4873562	GH3 domain containing	0.76
27	4875164	4886606	signal transducer and activator of transcription 5B	0.76
27	4896267	4907552	Signal transducer and activator of transcription 3	0.76
27	4913996	4926852	polymerase I and transcript release factor	0.76
27	4929945	4958104	V-type proton ATPase 116 kDa subunit a isoform 1	0.76
27	4962219	4963268	17-beta-hydroxysteroid dehydrogenase	0.76
27	4963812	4965610	CoA synthase	0.76

27	4967021	4970406	max-like protein X	0.76
27	4970565	4973603	PSMC3 interacting protein	0.76
27	4990263	4995457	pleckstrin homology domain containing, family H (with MyTH4 domain) member 3	0.76
27	5003802	5012710	contactin associated protein 1	0.76
27	5030186	5032034	receptor activity-modifying protein 2 precursor	0.76
27	5039150	5051893	WNK lysine deficient protein kinase 4	0.76
27	5055739	5060519	beclin-1	0.76
27	5060694	5067398	Proteasome activator complex subunit 3	0.76
27	5095037	5098091	RUN domain containing 1	0.76
27	5099248	5101501	60S ribosomal protein L27	0.76
27	5102844	5105307	interferon-induced protein 35	0.76
27	5126612	5140086	rho-related GTP-binding protein RhoN	0.76
27	5150701	5171059	breast cancer 1, early onset	0.76
27	5171677	5189435	neighbor of BRCA1 gene 1	0.76
27	5192609	5198967	membrane protein, palmitoylated 2 (MAGUK p55 subfamily member 2)	0.76
27	5000132	5001181	chemokine (C-C motif) receptor 10	0.76
27	5173809	5173871	Neighbour of BRCA1 gene 2 converved region	0.76
