# Supplementary material for: Genome Scan for Selection in Structured Layer Chicken Populations Exploiting Linkage Disequilibrium Information
Source: PLoS One. 2015 Jul 7;10(7):e0130497. doi: 10.1371/journal.pone.0130497 (PMC4494984; doi:10.1371/journal.pone.0130497)
Supplement: S12 Table — (PDF) [file pone.0130497.s014.pdf]

Supplementary Table 12. Lists of pathways and gene ontologies under selection with hapFLK with 0.05% threshold in all layers.

Description	# Genes anotated	Genes of pathways (%)*	P-Value
protein binding	2	2.0	0.000
apical junction assembly	2	28.6	0.000
epithelial cell-cell adhesion	2	28.6	0.000
cadherin binding	2	14.3	0.001
adherens junction	2	10.5	0.001
cell-cell adherens junction	2	9.1	0.002
mitochondrial nucleoid	2	7.4	0.002
integrin activation	1	50.0	0.005
intracellular organelle	1	50.0	0.005
L-ascorbic acid transport	1	50.0	0.005
L-ascorbic acid transporter activity	1	50.0	0.005
mediator complex binding	1	50.0	0.005
meiotic cohesin complex	1	50.0	0.005
negative regulation of integrin-mediated signaling pathway	1	50.0	0.005
positive regulation of RNA splicing	1	50.0	0.005
regulation of endodeoxyribonuclease activity	1	50.0	0.005
regulation of relaxation of cardiac muscle	1	50.0	0.005
sodium-dependent L-ascorbate transmembrane transporter activity	1	50.0	0.005
transepithelial L-ascorbic acid transport	1	50.0	0.005
beta-catenin binding	2	4.7	0.006
nucleosome assembly	2	4.3	0.007
bradykinin catabolic process	1	33.3	0.008
calcium-dependent cell-cell adhesion	1	33.3	0.008
cellular response to indole-3-methanol	1	33.3	0.008
dehydroascorbic acid transporter activity	1	33.3	0.008
endoplasmic reticulum chaperone complex	1	33.3	0.008
negative regulation of DNA endoreduplication	1	33.3	0.008
nuclear meiotic cohesin complex	1	33.3	0.008
regulation of B cell proliferation	1	33.3	0.008
regulation of receptor activity	1	33.3	0.008
translation release factor activity, codon specific	1	33.3	0.008
type 1 fibroblast growth factor receptor binding	1	33.3	0.008
type 2 fibroblast growth factor receptor binding	1	33.3	0.008
zonula adherens	1	33.3	0.008
adherens junction assembly	1	25.0	0.011
AMP biosynthetic process	1	25.0	0.011
dehydroascorbic acid transport	1	25.0	0.011
fibrinolysis	1	25.0	0.011
negative regulation of cell motility	1	25.0	0.011

negative regulation of centrosome duplication	1	25.0	0.011
negative regulation of protein kinase activity by regulation of protein phosphorylation	1	25.0	0.011
regulation of smooth muscle cell migration	1	25.0	0.011
smooth muscle cell migration	1	25.0	0.011
translation repressor activity	1	25.0	0.011
translational termination	1	25.0	0.011
voltage-gated anion channel activity	1	25.0	0.011
alpha-catenin binding	1	20.0	0.013
dystroglycan binding	1	20.0	0.013
labyrinthine layer development	1	20.0	0.013
MOZ/MORF histone acetyltransferase complex	1	20.0	0.013
negative regulation of neuroblast proliferation	1	20.0	0.013
negative regulation of translational initiation	1	20.0	0.013
neuron fate specification	1	20.0	0.013
purine ribonucleoside salvage	1	20.0	0.013
regulation of centriole replication	1	20.0	0.013
regulation of respiratory gaseous exchange by neurological system process	1	20.0	0.013
Tat protein binding	1	20.0	0.013
calmodulin-dependent protein kinase activity	1	16.7	0.016
catenin complex	1	16.7	0.016
establishment or maintenance of cell polarity	1	16.7	0.016
lateral element	1	16.7	0.016
mitotic spindle organization	1	16.7	0.016
ribosomal small subunit binding	1	16.7	0.016
spindle pole centrosome	1	16.7	0.016
cell-cell junction	2	2.7	0.017
gamma-catenin binding	1	14.3	0.019
regulation of anion transport	1	14.3	0.019
phosphotransferase activity, alcohol group as acceptor	2	2.4	0.021
MAP kinase tyrosine/serine/threonine phosphatase activity	1	12.5	0.021
O-methyltransferase activity	1	12.5	0.021
positive regulation of extrinsic apoptotic signaling pathway in absence of ligand	1	12.5	0.021
protein kinase inhibitor activity	1	12.5	0.021
regulation of DNA replication	1	12.5	0.021
vinculin binding	1	12.5	0.021
acetyltransferase activity	1	11.1	0.024
costamere	1	11.1	0.024
regulation of cell adhesion mediated by integrin	1	11.1	0.024
regulation of cell proliferation	2	2.2	0.024
structural molecule activity	2	2.2	0.025
fascia adherens	1	10.0	0.027
MAP kinase activity	1	10.0	0.027

dynein binding	1	9.1	0.029
meiosis	1	9.1	0.029
actin cytoskeleton	2	2.0	0.030
nucleoplasm	2	1.9	0.032
basal plasma membrane	1	8.3	0.032
ceramide biosynthetic process	1	8.3	0.032
protein destabilization	1	8.3	0.032
SCF-dependent proteasomal ubiquitin-dependent protein catabolic process	1	8.3	0.032
anterograde synaptic vesicle transport	1	7.7	0.035
morphogenesis of an epithelium	1	7.7	0.035
respiratory gaseous exchange	1	7.7	0.035
nucleus	2	2.1	0.037
anterograde axon cargo transport	1	7.1	0.037
glycogen metabolic process	1	7.1	0.037
protein export from nucleus	1	7.1	0.037
protein localization to cell surface	1	7.1	0.037
anion transport	1	6.7	0.040
cell fate specification	1	6.7	0.040
histone acetylation	1	6.7	0.040
positive regulation of proteolysis	1	6.7	0.040
positive regulation of smoothened signaling pathway	1	6.7	0.040
protein dimerization activity	2	1.7	0.042
aminopeptidase activity	1	6.3	0.042
cell aging	1	6.3	0.042
RNA metabolic process	1	6.3	0.042
sodium ion transmembrane transport	1	6.3	0.042
negative regulation of intrinsic apoptotic signaling pathway	1	5.9	0.045
protein methylation	1	5.9	0.045
intercalated disc	1	5.6	0.048
negative regulation of extrinsic apoptotic signaling pathway in absence of ligand	1	5.6	0.048

*Percentage of the genes of the pathway which were among the annotated genes.
