# Supplementary material for: Genome Scan for Selection in Structured Layer Chicken Populations Exploiting Linkage Disequilibrium Information
Source: PLoS One. 2015 Jul 7;10(7):e0130497. doi: 10.1371/journal.pone.0130497 (PMC4494984; doi:10.1371/journal.pone.0130497)
Supplement: S14 Table — (PDF) [file pone.0130497.s016.pdf]

Supplementary Table 14. Lists of pathways and gene ontologies under selection with hapFLK with 0.05% threshold in brown layers.

Description	# Genes anotated	Genes of pathways (%)*	P-Value
RNA polymerase II transcription factor	2	18.2	0.005
negative regulation of MAPK cascade	2	16.7	0.005
immunological synapse	2	14.3	0.007
inward rectifier potassium channel activity	2	12.5	0.010
RNA polymerase II distal enhancer sequence-specific DNA binding	2	12.5	0.010
phosphatidylinositol metabolic process	2	11.8	0.011
cholesterol binding	2	10.0	0.015
ruffle membrane	2	9.5	0.016
phosphatidylinositol binding	3	4.8	0.021
sequence-specific DNA binding RNA polymerase II transcription	2	7.4	0.026
skeletal muscle cell differentiation	2	6.7	0.032
negative regulation of Wnt receptor signaling pathway	2	6.3	0.036
Rab GTPase binding	2	5.7	0.043

*Percentage of the genes of the pathway which were among the annotated genes.
