# Supplementary material for: Genome Scan for Selection in Structured Layer Chicken Populations Exploiting Linkage Disequilibrium Information
Source: PLoS One. 2015 Jul 7;10(7):e0130497. doi: 10.1371/journal.pone.0130497 (PMC4494984; doi:10.1371/journal.pone.0130497)
Supplement: S15 Table — LG stands for studies between commercial-layers and non-commercial chickens and BW stands for studies between brown and white layers, respectively. (PDF) [file pone.0130497.s017.pdf]

Supplementary Table 15. Regions detected as putative selective sweeps by F_{ST} method (Gholami *et al* 2014) with upper (U) and lower (L) 1% threshold. LG stands for studies between commercial-layers and non-commercial chickens and BW stands for studies between brown and white layers, respectively.

Chr	Start	End	F_{ST}	Test
1	1,193,561	2,160,406	0.04	LG-L
1	1,441,655	1,479,023	0.06	BW-L
1	5,179,370	6,229,653	0.05	LG-L
1	8,022,827	9,047,098	0.04	LG-L
1	8,421,962	8,845,934	0.05	BW-L
1	9,371,847	9,703,758	0.69	BW-U
1	9,398,724	9,688,938	0.26	LG-U
1	9,738,479	9,969,381	0.04	LG-L
1	9,859,154	9,881,817	0.08	BW-L
1	10,449,776	10,476,620	0.08	BW-L
1	10,716,550	10,841,257	0.05	LG-L
1	13,673,985	13,702,408	0.06	LG-L
1	14,050,347	14,084,859	0.06	BW-L
1	14,155,483	14,994,835	0.71	BW-U
1	14,161,900	14,194,912	0.22	LG-U
1	15,957,904	16,013,600	0.24	LG-U
1	16,775,514	17,072,787	0.05	LG-L
1	17,835,693	17,864,334	0.19	LG-U
1	17,978,421	18,020,939	0.05	LG-L
1	18,552,552	18,689,745	0.04	LG-L
1	18,560,115	18,683,424	0.05	BW-L
1	19,533,820	19,657,690	0.07	BW-L
1	19,679,055	19,708,643	0.06	LG-L
1	19,744,361	19,787,178	0.23	LG-U
1	21,515,856	21,669,265	0.05	LG-L
1	24,853,910	24,879,653	0.51	BW-U
1	25,442,648	26,636,946	0.75	BW-U
1	25,809,984	26,252,980	0.23	LG-U
1	27,414,647	27,444,302	0.07	BW-L
1	27,943,860	28,217,665	0.04	LG-L
1	28,186,828	28,308,289	0.02	BW-L
1	28,477,499	28,523,837	0.57	BW-U
1	29,182,974	29,221,914	0.21	LG-U
1	32,069,834	32,389,823	0.23	LG-U
1	32,075,632	32,103,663	0.53	BW-U
1	32,701,904	33,105,713	0.02	BW-L
1	32,721,392	32,962,939	0.04	LG-L

1	34,965,100	35,367,039	0.20	LG-U
1	35,898,597	35,946,312	0.61	BW-U
1	36,695,479	36,724,082	0.21	LG-U
1	37,303,787	37,333,726	0.55	BW-U
1	37,306,013	37,333,726	0.20	LG-U
1	38,323,846	38,445,567	0.55	BW-U
1	39,089,522	39,159,088	0.20	LG-U
1	39,091,445	39,109,935	0.54	BW-U
1	40,294,235	40,840,512	0.56	BW-U
1	41,340,808	41,508,154	0.21	LG-U
1	41,376,061	41,467,649	0.58	BW-U
1	41,637,416	41,687,234	0.05	BW-L
1	42,083,294	42,552,048	0.64	BW-U
1	43,122,131	43,561,291	0.64	BW-U
1	43,161,360	45,510,528	0.25	LG-U
1	43,368,113	43,419,637	0.05	BW-L
1	45,313,256	45,603,214	0.04	BW-L
1	46,322,686	46,546,913	0.26	LG-U
1	46,491,308	46,520,875	0.57	BW-U
1	46,608,479	46,648,538	0.08	BW-L
1	50,049,450	50,642,580	0.22	LG-U
1	50,111,749	50,153,489	0.58	BW-U
1	51,260,400	52,019,199	0.63	BW-U
1	51,747,030	52,016,343	0.21	LG-U
1	52,703,558	52,729,236	0.56	BW-U
1	53,066,372	53,080,103	0.06	LG-L
1	53,900,745	53,939,961	0.06	BW-L
1	54,246,372	54,273,416	0.54	BW-U
1	54,507,528	54,536,227	0.21	LG-U
1	55,764,654	55,804,334	0.20	LG-U
1	56,303,249	57,197,538	0.06	BW-L
1	56,527,871	56,556,097	0.05	LG-L
1	58,200,640	58,235,292	0.55	BW-U
1	58,363,617	58,956,853	0.21	LG-U
1	58,936,564	59,109,835	0.66	BW-U
1	59,873,363	59,888,158	0.19	LG-U
1	60,319,594	60,342,241	0.08	BW-L
1	60,359,702	60,480,934	0.53	BW-U
1	60,776,913	61,303,203	0.22	LG-U
1	61,152,158	61,195,236	0.69	BW-U
1	62,988,448	63,006,620	0.08	BW-L
1	63,029,709	63,443,749	0.20	LG-U
1	63,411,807	63,454,681	0.60	BW-U

1	64,471,369	64,496,349	0.51	BW-U
1	64,508,907	64,549,255	0.20	LG-U
1	66,018,488	66,162,576	0.25	LG-U
1	66,318,746	66,347,929	0.08	BW-L
1	66,828,581	66,869,716	0.62	BW-U
1	67,086,431	67,548,182	0.05	LG-L
1	67,396,266	67,415,782	0.07	BW-L
1	68,341,141	69,503,920	0.29	LG-U
1	68,399,482	68,896,858	0.58	BW-U
1	69,469,595	70,201,138	0.55	BW-U
1	70,148,357	70,185,499	0.22	LG-U
1	72,027,778	72,696,030	0.03	BW-L
1	72,619,478	73,142,186	0.22	LG-U
1	72,896,754	73,167,112	0.74	BW-U
1	76,226,444	76,792,359	0.23	LG-U
1	76,421,837	76,526,547	0.03	BW-L
1	79,066,419	79,092,157	0.20	LG-U
1	79,987,700	80,418,301	0.04	BW-L
1	81,260,697	81,731,439	0.04	LG-L
1	81,686,042	82,069,734	0.03	BW-L
1	84,323,244	86,151,250	0.22	LG-U
1	84,642,378	85,796,928	0.64	BW-U
1	86,464,220	86,488,605	0.05	LG-L
1	86,818,724	86,856,240	0.06	BW-L
1	87,642,571	88,092,210	0.04	LG-L
1	87,813,146	87,880,457	0.57	BW-U
1	88,722,419	88,768,126	0.04	BW-L
1	88,748,151	89,093,543	0.05	LG-L
1	89,966,169	89,990,508	0.06	BW-L
1	90,748,828	91,197,147	0.07	BW-L
1	90,886,679	91,164,114	0.05	LG-L
1	91,379,104	91,434,231	0.21	LG-U
1	91,381,218	91,415,282	0.55	BW-U
1	91,753,916	91,907,871	0.05	LG-L
1	91,761,220	91,794,078	0.06	BW-L
1	92,814,600	92,886,450	0.05	LG-L
1	93,001,292	93,029,051	0.52	BW-U,
1	93,823,292	96,765,628	0.03	LG-L
1	94,153,506	94,193,322	0.06	BW-L
1	94,360,597	94,432,393	0.82	BW-U
1	94,373,088	94,417,220	0.23	LG-U
1	95,291,969	95,367,844	0.07	BW-L
1	96,668,297	96,707,218	0.07	BW-L

1	97,602,431	99,084,382	0.03	LG-L
1	98,232,148	98,276,678	0.21	LG-U
1	98,284,551	98,820,373	0.03	BW-L
1	99,795,712	99,838,296	0.22	LG-U
1	99,807,511	100,221,026	0.59	BW-U
1	101,036,229	101,487,219	0.05	LG-L
1	101,444,303	101,563,985	0.07	BW-L
1	102,787,132	102,812,382	0.21	LG-U
1	102,991,881	103,017,289	0.51	BW-U
1	103,156,470	103,593,845	0.05	LG-L
1	104,312,359	104,538,878	0.03	LG-L
1	104,511,693	104,533,175	0.07	BW-L
1	106,034,326	106,052,643	0.06	LG-L
1	106,100,891	106,153,189	0.04	BW-L
1	107,776,597	108,136,657	0.23	LG-U, BW-U
1	108,636,886	108,959,832	0.21	LG-U
1	111,267,609	111,288,872	0.08	BW-L
1	111,267,609	111,527,698	0.05	LG-L
1	112,156,503	112,181,114	0.08	BW-L, LG-L
1	113,107,191	113,529,870	0.05	LG-L
1	113,809,905	113,828,375	0.20	LG-U
1	114,894,595	114,915,628	0.51	BW-U
1	115,476,285	115,523,891	0.21	LG-U
1	115,988,784	116,004,314	0.54	BW-U
1	116,503,940	117,648,686	0.28	LG-U
1	117,296,836	117,651,049	0.67	BW-U
1	117,438,382	117,462,220	0.05	LG-L
1	117,491,774	117,551,343	0.04	BW-L
1	119,028,106	120,145,853	0.04	BW-L
1	119,432,048	119,450,552	0.51	BW-U
1	119,608,449	119,635,306	0.05	LG-L
1	119,939,464	120,008,424	0.31	LG-U
1	122,501,717	122,537,196	0.21	LG-U
1	123,601,626	123,644,932	0.63	BW-U
1	124,259,017	124,342,556	0.58	BW-U
1	124,270,116	124,288,848	0.20	LG-U
1	124,824,287	124,845,348	0.08	BW-L
1	125,487,006	125,938,728	0.06	LG-L
1	126,449,811	128,670,033	0.04	LG-L
1	126,618,698	126,654,297	0.54	BW-U
1	127,236,038	127,748,123	0.03	BW-L
1	128,325,849	128,345,605	0.08	BW-L
1	130,191,226	131,019,604	0.21	LG-U

1	130,497,173	130,512,806	0.08	BW-L
1	132,033,893	132,065,465	0.07	BW-L
1	132,035,631	132,404,557	0.05	LG-L
1	133,744,269	133,893,092	0.21	LG-U
1	135,071,418	135,095,827	0.57	BW-U
1	135,642,106	135,662,576	0.08	BW-L
1	136,405,896	136,460,075	0.26	LG-U
1	138,047,390	138,064,281	0.06	LG-L
1	138,915,404	139,157,741	0.04	LG-L
1	139,633,324	139,653,373	0.21	LG-U
1	140,802,417	140,879,180	0.66	BW-U
1	140,838,046	141,243,417	0.24	LG-U
1	142,056,888	142,115,098	0.04	LG-L
1	142,067,867	142,099,589	0.08	BW-L
1	143,202,830	143,223,922	0.06	LG-L
1	143,665,916	143,697,509	0.54	BW-U
1	143,783,915	143,806,883	0.08	BW-L
1	143,935,535	143,966,940	0.22	LG-U
1	144,529,094	144,554,294	0.58	BW-U
1	144,533,253	144,554,294	0.20	LG-U
1	145,124,081	145,140,078	0.51	BW-U
1	145,379,101	145,529,509	0.23	LG-U
1	146,956,898	147,263,307	0.66	BW-U
1	146,996,868	147,029,999	0.20	LG-U
1	147,981,974	148,661,701	0.82	BW-U
1	148,499,786	148,651,466	0.26	LG-U
1	148,833,787	148,921,664	0.04	LG-L
1	148,833,787	149,016,095	0.00	BW-L
1	149,726,669	152,521,639	0.93	BW-U
1	150,244,477	150,720,557	0.24	LG-U
1	150,470,729	150,657,508	0.01	BW-L
1	151,297,152	151,425,445	0.07	BW-L
1	151,426,019	152,178,438	0.27	LG-U
1	152,078,774	152,102,965	0.04	BW-L
1	154,200,791	155,038,303	0.54	BW-U
1	155,379,431	155,409,680	0.20	LG-U
1	156,506,544	156,555,944	0.62	BW-U
1	156,721,032	157,385,675	0.05	LG-L
1	158,621,198	158,828,625	0.05	LG-L
1	159,121,548	159,263,728	0.24	LG-U
1	159,121,630	159,263,621	0.57	BW-U
1	159,438,530	159,642,844	0.05	LG-L
1	160,207,996	160,785,436	0.22	LG-U

1	160,244,586	160,287,743	0.07	BW-L
1	160,508,332	160,797,848	0.66	BW-U
1	161,064,433	161,288,744	0.05	LG-L
1	161,373,573	161,535,064	0.05	BW-L
1	161,546,520	161,673,467	0.23	LG-U
1	161,804,134	161,970,556	0.62	BW-U
1	162,478,305	163,030,434	0.05	LG-L
1	162,836,386	163,076,017	0.04	BW-L
1	162,878,131	163,491,021	0.22	LG-U
1	163,588,943	163,611,545	0.08	BW-L
1	163,703,238	163,731,497	0.52	BW-U
1	164,366,196	164,570,010	0.63	BW-U
1	164,526,277	164,557,150	0.22	LG-U
1	164,721,098	164,770,827	0.06	BW-L
1	164,735,397	164,770,827	0.05	LG-L
1	165,063,784	165,660,760	0.26	LG-U
1	165,303,792	165,596,484	0.68	BW-U
1	167,086,957	168,498,566	0.60	BW-U
1	167,555,085	168,480,857	0.21	LG-U
1	169,331,679	169,732,397	0.07	BW-L
1	169,382,563	169,419,583	0.03	LG-L
1	170,343,216	170,372,561	0.20	LG-U
1	170,359,901	170,372,561	0.51	BW-U
1	170,915,076	170,968,352	0.06	BW-L
1	171,872,899	171,893,375	0.06	LG-L
1	172,024,534	172,046,034	0.55	BW-U
1	173,597,516	173,941,361	0.24	LG-U
1	174,729,918	174,915,512	0.55	BW-U
1	174,932,994	174,956,295	0.07	BW-L
1	174,937,236	174,968,586	0.05	LG-L
1	175,903,675	176,089,183	0.57	BW-U
1	176,834,618	177,693,121	0.23	LG-U
1	176,837,169	177,693,121	0.58	BW-U
1	178,327,931	178,734,654	0.22	LG-U
1	179,551,353	179,640,872	0.21	LG-U
1	179,681,285	179,752,003	0.52	BW-U
1	182,261,557	182,278,402	0.20	LG-U
1	182,832,567	182,880,949	0.04	LG-L
1	182,853,376	182,876,578	0.06	BW-L
1	183,519,042	183,534,830	0.52	BW-U
1	183,529,755	183,698,841	0.22	LG-U
1	184,467,724	184,626,315	0.23	LG-U
1	185,196,591	185,241,578	0.26	LG-U

1	185,196,591	185,416,841	0.74	BW-U
1	186,949,522	186,963,105	0.06	LG-L
1	188,269,246	188,284,541	0.51	BW-U
1	188,663,729	188,756,359	0.04	LG-L
1	190,666,686	191,173,971	0.24	LG-U
1	190,714,558	190,747,189	0.05	BW-L
1	191,637,275	191,975,267	0.71	BW-U
1	191,953,611	191,966,120	0.20	LG-U
1	192,490,938	192,887,414	0.59	BW-U
1	193,339,654	193,359,674	0.20	LG-U
1	193,645,473	193,665,557	0.52	BW-U
1	193,939,943	193,960,555	0.07	BW-L
1	194,211,322	194,227,126	0.20	LG-U
2	1,254,719	1,276,409	0.06	LG-L
2	3,711,786	3,741,245	0.05	LG-L
2	4,716,998	4,740,715	0.06	LG-L
2	5,410,332	5,444,821	0.56	BW-U
2	6,442,214	6,477,449	0.05	LG-L
2	7,523,577	7,551,619	0.05	LG-L
2	8,724,225	8,747,572	0.20	LG-U
2	8,909,787	8,932,064	0.06	LG-L
2	11,992,949	12,514,619	0.03	LG-L
2	12,108,802	12,249,637	0.02	BW-L
2	13,320,934	13,987,558	0.23	LG-U
2	13,754,019	14,009,494	0.60	BW-U
2	14,607,509	14,976,241	0.21	LG-U
2	14,666,030	14,712,311	0.60	BW-U
2	15,478,956	15,516,089	0.20	LG-U
2	18,715,224	18,739,474	0.56	BW-U
2	18,935,659	20,006,860	0.26	LG-U
2	19,971,618	20,016,589	0.58	BW-U
2	21,521,930	21,716,202	0.53	BW-U
2	21,546,414	21,655,717	0.06	LG-L
2	22,478,520	22,496,660	0.06	LG-L
2	24,158,283	24,465,030	0.52	BW-U
2	26,087,115	26,490,314	0.25	LG-U
2	27,604,583	27,639,070	0.05	LG-L
2	27,609,876	27,630,160	0.08	BW-L
2	29,710,072	29,732,270	0.51	BW-U
2	30,259,123	30,317,199	0.05	LG-L
2	30,278,951	30,329,517	0.06	BW-L
2	30,602,381	30,636,767	0.53	BW-U
2	31,288,760	31,313,579	0.20	LG-U

2	31,621,119	31,638,763	0.06	LG-L
2	31,805,792	31,829,568	0.07	BW-L
2	32,442,546	32,478,619	0.20	LG-U
2	33,670,257	33,698,142	0.06	LG-L
2	34,041,705	34,081,309	0.06	BW-L
2	34,840,663	34,880,005	0.05	LG-L
2	36,867,618	36,900,900	0.06	LG-L
2	37,503,545	37,541,400	0.23	LG-U
2	38,768,253	38,805,734	0.05	LG-L
2	41,023,868	41,360,951	0.25	LG-U
2	43,957,154	43,997,435	0.06	BW-L
2	45,285,004	45,339,358	0.21	LG-U
2	45,299,810	45,697,314	0.54	BW-U
2	46,209,647	46,257,723	0.22	LG-U
2	46,665,504	46,701,610	0.56	BW-U
2	48,300,308	48,356,680	0.59	BW-U
2	50,069,573	50,249,428	0.04	LG-L
2	50,454,845	50,793,336	0.75	BW-U
2	50,509,204	50,612,925	0.21	LG-U
2	56,601,004	56,900,904	0.05	LG-L
2	56,870,413	56,899,952	0.07	BW-L
2	57,851,345	57,919,930	0.63	BW-U
2	60,282,670	60,315,907	0.07	BW-L
2	60,448,058	60,477,058	0.06	LG-L
2	62,219,445	62,250,695	0.06	BW-L
2	62,610,833	62,637,583	0.53	BW-U
2	64,002,393	64,061,503	0.05	LG-L
2	66,443,035	66,471,338	0.51	BW-U
2	66,579,950	66,618,759	0.20	LG-U
2	67,525,380	67,813,699	0.54	BW-U
2	67,874,326	67,904,798	0.19	LG-U
2	69,167,813	69,200,233	0.06	LG-L
2	69,985,562	70,028,537	0.51	BW-U
2	71,422,416	71,470,960	0.21	LG-U
2	72,367,663	72,821,517	0.25	LG-U
2	72,465,649	72,518,498	0.03	BW-L
2	72,466,233	72,523,991	0.03	LG-L
2	73,170,359	73,767,252	0.06	BW-L
2	73,177,164	73,982,655	0.04	LG-L
2	74,247,580	74,463,169	0.63	BW-U
2	75,822,569	75,846,801	0.51	BW-U
2	76,386,098	76,401,938	0.52	BW-U
2	78,017,562	78,047,660	0.05	LG-L

2	78,018,173	78,047,660	0.07	BW-L
2	78,308,220	78,539,035	0.61	BW-U
2	79,085,768	79,464,425	0.32	LG-U
2	79,120,897	79,150,192	0.07	BW-L
2	79,418,972	79,461,926	0.54	BW-U
2	80,471,939	80,722,131	0.63	BW-U
2	81,114,910	81,313,704	0.22	LG-U
2	81,356,139	81,564,456	0.59	BW-U
2	81,580,114	81,977,415	0.03	BW-L
2	81,586,109	82,057,139	0.04	LG-L
2	82,750,853	83,307,881	0.57	BW-U
2	82,750,853	83,477,791	0.23	LG-U
2	84,311,596	85,079,253	0.70	BW-U
2	84,611,314	84,644,824	0.21	LG-U
2	85,931,314	86,583,670	0.77	BW-U
2	86,158,318	86,703,786	0.25	LG-U
2	87,519,967	87,736,417	0.58	BW-U
2	88,472,359	88,579,747	0.22	LG-U
2	88,782,897	88,805,513	0.07	BW-L
2	89,500,095	89,570,026	0.26	LG-U
2	90,766,561	90,898,916	0.24	LG-U
2	90,839,128	91,342,577	0.05	BW-L
2	91,370,665	91,387,775	0.53	BW-U
2	92,273,841	92,601,350	0.05	LG-L
2	92,287,279	92,481,014	0.06	BW-L
2	93,682,562	94,402,094	0.05	BW-L
2	95,529,261	95,562,224	0.08	BW-L
2	95,887,405	95,915,287	0.20	LG-U
2	96,072,344	96,091,899	0.07	BW-L
2	96,575,182	96,612,566	0.54	BW-U
2	97,135,288	97,163,857	0.55	BW-U
2	97,753,982	97,778,921	0.07	BW-L
2	98,542,158	98,561,499	0.06	LG-L
2	102,665,193	103,192,592	0.56	BW-U
2	102,683,568	102,725,495	0.22	LG-U
2	103,213,369	103,232,528	0.06	BW-L
2	104,577,404	105,101,612	0.04	LG-L
2	104,832,305	104,851,062	0.08	BW-L
2	106,233,586	106,271,908	0.05	LG-L
2	107,830,556	107,866,167	0.03	BW-L
2	107,851,085	107,868,688	0.06	LG-L
2	107,951,402	108,324,721	0.55	BW-U
2	108,122,366	108,145,782	0.21	LG-U

2	109,901,344	109,961,290	0.22	LG-U
2	109,902,343	110,067,338	0.61	BW-U
2	110,287,910	110,314,410	0.06	LG-L
2	110,792,545	111,127,249	0.05	BW-L
2	110,874,286	110,898,112	0.53	BW-U
2	111,779,831	112,711,412	0.04	LG-L
2	111,780,792	111,810,338	0.08	BW-L
2	112,626,661	113,774,085	0.05	BW-L
2	113,517,867	114,461,273	0.05	LG-L
2	114,319,853	114,341,565	0.07	BW-L
2	117,335,609	117,356,505	0.52	BW-U
2	117,336,110	117,356,505	0.20	LG-U
2	117,908,284	118,064,259	0.05	LG-L
2	119,121,036	119,294,819	0.05	LG-L
2	119,121,036	119,780,209	0.05	BW-L
2	121,448,317	122,041,521	0.05	BW-L
2	121,594,944	121,619,256	0.55	BW-U
2	121,703,341	122,013,676	0.04	LG-L
2	122,369,947	122,408,083	0.58	BW-U
2	122,976,602	122,998,281	0.20	LG-U
2	122,976,602	123,250,117	0.61	BW-U
2	123,556,204	123,595,552	0.20	LG-U
2	123,710,819	123,731,509	0.05	LG-L
2	124,070,145	124,340,500	0.53	BW-U
2	125,285,159	125,320,460	0.59	BW-U
2	125,844,447	126,114,969	0.06	BW-L
2	126,678,367	126,737,768	0.06	LG-L
2	127,154,944	127,175,399	0.52	BW-U
2	127,528,038	127,559,402	0.05	LG-L
2	128,050,732	128,076,199	0.54	BW-U
2	128,624,861	128,657,714	0.05	LG-L
2	128,741,868	128,910,160	0.04	BW-L
2	129,617,573	129,958,082	0.23	LG-U
2	129,992,294	130,174,198	0.05	BW-L
2	130,764,762	132,364,324	0.04	BW-L
2	131,107,254	133,523,335	0.03	LG-L
2	131,256,281	131,275,561	0.20	LG-U
2	133,356,866	133,465,145	0.07	BW-L
2	135,496,322	136,214,962	0.21	LG-U
2	136,917,214	136,966,955	0.58	BW-U
2	136,917,214	136,970,865	0.22	LG-U
2	138,469,076	138,494,620	0.53	BW-U
2	139,375,063	139,405,258	0.05	LG-L

2	140,269,082	140,769,481	0.01	BW-L
2	140,271,526	141,083,414	0.03	LG-L
2	142,669,542	142,731,742	0.04	BW-L
2	143,476,382	143,496,648	0.51	BW-U
2	144,327,359	144,374,999	0.05	LG-L
2	145,588,698	146,300,633	0.01	BW-L
2	145,826,510	145,882,701	0.66	BW-U
2	145,987,205	146,319,664	0.03	LG-L
2	146,470,566	146,490,399	0.20	LG-U
2	146,949,180	146,970,068	0.53	BW-U
3	320,263	408,990	0.05	LG-L
3	1,419,336	1,479,746	0.06	BW-L
3	1,767,560	1,793,471	0.06	LG-L
3	4,633,728	4,658,160	0.20	LG-U
3	5,773,758	6,061,872	0.27	LG-U
3	6,167,726	7,269,029	0.02	BW-L
3	6,431,257	6,690,595	0.04	LG-L
3	6,628,883	7,276,820	0.26	LG-U
3	8,281,426	8,306,342	0.52	BW-U
3	9,506,957	9,542,304	0.56	BW-U
3	9,511,413	9,543,859	0.20	LG-U
3	9,688,855	9,705,889	0.06	LG-L
3	10,282,400	11,786,261	0.04	LG-L
3	10,629,503	11,229,324	0.06	BW-L
3	12,152,722	12,196,380	0.08	BW-L
3	12,276,499	12,705,631	0.58	BW-U
3	12,376,900	12,735,712	0.20	LG-U
3	12,847,344	13,285,255	0.07	BW-L
3	13,356,427	13,467,255	0.63	BW-U
3	13,806,554	13,827,135	0.08	BW-L
3	14,698,048	14,735,257	0.22	LG-U
3	16,494,431	16,527,235	0.05	BW-L
3	18,502,320	18,555,412	0.05	LG-L
3	19,372,889	19,410,526	0.07	BW-L
3	20,124,055	20,336,700	0.05	BW-L
3	20,378,393	20,530,346	0.24	LG-U
3	20,418,372	20,615,278	0.58	BW-U
3	21,501,813	21,705,493	0.25	LG-U
3	21,596,816	22,095,628	0.07	BW-L
3	21,665,666	21,707,951	0.58	BW-U
3	22,000,709	22,098,744	0.06	LG-L
3	22,598,789	22,950,067	0.05	BW-L
3	22,890,062	23,044,823	0.05	LG-L

3	23,794,815	23,846,394	0.03	BW-L
3	24,829,946	24,852,498	0.07	BW-L
3	26,271,660	26,845,328	0.23	LG-U
3	26,362,754	26,410,608	0.02	BW-L
3	27,772,603	29,609,673	0.72	BW-U
3	27,920,939	28,849,114	0.27	LG-U
3	28,399,719	28,596,692	0.02	BW-L
3	29,491,809	29,660,397	0.21	LG-U
3	31,713,919	31,757,322	0.04	LG-L
3	31,720,264	31,792,913	0.07	BW-L
3	32,613,450	32,682,843	0.56	BW-U
3	32,655,959	32,682,843	0.20	LG-U
3	33,348,669	34,913,481	0.03	BW-L
3	34,196,557	34,585,808	0.20	LG-U
3	36,125,173	36,244,850	0.52	BW-U
3	36,543,375	36,595,659	0.05	LG-L
3	36,543,375	36,627,502	0.05	BW-L
3	36,629,200	36,823,879	0.21	LG-U
3	37,155,460	37,199,596	0.06	BW-L
3	38,009,595	38,054,389	0.61	BW-U
3	38,031,719	38,054,389	0.20	LG-U
3	41,032,855	43,152,536	0.67	BW-U
3	41,445,965	43,089,377	0.27	LG-U
3	43,343,364	43,364,668	0.07	BW-L
3	44,029,977	47,579,337	0.75	BW-U
3	44,186,023	44,204,943	0.20	LG-U
3	45,473,042	46,772,410	0.22	LG-U
3	45,643,050	46,171,577	0.06	BW-L
3	47,418,567	47,465,221	0.22	LG-U
3	48,295,962	48,969,283	0.76	BW-U
3	48,302,524	48,931,846	0.22	LG-U
3	48,532,979	49,351,750	0.06	BW-L
3	48,601,630	48,849,147	0.06	LG-L
3	49,956,133	50,716,516	0.73	BW-U
3	50,678,529	50,848,504	0.24	LG-U
3	50,701,546	50,785,517	0.02	BW-L
3	51,774,255	52,054,611	0.63	BW-U
3	52,424,633	52,932,282	0.04	LG-L
3	53,143,832	53,180,537	0.06	BW-L
3	53,224,543	53,253,795	0.51	BW-U
3	54,466,648	54,488,554	0.55	BW-U
3	54,577,913	55,003,855	0.05	LG-L
3	55,088,887	55,141,005	0.58	BW-U

3	55,904,835	55,938,261	0.05	LG-L
3	58,034,427	58,066,089	0.53	BW-U
3	58,613,792	58,637,720	0.06	LG-L
3	59,122,973	59,146,974	0.59	BW-U
3	59,202,622	59,231,427	0.07	BW-L
3	60,356,031	61,197,748	0.04	LG-L
3	60,944,428	61,132,698	0.03	BW-L
3	61,837,810	61,851,374	0.06	LG-L
3	63,228,866	64,770,658	0.67	BW-U
3	64,499,044	64,519,068	0.08	BW-L
3	65,044,935	65,076,224	0.05	LG-L
3	65,366,745	66,340,427	0.58	BW-U
3	67,753,710	67,782,451	0.57	BW-U
3	67,882,844	67,905,960	0.06	LG-L
3	68,620,899	68,658,164	0.08	BW-L
3	69,297,198	69,336,178	0.05	LG-L
3	69,945,584	69,986,940	0.67	BW-U
3	71,133,624	71,247,607	0.07	BW-L
3	72,265,806	72,288,043	0.07	BW-L
3	72,574,767	72,680,359	0.04	LG-L
3	73,115,920	73,569,141	0.24	LG-U
3	73,119,566	74,312,534	0.67	BW-U
3	73,260,405	73,292,590	0.04	LG-L
3	75,815,930	75,895,205	0.20	LG-U
3	76,027,460	76,063,825	0.07	BW-L
3	76,395,628	76,415,945	0.52	BW-U
3	76,802,258	76,827,026	0.20	LG-U
3	77,178,927	78,495,024	0.05	BW-L
3	77,799,255	77,832,231	0.21	LG-U
3	78,333,886	78,357,498	0.22	LG-U
3	79,115,925	79,151,303	0.55	BW-U
3	79,248,829	79,275,469	0.20	LG-U
3	79,685,980	79,713,858	0.06	BW-L
3	79,792,211	80,815,179	0.67	BW-U
3	79,973,088	80,502,888	0.21	LG-U
3	80,912,375	80,965,592	0.07	BW-L
3	80,919,014	80,943,200	0.05	LG-L
3	81,500,811	81,773,761	0.58	BW-U
3	81,558,990	81,587,532	0.19	LG-U
3	81,708,092	81,744,267	0.05	LG-L
3	82,103,968	82,134,246	0.07	BW-L
3	82,984,757	83,608,963	0.04	LG-L
3	84,003,744	84,479,736	0.57	BW-U

3	84,578,864	84,930,135	0.21	LG-U
3	84,902,600	84,966,481	0.05	BW-L
3	85,177,879	85,200,516	0.05	LG-L
3	85,554,148	85,586,798	0.07	BW-L
3	85,853,325	86,130,933	0.54	BW-U
3	86,536,016	86,944,863	0.23	LG-U
3	86,761,698	86,790,479	0.06	LG-L
3	87,619,886	87,650,101	0.20	LG-U
3	87,721,694	87,742,746	0.04	BW-L
3	88,313,311	88,333,452	0.07	BW-L
3	88,727,765	89,279,185	0.23	LG-U
3	88,931,225	88,946,473	0.07	BW-L
3	89,563,722	89,588,589	0.07	BW-L
3	89,727,787	89,750,187	0.06	LG-L
3	92,507,612	92,799,877	0.06	BW-L
3	92,524,510	93,095,228	0.04	LG-L
3	93,599,395	93,631,663	0.60	BW-U
3	93,601,314	93,624,740	0.21	LG-U
3	93,921,077	94,497,880	0.04	LG-L
3	94,433,991	94,453,489	0.07	BW-L
3	95,480,561	95,921,645	0.04	BW-L
3	95,616,557	95,654,119	0.20	LG-U
3	96,290,451	99,300,060	0.27	LG-U
3	96,547,005	98,832,005	0.72	BW-U
3	97,872,862	97,899,427	0.06	BW-L
3	99,459,637	99,484,526	0.08	BW-L
3	99,631,797	99,814,933	0.55	BW-U
3	100,982,436	100,995,415	0.07	BW-L
3	101,078,725	101,101,721	0.05	LG-L
3	101,730,331	102,011,543	0.04	BW-L
3	102,180,647	102,291,969	0.56	BW-U
3	102,235,429	102,822,585	0.27	LG-U
3	102,997,178	103,072,137	0.06	BW-L
3	104,461,540	104,493,074	0.21	LG-U
3	104,471,603	104,492,194	0.54	BW-U
3	109,365,580	109,395,871	0.05	LG-L
3	109,421,486	109,641,166	0.22	LG-U
4	251,064	300,288	0.07	BW-L
4	1,431,666	1,468,407	0.06	LG-L
4	3,132,604	3,186,108	0.03	LG-L
4	3,138,757	3,178,869	0.06	BW-L
4	3,853,836	3,886,019	0.06	LG-L
4	5,919,600	6,312,315	0.04	LG-L

4	5,974,587	6,004,044	0.07	BW-L
4	7,322,025	8,185,538	0.04	LG-L
4	9,136,756	9,170,523	0.05	LG-L
4	9,723,404	9,734,720	0.05	LG-L
4	9,946,469	10,583,512	0.22	LG-U
4	10,648,974	10,669,448	0.08	BW-L
4	13,054,965	13,082,572	0.06	LG-L
4	14,823,079	14,924,496	0.06	BW-L
4	14,976,792	15,637,478	0.04	LG-L
4	17,230,977	17,276,084	0.21	LG-U
4	17,867,315	17,938,216	0.24	LG-U
4	19,964,489	20,020,853	0.06	BW-L
4	20,708,869	20,828,812	0.05	LG-L
4	21,699,121	21,738,250	0.66	BW-U
4	21,905,221	21,936,770	0.05	LG-L
4	21,909,619	21,932,980	0.08	BW-L
4	22,820,765	22,846,729	0.20	LG-U
4	23,026,986	23,056,097	0.52	BW-U
4	25,176,714	25,282,754	0.58	BW-U
4	25,264,974	25,281,744	0.20	LG-U
4	25,782,537	25,977,294	0.05	LG-L
4	25,785,327	25,811,048	0.07	BW-L
4	26,512,347	26,545,215	0.51	BW-U
4	26,562,444	26,602,149	0.08	BW-L
4	26,691,151	26,738,311	0.20	LG-U
4	27,240,672	28,350,137	0.69	BW-U
4	31,808,891	32,227,764	0.80	BW-U
4	31,809,848	31,861,929	0.22	LG-U
4	32,577,588	32,615,957	0.05	LG-L
4	32,594,714	32,614,088	0.08	BW-L
4	33,236,851	33,273,246	0.05	LG-L
4	34,245,647	35,193,981	0.30	LG-U
4	36,251,637	36,343,275	0.23	LG-U
4	37,846,065	38,332,191	0.23	LG-U
4	38,508,826	39,422,947	0.01	BW-L
4	38,893,048	38,939,012	0.21	LG-U
4	40,467,287	40,499,788	0.04	LG-L
4	40,700,770	40,898,039	0.05	BW-L
4	40,814,799	40,869,822	0.24	LG-U
4	41,897,670	41,938,275	0.07	BW-L
4	42,076,152	42,252,792	0.22	LG-U
4	42,630,670	42,656,241	0.05	LG-L
4	43,350,537	43,397,242	0.05	LG-L

4	44,364,260	44,724,945	0.06	BW-L
4	46,163,339	46,198,136	0.22	LG-U
4	46,200,538	46,231,765	0.55	BW-U
4	47,671,719	47,707,281	0.05	LG-L
4	47,813,480	47,841,319	0.07	BW-L
4	49,729,505	50,943,820	0.70	BW-U
4	49,729,505	50,943,820	0.70	BW-U
4	49,979,208	50,010,939	0.21	LG-U
4	50,874,442	50,892,459	0.08	BW-L
4	51,594,973	52,458,269	0.06	BW-L
4	52,715,572	52,741,001	0.22	LG-U
4	53,387,763	53,873,864	0.20	LG-U
4	53,848,765	53,876,951	0.54	BW-U
4	54,218,710	54,244,359	0.05	LG-L
4	55,493,308	56,078,938	0.55	BW-U
4	56,465,637	56,553,996	0.00	BW-L
4	56,467,464	56,548,160	0.05	LG-L
4	56,714,160	56,736,952	0.20	LG-U
4	56,910,019	56,934,269	0.55	BW-U
4	57,961,333	57,983,149	0.07	BW-L
4	58,349,752	58,373,173	0.53	BW-U
4	59,771,750	59,805,562	0.05	LG-L
4	59,940,444	59,990,055	0.62	BW-U
4	59,947,628	59,982,597	0.22	LG-U
4	61,257,404	61,685,210	0.26	LG-U
4	62,344,704	62,362,262	0.52	BW-U
4	62,818,505	62,848,126	0.07	BW-L
4	62,819,755	62,851,795	0.05	LG-L
4	62,974,871	63,530,431	0.23	LG-U
4	64,558,175	64,576,699	0.06	LG-L
4	67,484,728	67,506,941	0.52	BW-U
4	68,682,964	68,742,420	0.23	LG-U
4	69,483,695	70,121,893	0.21	LG-U
4	69,660,565	70,124,312	0.54	BW-U
4	70,867,475	71,167,597	0.51	BW-U
4	70,956,333	71,175,698	0.21	LG-U
4	71,674,214	71,713,358	0.08	BW-L
4	71,919,395	72,407,398	0.23	LG-U
4	73,856,156	74,026,737	0.05	LG-L
4	74,345,049	74,387,805	0.64	BW-U
4	74,350,139	74,383,364	0.21	LG-U
4	74,587,540	75,568,422	0.03	LG-L
4	74,592,140	74,830,946	0.07	BW-L

4	75,435,907	75,492,233	0.73	BW-U
4	75,449,277	75,485,022	0.20	LG-U
4	76,684,890	77,151,194	0.06	BW-L
4	76,686,061	77,265,701	0.05	LG-L
4	81,079,351	81,101,682	0.05	LG-L
4	81,082,319	81,098,184	0.08	BW-L
4	81,321,604	82,505,144	0.58	BW-U
4	81,686,131	82,106,927	0.23	LG-U
4	82,817,430	82,863,103	0.03	BW-L
4	83,050,675	83,075,182	0.21	LG-U
4	84,986,909	85,014,470	0.06	LG-L
4	86,262,494	87,797,665	0.05	LG-L
4	86,748,955	86,901,424	0.55	BW-U
4	87,187,378	87,253,655	0.05	BW-L
4	89,120,494	89,190,965	0.21	LG-U
4	90,024,429	90,050,030	0.22	LG-U
5	510,501	1,780,710	0.05	LG-L
5	977,926	1,274,626	0.03	BW-L
5	4,246,343	4,339,249	0.02	BW-L
5	4,967,401	5,164,439	0.05	LG-L
5	6,802,661	6,841,097	0.22	LG-U
5	6,802,661	6,841,097	0.22	LG-U
5	7,551,596	7,567,858	0.20	LG-U
5	8,325,895	8,410,436	0.23	LG-U
5	8,918,803	8,940,570	0.20	LG-U
5	10,058,494	10,121,578	0.63	BW-U
5	11,990,130	12,329,589	0.23	LG-U
5	12,054,794	12,330,246	0.76	BW-U
5	13,145,143	13,500,321	0.54	BW-U
5	13,532,597	13,557,210	0.06	LG-L
5	14,001,319	14,018,826	0.08	BW-L
5	14,232,688	14,810,989	0.04	LG-L
5	14,657,174	14,673,513	0.08	BW-L
5	14,851,575	14,893,568	0.20	LG-U
5	15,486,412	15,510,497	0.06	LG-L
5	16,586,010	16,640,239	0.04	LG-L
5	17,449,400	17,789,282	0.55	BW-U
5	18,025,516	18,551,749	0.05	LG-L
5	18,262,172	18,289,211	0.07	BW-L
5	19,185,511	19,204,485	0.06	LG-L
5	19,438,004	19,468,098	0.08	BW-L
5	19,963,061	20,029,935	0.05	LG-L
5	20,365,923	21,052,848	0.06	BW-L

5	20,521,976	20,540,585	0.20	LG-U
5	20,680,566	20,883,656	0.05	LG-L
5	21,867,890	22,049,811	0.02	BW-L
5	21,933,423	22,251,646	0.02	LG-L
5	22,035,576	22,110,430	0.24	LG-U
5	22,825,255	23,061,317	0.22	LG-U
5	22,863,032	23,388,841	0.55	BW-U
5	22,863,032	23,388,841	0.55	BW-U
5	23,956,157	24,113,787	0.63	BW-U
5	23,971,310	25,010,763	0.26	LG-U
5	24,633,095	25,026,209	0.65	BW-U
5	25,499,392	25,519,779	0.06	LG-L
5	26,544,477	26,582,087	0.22	LG-U
5	26,544,477	26,643,893	0.58	BW-U
5	28,981,027	29,008,480	0.20	LG-U
5	29,051,196	29,071,915	0.07	BW-L
5	30,342,781	30,371,431	0.53	BW-U
5	30,559,879	31,928,539	0.04	LG-L
5	31,465,871	32,288,833	0.03	BW-L
5	33,103,817	33,135,162	0.52	BW-U
5	33,103,817	33,137,219	0.20	LG-U
5	34,352,062	35,330,953	0.68	BW-U
5	35,900,814	36,136,217	0.08	BW-L
5	36,166,430	37,060,516	0.22	LG-U
5	38,521,742	38,671,770	0.57	BW-U
5	39,289,649	40,264,973	0.63	BW-U
5	39,348,630	39,365,437	0.19	LG-U
5	39,450,519	39,475,433	0.05	LG-L
5	39,791,767	40,314,508	0.04	BW-L
5	40,074,894	40,450,659	0.04	LG-L
5	40,957,210	41,088,264	0.73	BW-U
5	41,015,998	41,066,763	0.21	LG-U
5	41,098,078	41,286,369	0.04	BW-L
5	41,244,628	41,292,983	0.05	LG-L
5	42,291,548	42,344,999	0.61	BW-U
5	42,304,673	42,576,203	0.20	LG-U
5	43,503,476	43,609,159	0.60	BW-U
5	43,568,781	43,876,059	0.22	LG-U
5	44,831,553	44,854,526	0.53	BW-U
5	45,376,451	45,404,204	0.05	LG-L
5	45,806,505	46,022,300	0.28	LG-U
5	46,907,007	46,938,010	0.06	BW-L
5	47,624,314	48,957,772	0.28	LG-U

5	48,067,550	48,776,793	0.60	BW-U
5	48,322,820	48,412,904	0.01	BW-L
5	49,627,505	49,645,376	0.53	BW-U
5	50,030,349	50,049,229	0.05	LG-L
5	51,621,719	51,673,026	0.05	LG-L
5	51,651,272	51,796,847	0.07	BW-L
5	52,843,645	52,866,444	0.07	BW-L
5	53,673,093	54,159,282	0.60	BW-U
5	53,682,807	54,061,913	0.22	LG-U
5	55,737,005	55,752,795	0.51	BW-U
5	57,638,705	57,652,541	0.54	BW-U
5	58,260,347	58,623,160	0.62	BW-U
6	506,960	521,203	0.06	LG-L
6	1,316,629	1,346,842	0.56	BW-U
6	2,718,275	2,740,956	0.20	LG-U
6	4,436,055	4,453,111	0.05	LG-L
6	5,608,614	5,638,696	0.56	BW-U
6	6,273,221	7,053,477	0.04	LG-L
6	6,736,516	6,755,866	0.08	BW-L
6	8,147,142	8,166,877	0.52	BW-U
6	8,616,953	8,653,362	0.06	BW-L
6	8,624,588	8,653,362	0.25	LG-U
6	10,845,832	10,864,118	0.51	BW-U
6	11,135,930	11,194,180	0.02	BW-L
6	11,155,546	11,194,180	0.04	LG-L
6	11,426,283	11,718,376	0.63	BW-U
6	11,556,814	11,715,668	0.22	LG-U
6	12,216,065	12,235,766	0.06	LG-L
6	12,474,480	12,898,970	0.07	BW-L
6	12,873,816	14,033,922	0.04	LG-L
6	13,178,679	13,198,091	0.55	BW-U
6	14,232,482	14,246,652	0.52	BW-U
6	15,244,342	16,230,630	0.04	LG-L
6	15,563,024	16,185,004	0.03	BW-L
6	16,954,997	16,992,291	0.04	BW-L
6	17,341,918	17,364,264	0.21	LG-U
6	17,896,885	18,093,687	0.01	BW-L
6	17,932,192	18,094,567	0.03	LG-L
6	18,820,119	18,834,336	0.51	BW-U
6	18,848,796	18,876,817	0.22	LG-U
6	18,900,349	18,931,393	0.07	BW-L
6	19,447,361	19,468,416	0.56	BW-U
6	20,550,571	20,566,437	0.07	BW-L

6	20,575,076	21,224,325	0.61	BW-U
6	20,758,401	21,532,271	0.27	LG-U
6	23,699,235	23,735,878	0.07	BW-L
6	24,122,542	24,336,907	0.04	LG-L
6	24,250,606	24,265,607	0.08	BW-L
6	25,761,112	26,129,140	0.05	LG-L
6	27,101,675	27,131,959	0.20	LG-U
6	30,123,982	30,158,636	0.21	LG-U
6	31,071,229	31,790,172	0.25	LG-U
6	31,752,445	31,790,172	0.65	BW-U
6	33,112,007	33,197,745	0.05	LG-L
7	284,853	774,594	0.03	LG-L
7	408,479	770,100	0.04	BW-L
7	1,851,523	2,413,069	0.06	BW-L
7	2,245,091	3,367,606	0.04	LG-L
7	3,286,513	3,307,527	0.52	BW-U
7	3,867,172	3,896,527	0.07	BW-L
7	4,144,081	4,605,926	0.05	LG-L
7	4,697,775	4,723,416	0.54	BW-U
7	5,493,807	5,810,496	0.21	LG-U
7	5,771,768	5,875,632	0.74	BW-U
7	6,558,584	6,583,082	0.05	LG-L
7	7,753,711	7,892,864	0.06	BW-L
7	8,452,184	8,603,603	0.04	LG-L
7	8,577,923	8,603,603	0.06	BW-L
7	9,276,545	9,300,834	0.07	BW-L
7	9,923,396	9,958,693	0.05	BW-L
7	10,874,012	11,220,202	0.20	LG-U
7	11,106,507	11,171,947	0.07	BW-L
7	12,072,361	12,090,467	0.05	LG-L
7	13,147,924	14,456,327	0.05	LG-L
7	13,887,433	13,919,939	0.67	BW-U
7	14,448,385	14,468,635	0.06	BW-L
7	16,311,552	16,332,231	0.21	LG-U
7	16,404,488	16,550,038	0.62	BW-U
7	17,029,531	17,050,182	0.06	BW-L
7	17,131,374	17,301,699	0.64	BW-U
7	17,251,168	17,301,672	0.22	LG-U
7	17,886,824	17,949,237	0.55	BW-U
7	17,987,106	18,637,425	0.20	LG-U
7	18,617,357	18,808,978	0.65	BW-U
7	19,704,808	19,919,070	0.05	LG-L
7	21,253,136	21,272,259	0.05	BW-L

7	21,268,839	21,335,902	0.59	BW-U
7	23,290,513	23,347,145	0.04	LG-L
7	23,302,539	23,337,011	0.07	BW-L
7	24,185,661	24,499,912	0.04	LG-L
7	24,188,665	25,244,175	0.03	BW-L
7	24,794,665	24,822,035	0.21	LG-U
7	25,218,423	25,239,613	0.05	LG-L
7	25,465,604	25,531,175	0.60	BW-U
7	26,063,650	26,106,105	0.05	LG-L
7	26,604,752	27,008,916	0.62	BW-U
7	26,689,636	26,726,268	0.21	LG-U
7	27,617,034	27,638,269	0.08	BW-L
7	28,360,752	28,880,095	0.05	LG-L
7	28,750,099	28,883,268	0.06	BW-L
7	29,015,530	29,064,866	0.53	BW-U
7	30,313,241	30,668,289	0.54	BW-U
7	30,519,273	30,668,289	0.21	LG-U
7	31,441,026	31,585,502	0.05	LG-L
7	31,717,186	31,737,269	0.55	BW-U
7	31,718,114	31,735,504	0.20	LG-U
7	32,482,607	32,507,176	0.05	LG-L
7	33,481,910	33,500,769	0.06	LG-L
7	35,133,240	35,323,399	0.53	BW-U
7	35,838,428	35,868,104	0.55	BW-U
8	281,789	1,132,843	0.80	BW-U
8	349,501	2,042,862	0.25	LG-U
8	2,110,522	2,217,296	0.53	BW-U
8	3,101,720	3,120,454	0.05	LG-L
8	3,957,847	4,391,577	0.20	LG-U
8	5,879,372	5,912,374	0.22	LG-U
8	5,883,583	5,922,717	0.64	BW-U
8	6,472,570	6,498,568	0.07	BW-L
8	7,052,741	7,503,592	0.05	LG-L
8	7,054,374	7,066,873	0.08	BW-L
8	8,204,654	8,563,104	0.04	LG-L
8	8,644,253	9,477,770	0.01	BW-L
8	8,760,964	9,516,304	0.29	LG-U
8	10,735,171	11,407,663	0.24	LG-U
8	11,325,168	12,171,174	0.05	BW-L
8	11,680,461	12,182,135	0.04	LG-L
8	12,952,124	12,997,959	0.07	BW-L
8	14,072,533	14,225,749	0.27	LG-U
8	14,567,533	14,587,407	0.53	BW-U

8	15,435,059	15,453,896	0.08	BW-L
8	17,265,656	17,297,539	0.06	LG-L
8	17,428,976	17,469,904	0.21	LG-U
8	17,443,889	17,471,534	0.53	BW-U
8	17,805,679	17,821,973	0.08	BW-L
8	18,089,557	18,935,397	0.05	LG-L
8	18,913,230	18,945,051	0.06	BW-L
8	20,551,778	20,567,168	0.06	LG-L
8	21,398,144	21,411,425	0.51	BW-U
8	22,507,666	22,522,489	0.08	BW-L
8	23,160,976	23,356,127	0.05	BW-L
8	23,197,307	23,829,662	0.03	LG-L
8	24,704,482	24,721,574	0.51	BW-U
8	25,929,349	25,966,042	0.04	BW-L
8	27,125,740	27,141,443	0.20	LG-U
8	27,365,262	27,406,966	0.06	BW-L
8	27,629,847	27,653,942	0.06	LG-L
9	1,055,233	1,125,406	0.21	LG-U
9	2,060,601	2,089,638	0.53	BW-U
9	3,926,355	3,942,073	0.05	LG-L
9	4,985,319	5,759,716	0.24	LG-U
9	5,201,413	5,690,758	0.73	BW-U
9	6,106,107	6,146,570	0.03	BW-L
9	6,320,435	6,764,558	0.22	LG-U
9	6,437,123	6,466,137	0.52	BW-U
9	7,671,979	7,684,530	0.08	BW-L
9	7,796,160	7,872,055	0.56	BW-U
9	9,528,481	9,888,387	0.04	LG-L
9	9,769,069	9,878,672	0.07	BW-L
9	11,054,877	11,092,713	0.08	BW-L
9	11,257,105	12,147,823	0.05	LG-L
9	11,806,336	12,031,014	0.02	BW-L
9	12,601,916	13,551,709	0.22	LG-U
9	12,921,550	13,277,653	0.63	BW-U
9	16,006,687	17,001,069	0.24	LG-U
9	18,116,590	18,498,695	0.19	LG-U
9	19,841,287	19,872,151	0.04	BW-L
9	19,845,629	19,922,704	0.05	LG-L
9	20,406,745	20,428,065	0.60	BW-U
9	20,410,071	20,422,153	0.20	LG-U
9	21,944,789	21,960,624	0.05	LG-L
9	22,818,301	22,831,521	0.05	LG-L
9	23,109,187	23,467,664	0.24	LG-U

9	23,184,081	23,524,498	0.78	BW-U
10	2,597,360	2,628,200	0.20	LG-U
10	3,116,398	3,129,335	0.51	BW-U
10	3,940,389	3,953,752	0.51	BW-U
10	3,958,924	3,969,539	0.20	LG-U
10	4,843,362	5,709,976	0.24	LG-U
10	5,030,766	5,153,695	0.67	BW-U
10	5,691,893	6,137,104	0.07	BW-L
10	6,502,500	7,022,210	0.67	BW-U
10	6,505,595	8,215,135	0.24	LG-U
10	10,299,507	10,319,243	0.20	LG-U
10	11,182,653	11,227,115	0.56	BW-U
10	11,624,103	12,109,182	0.03	BW-L
10	11,759,591	11,776,731	0.21	LG-U
10	12,573,915	12,591,024	0.05	LG-L
10	13,135,332	13,174,402	0.04	BW-L
10	13,364,868	13,558,518	0.05	LG-L
10	15,016,955	15,385,946	0.05	BW-L
10	15,017,526	15,038,968	0.05	LG-L
10	16,728,992	16,828,739	0.03	BW-L
10	16,749,702	16,770,696	0.21	LG-U
10	19,261,282	19,337,388	0.59	BW-U
11	40,717	240,158	0.21	LG-U
11	719,301	738,818	0.06	BW-L
11	1,511,157	1,546,605	0.06	BW-L
11	1,971,351	2,004,302	0.22	LG-U
11	2,160,518	2,198,778	0.05	LG-L
11	2,475,651	2,508,394	0.07	BW-L
11	3,399,240	3,957,997	0.03	LG-L
11	3,536,097	3,656,006	0.06	BW-L
11	4,404,436	4,461,344	0.54	BW-U
11	5,271,442	5,459,365	0.04	LG-L
11	5,273,655	5,287,126	0.07	BW-L
11	6,447,932	7,787,022	0.05	LG-L
11	6,711,298	6,737,456	0.05	BW-L
11	7,420,141	7,434,080	0.07	BW-L
11	9,958,471	11,856,923	0.64	BW-U
11	10,141,802	10,165,396	0.24	LG-U
11	10,389,169	10,413,329	0.08	BW-L
11	12,218,095	13,597,179	0.04	LG-L
11	12,419,681	12,731,242	0.66	BW-U
11	12,708,380	12,731,242	0.20	LG-U
11	12,728,008	13,783,420	0.01	BW-L

11	14,557,272	14,775,605	0.05	LG-L
11	14,962,152	14,983,836	0.54	BW-U
11	16,575,309	16,598,473	0.22	LG-U
11	16,577,128	16,596,724	0.51	BW-U
11	17,590,794	17,663,153	0.05	LG-L
11	17,732,646	17,782,166	0.64	BW-U
11	18,282,962	19,151,209	0.67	BW-U
11	18,779,388	19,150,008	0.22	LG-U
12	119,040	523,792	0.03	LG-L
12	122,874	164,864	0.05	BW-L
12	1,906,487	2,060,958	0.05	BW-L
12	1,908,738	2,064,123	0.05	LG-L
12	2,238,508	2,266,845	0.20	LG-U
12	3,451,706	3,645,802	0.06	BW-L
12	3,509,011	3,546,241	0.05	LG-L
12	3,771,722	3,841,240	0.26	LG-U
12	4,403,154	4,466,687	0.02	BW-L
12	4,690,825	5,044,996	0.20	LG-U
12	5,667,658	5,698,499	0.59	BW-U
12	5,667,658	6,733,716	0.24	LG-U
12	5,910,281	5,926,563	0.08	BW-L
12	7,317,340	7,332,162	0.05	LG-L
12	7,803,962	7,862,597	0.06	BW-L
12	8,368,655	8,382,746	0.20	LG-U
12	8,652,702	8,872,763	0.06	BW-L
12	8,986,336	9,007,583	0.22	LG-U
12	10,063,913	10,321,195	0.04	LG-L
12	10,160,209	10,209,630	0.06	BW-L
12	11,000,510	11,025,166	0.06	LG-L
12	12,294,963	12,313,153	0.06	BW-L
12	13,973,047	14,676,774	0.25	LG-U
12	14,035,519	14,686,314	0.56	BW-U
12	15,325,598	15,833,183	0.20	LG-U
12	17,401,984	17,418,141	0.05	LG-L
12	18,911,147	19,113,787	0.58	BW-U
13	234,276	262,443	0.04	LG-L
13	342,639	397,667	0.07	BW-L
13	441,255	475,699	0.20	LG-U
13	3,287,378	3,503,171	0.01	BW-L
13	3,564,294	3,856,190	0.22	LG-U
13	4,202,851	4,744,712	0.68	BW-U
13	4,401,836	4,625,853	0.05	LG-L
13	4,720,342	5,947,799	0.24	LG-U

13	5,266,290	5,970,819	0.72	BW-U
13	6,455,173	6,942,705	0.22	LG-U
13	6,761,239	6,780,079	0.05	BW-L
13	6,764,018	6,788,139	0.05	LG-L
13	8,125,844	8,545,656	0.24	LG-U
13	9,059,129	9,072,357	0.20	LG-U
13	10,739,070	10,959,942	0.24	LG-U
13	10,749,566	10,770,453	0.56	BW-U
13	11,817,905	11,967,748	0.57	BW-U
13	11,824,689	11,966,327	0.22	LG-U
13	13,015,277	13,076,973	0.06	BW-L
13	13,088,598	13,217,050	0.22	LG-U
13	13,247,762	13,266,104	0.53	BW-U
13	13,605,008	14,119,842	0.05	LG-L
13	16,765,338	16,812,700	0.57	BW-U
14	3,131,906	3,144,043	0.52	BW-U
14	4,751,385	4,762,906	0.05	LG-L
14	5,334,151	5,348,822	0.05	LG-L
14	6,592,604	6,612,979	0.60	BW-U
14	6,600,578	6,611,090	0.19	LG-U
14	8,339,375	8,380,144	0.05	BW-L
14	8,871,570	8,890,411	0.22	LG-U
14	8,931,595	9,210,963	0.08	BW-L
14	9,786,357	9,832,071	0.07	BW-L
14	9,950,461	9,962,966	0.05	LG-L
14	10,094,666	10,651,045	0.20	LG-U
14	10,672,789	11,047,682	0.05	LG-L
14	11,098,748	11,119,226	0.06	BW-L
14	11,301,673	11,768,724	0.55	BW-U
14	12,082,872	12,098,627	0.20	LG-U
14	13,480,437	13,492,784	0.08	BW-L
14	14,141,880	14,166,235	0.06	LG-L
14	14,565,105	14,588,046	0.64	BW-U
14	14,888,073	14,994,356	0.01	BW-L
14	15,068,865	15,079,586	0.06	LG-L
15	2,839,053	2,870,587	0.20	LG-U
15	2,839,053	3,216,526	0.59	BW-U
15	3,009,623	3,296,013	0.05	BW-L
15	3,856,434	4,284,717	0.64	BW-U
15	3,912,150	3,962,589	0.21	LG-U
15	4,175,622	4,197,555	0.05	LG-L
15	4,901,199	5,331,355	0.64	BW-U
15	4,902,360	6,633,772	0.27	LG-U

15	6,327,654	6,338,107	0.51	BW-U
15	7,688,594	8,012,867	0.23	LG-U
15	7,938,942	7,987,510	0.55	BW-U
15	11,230,733	11,603,526	0.21	LG-U
15	11,786,789	11,804,151	0.07	BW-L
16	216,322	455,298	0.04	LG-L
16	226,229	487,103	0.07	BW-L
17	76,944	443,272	0.08	BW-L
17	1,312,250	1,342,971	0.55	BW-U
17	1,802,893	2,280,597	0.03	BW-L
17	1,804,220	1,818,467	0.06	LG-L
17	2,321,217	2,360,843	0.04	LG-L
17	4,654,736	4,669,706	0.06	LG-L
17	5,213,727	5,994,107	0.04	LG-L
17	5,982,669	5,991,227	0.08	BW-L
17	6,764,700	7,875,530	0.04	LG-L
17	7,247,519	7,764,300	0.05	BW-L
17	9,399,900	9,422,487	0.20	LG-U
17	10,335,914	10,521,138	0.23	LG-U
17	10,449,724	10,505,360	0.55	BW-U
18	229,696	399,260	0.66	BW-U
18	2,375,733	2,663,264	0.20	LG-U
18	2,552,510	2,574,823	0.54	BW-U
18	4,286,374	4,303,003	0.06	LG-L
18	4,741,043	4,813,790	0.66	BW-U
18	6,055,628	6,498,422	0.05	BW-L
18	6,204,103	6,425,397	0.05	LG-L
18	6,921,224	7,300,510	0.65	BW-U
18	7,232,603	7,252,405	0.20	LG-U
18	7,749,977	8,107,403	0.07	BW-L
18	7,816,669	8,571,471	0.60	BW-U
18	10,113,905	10,913,287	0.04	BW-L
18	10,337,383	10,913,287	0.04	LG-L
19	1,014,177	1,257,871	0.20	LG-U
19	1,634,050	1,652,196	0.05	LG-L
19	1,636,792	1,651,799	0.08	BW-L
19	3,088,945	3,101,647	0.08	BW-L
19	4,068,455	4,086,377	0.54	BW-U
19	4,068,995	4,082,773	0.19	LG-U
19	4,472,108	5,015,459	0.05	LG-L
19	4,684,240	4,709,645	0.07	BW-L
19	6,776,994	6,798,020	0.57	BW-U
19	7,356,795	7,454,495	0.58	BW-U

19	8,059,065	8,072,964	0.57	BW-U
19	9,021,084	9,692,450	0.06	BW-L
19	9,204,093	9,247,281	0.05	LG-L
19	9,997,421	10,036,359	0.05	LG-L
20	596,479	1,695,129	0.67	BW-U
20	1,406,279	1,430,522	0.07	BW-L
20	1,544,547	1,648,728	0.22	LG-U
20	3,910,723	4,203,773	0.20	LG-U
20	4,190,510	4,205,386	0.52	BW-U
20	5,128,650	5,147,475	0.20	LG-U
20	5,398,811	5,418,338	0.08	BW-L
20	5,669,632	7,739,886	0.28	LG-U
20	5,694,658	7,883,011	0.76	BW-U
20	8,402,139	8,888,304	0.53	BW-U
20	8,660,115	8,678,680	0.20	LG-U
20	9,158,598	9,392,219	0.06	BW-L
20	9,821,452	9,925,236	0.05	LG-L
20	10,779,999	10,808,468	0.08	BW-L
20	10,877,639	10,895,105	0.51	BW-U
20	10,897,733	10,918,297	0.05	LG-L
20	12,630,029	12,648,306	0.06	LG-L, BW-L
20	13,125,673	13,145,449	0.63	BW-U
20	13,443,919	14,117,013	0.03	LG-L
20	13,559,577	14,110,878	0.04	BW-L
21	1,412,280	1,948,872	0.04	BW-L
21	1,414,066	1,773,511	0.04	LG-L
21	2,636,035	3,145,352	0.20	LG-U
21	3,120,503	3,143,971	0.52	BW-U
21	3,240,713	3,820,507	0.05	BW-L
21	3,409,989	3,433,368	0.04	LG-L
21	3,877,336	3,900,212	0.53	BW-U
21	4,696,605	5,074,167	0.63	BW-U
21	4,700,306	4,708,223	0.20	LG-U
21	5,604,707	5,618,687	0.57	BW-U
22	234,053	325,069	0.03	LG-L
22	246,914	313,138	0.00	BW-L
22	818,639	873,758	0.08	BW-L
22	1,546,280	2,239,440	0.07	BW-L
22	1,690,912	1,787,697	0.58	BW-U
22	1,763,070	1,782,072	0.21	LG-U
22	1,899,878	2,339,996	0.05	LG-L
22	3,400,232	3,632,706	0.01	BW-L
22	3,625,742	3,632,958	0.05	LG-L

23	70,130	163,504	0.05	LG-L
23	211,755	236,167	0.56	BW-U
23	833,995	946,265	0.60	BW-U
23	838,775	883,063	0.20	LG-U
23	3,063,562	5,253,343	0.04	LG-L
23	3,506,566	3,526,024	0.06	BW-L
23	4,160,011	5,286,741	0.06	BW-L
24	16,009	665,935	0.04	LG-L
24	32,849	178,257	0.07	BW-L
24	1,100,667	1,148,998	0.55	BW-U
24	2,639,395	2,647,933	0.08	BW-L
24	3,554,559	3,579,220	0.53	BW-U
24	3,561,409	3,579,220	0.19	LG-U
24	3,843,336	3,856,474	0.05	LG-L
24	3,982,629	3,993,700	0.08	BW-L
24	4,478,106	4,504,047	0.05	LG-L
24	5,321,343	6,279,737	0.05	BW-L
24	5,817,540	5,912,543	0.05	LG-L
24	6,158,505	6,194,686	0.54	BW-U
25	1,012,824	1,177,961	0.24	LG-U
25	1,130,229	1,144,719	0.05	BW-L
25	1,164,079	1,177,961	0.52	BW-U
25	1,525,469	2,103,505	0.04	LG-L
25	1,694,849	1,810,947	0.07	BW-L
26	8,294	289,726	0.02	LG-L
26	68,665	280,290	0.03	BW-L
26	1,341,969	1,811,160	0.06	LG-L
26	2,370,211	2,808,626	0.05	LG-L
26	3,221,916	3,314,291	0.21	LG-U
26	3,410,677	3,687,149	0.52	BW-U
26	4,961,676	5,071,189	0.05	LG-L
27	3,594	1,112,116	0.02	LG-L
27	3,594	1,205,084	0.03	BW-L
27	1,770,715	1,785,086	0.07	BW-L
27	2,173,155	2,907,109	0.05	LG-L
27	2,391,486	2,404,523	0.07	BW-L
27	4,599,444	5,184,681	0.03	LG-L
27	4,608,449	5,180,934	0.04	BW-L
28	449,850	951,082	0.05	BW-L
28	507,443	518,782	0.06	LG-L
28	1,591,032	1,996,687	0.05	BW-L
28	1,639,079	2,030,272	0.04	LG-L
28	2,607,017	2,621,935	0.05	LG-L

28	3,609,562	3,634,986	0.06	BW-L
28	3,609,562	3,786,389	0.05	LG-L
40	156,197	174,988	0.04	LG-L
40	157,443	265,926	0.06	BW-L
40	813,141	856,880	0.05	LG-L
41	95,135	405,077	0.04	LG-L
Z	925,002	959,914	0.27	LG-U
Z	3,087,524	3,124,257	0.07	LG-L
Z	9,075,584	9,923,249	0.06	LG-L
Z	9,077,792	9,101,549	0.07	BW-L
Z	10,063,235	10,271,603	0.29	LG-U
Z	11,595,755	12,205,089	0.00	BW-L
Z	12,052,641	12,604,587	0.05	LG-L
Z	13,877,639	13,932,821	0.29	LG-U
Z	15,469,173	16,239,232	0.05	LG-L
Z	15,472,480	15,922,582	0.06	BW-L
Z	16,944,353	16,975,567	0.07	LG-L
Z	17,157,214	17,202,688	0.31	LG-U
Z	17,242,521	17,271,710	0.07	BW-L
Z	17,869,228	17,941,316	0.32	LG-U
Z	18,685,214	19,223,959	0.05	BW-L
Z	18,783,898	18,814,715	0.28	LG-U
Z	19,343,727	19,499,379	0.30	LG-U
Z	20,033,586	20,208,207	0.01	BW-L
Z	21,623,129	21,648,745	0.07	BW-L
Z	22,002,694	22,024,547	0.28	LG-U
Z	22,071,564	22,095,330	0.80	BW-U
Z	22,834,213	22,879,298	0.02	BW-L
Z	23,912,936	23,942,979	0.28	LG-U
Z	25,082,461	25,104,891	0.79	BW-U
Z	25,638,064	26,752,468	1.00	BW-U
Z	26,649,096	26,770,218	0.28	LG-U
Z	32,422,731	32,454,768	0.28	LG-U
Z	33,218,363	33,282,384	0.84	BW-U
Z	35,691,090	35,922,554	0.86	BW-U
Z	36,065,884	36,604,428	0.04	BW-L
Z	38,395,052	40,008,371	0.33	LG-U
Z	38,469,345	40,008,371	0.95	BW-U
Z	40,637,636	40,685,742	0.06	BW-L
Z	40,880,276	40,970,302	0.89	BW-U
Z	42,207,351	46,282,225	1.00	BW-U
Z	44,189,764	44,217,864	0.00	BW-L
Z	44,191,555	44,211,186	0.07	LG-L

Z	44,485,699	44,653,110	0.31	LG-U
Z	45,942,267	46,278,712	0.31	LG-U
Z	46,363,571	46,483,688	0.00	BW-L
Z	46,451,947	46,479,545	0.07	LG-L
Z	47,453,819	47,497,982	0.86	BW-U
Z	47,458,699	47,492,507	0.29	LG-U
Z	48,608,945	49,004,990	0.00	BW-L
Z	48,821,026	48,894,253	0.06	LG-L
Z	49,336,598	49,402,049	0.31	LG-U
Z	51,433,170	51,453,731	0.06	BW-L
Z	51,570,218	52,025,021	0.30	LG-U
Z	53,396,549	53,613,767	0.29	LG-U
Z	54,396,787	54,415,100	0.00	BW-L
Z	54,514,962	54,924,783	0.82	BW-U
Z	54,527,007	54,969,616	0.31	LG-U
Z	55,072,139	55,144,749	0.00	BW-L
Z	55,084,066	55,145,974	0.05	LG-L
Z	55,747,328	55,793,032	0.06	LG-L
Z	55,918,219	55,984,468	0.28	LG-U
Z	57,959,535	58,034,588	0.89	BW-U
Z	60,962,983	60,985,386	0.81	BW-U
Z	61,757,609	62,368,193	0.31	LG-U
Z	62,310,380	62,362,213	0.79	BW-U
Z	62,448,231	62,546,884	0.02	BW-L
Z	63,170,909	63,197,666	0.30	LG-U
Z	63,590,847	64,066,815	0.93	BW-U
Z	63,645,413	63,963,994	0.00	BW-L
Z	63,877,426	63,964,530	0.05	LG-L
Z	64,464,665	64,899,635	0.29	LG-U
Z	65,660,899	65,801,895	0.87	BW-U
Z	66,255,023	66,281,778	0.29	LG-U
Z	67,575,161	67,621,643	0.01	BW-L
Z	67,876,700	67,908,061	0.81	BW-U
Z	68,141,546	69,569,848	0.00	BW-L
Z	68,162,306	68,433,138	0.05	LG-L
Z	68,841,648	68,872,079	0.28	LG-U
Z	69,068,804	69,564,869	0.01	LG-L
Z	71,047,809	71,070,199	0.79	BW-U
Z	72,061,998	72,081,848	0.07	BW-L
Z	72,782,943	72,802,648	0.07	LG-L
Z	81,327,880	81,424,699	0.06	LG-L
Z	81,934,185	81,943,896	0.07	LG-L
Z	82,203,476	82,255,866	0.86	BW-U
Z	83,348,332	83,426,842	0.28	LG-U
